# Small-scale photonic Kolmogorov-Arnold networks using standard telecom nonlinear modules

**DOI:** 10.1038/s41467-026-75602-8

**Published:** 2026-07-21

**Authors:** Luca Nogueira Calçado, Sergei K. Turitsyn, Egor Manuylovich

**Affiliations:** Aston Institute of Photonic Technologies (AiPT), Aston University, Birmingham, UK

**Keywords:** Fibre optics and optical communications, Nonlinear optics, Electrical and electronic engineering

## Abstract

Photonic neural networks promise inference at the speed of light, yet most architectures combine linear optical meshes with electronic nonlinearities, reintroducing optical-electrical-optical bottlenecks. Kolmogorov-Arnold networks place trainable nonlinear functions on network edges, concentrating expressivity into a few structured modules. Each edge here is a single module built from a Mach-Zehnder interferometer, a semiconductor optical amplifier, and variable optical attenuators, giving a four-parameter transfer function set by gain saturation and interferometric mixing. A four-module network attains 94.3% accuracy (±3.9% s.d. over ten seeds) on nonlinear classification, and a seven-module network reaches *R*^2^ = 0.986 ± 0.015 on six-input regression, remaining robust to 6-bit inputs and 14 dB signal-to-noise ratio. Here, we show that a fully differentiable physics model enables end-to-end optimization of these standard telecom modules, giving a practical route from simulation toward experimental demonstration of photonic Kolmogorov-Arnold networks.

## Abstract (web summary)

Photonic neural networks usually rely on power-hungry optical-electronic conversions for nonlinearity. Here, authors build a Kolmogorov-Arnold network whose every edge is one telecom module-an interferometer, amplifier and attenuators-trained end-to-end with a differentiable physics model.

## Introduction

As neural networks become increasingly embedded within optical technologies - from high-capacity communications and information processing to advanced imaging and sensing (see e.g.,^1–9^ and references therein) - there is rising demand for inference hardware capable of matching the speed and bandwidth of light itself. Although photonic neural networks (PNNs) promise orders-of-magnitude improvements in latency and energy efficiency by performing computation directly in the optical domain, most existing implementations follow the conventional multilayer perceptron (MLP) paradigm. In these architectures, linear optical matrix-vector multiplication, implemented using interferometric meshes or wavelength multiplexing, is combined with electronic nonlinear activation functions. This hybrid approach reintroduces optical-electrical-optical (OEO) conversion bottlenecks, thereby limiting scalability and offsetting many of the anticipated advantages of all-optical processing^10,11^. Even high-performance accelerators achieving 11 TOPS throughput^12^ and parallel convolutional processing on photonic tensor cores^13,14^ rely on electronic nonlinearities between optical layers.

Kolmogorov-Arnold Networks (KANs) offer a fundamentally different computation paradigm^15^. Inspired by the Kolmogorov-Arnold representation theorem, KANs depart from the conventional node-based activation model by placing trainable univariate nonlinear functions on network edges. In contrast to multilayer perceptrons (MLPs), where fixed activation functions follow linear transformations, KANs distribute nonlinearity across connections, restructuring the computational graph itself. Recent work has demonstrated that this architecture can achieve accuracy comparable to, and in some cases exceeding, that of MLPs while requiring substantially fewer trainable parameters. Moreover, the learned edge functions admit symbolic approximation, enhancing interpretability and analytical transparency^15^.

Recent results in short-reach IM/DD optical communication systems show that KAN-based equalizers can achieve superior or comparable BER performance while using substantially fewer trainable parameters than conventional FNN and CNN architectures, demonstrating reductions of up to  ~66%^16^. These properties are particularly attractive for photonic implementation, where each nonlinear element incurs optical loss, hardware complexity, and fabrication overhead. By concentrating expressive power into a reduced number of structured nonlinear modules, KANs provide a parameter-efficient and physically compatible framework for scalable optical neural computation.

Several recent advances provide strong motivation for photonic implementations of KANs. Fischer et al.^17^ applied software-based KANs to nonlinear equalization in 112 Gb/s passive optical networks, demonstrating that KAN equalizers outperform convolutional neural networks at comparable computational complexity. These results show that KAN architectures are effective for high-speed optical signal processing and motivate their direct realization in the optical domain. Peng et al.^18^ reported the first photonic KAN implementation using ring-assisted Mach-Zehnder interferometers (RAMZI), using free-carrier dispersion in silicon microring resonators to create tunable nonlinear transfer functions. Their integrated silicon photonic architecture achieved 98% accuracy on MNIST handwritten digit recognition while reducing the energy-area product by approximately 65-fold compared to conventional MZI-based optical neural networks.

More recently, Stroev and Berloff^19^ introduced a theoretical framework for all-optical KANs implemented on spatial light modulator platforms, showing that trainable univariate nonlinear functions can arise from structural interference effects without relying on conventional nonlinear optical materials.

Despite these advances, two fundamental challenges remain. First, it is unclear whether the constrained nonlinear transfer functions available from practical optical components (often defined by only a small number of tunable physical parameters) can achieve expressive capability comparable to software-based activation functions such as B-splines, which may involve hundreds of learnable coefficients per edge. Second, and more critically for real-world deployment, it remains an open question whether photonic KANs can be implemented using off-the-shelf telecommunications components that are mass-produced, well-characterized, and optimized for the spectral bands and power levels of fiber-optic systems.

Conventional photonic neural networks face related but distinct constraints: photonic multilayer perceptrons perform linear matrix-vector multiplication optically but require electronic nonlinear activations between layers, while photonic reservoir computers exploit intrinsic nonlinear dynamics but use fixed, untrained nonlinearities with only a linear readout optimized. Previous photonic KAN demonstrations have relied on specialized resonator geometries or engineered nonlinear waveguides.

SSP-KAN differs from prior photonic KAN architectures in hardware accessibility. The RAMZI approach^18^ requires custom silicon photonic fabrication with precise resonance tuning; the SPM-based design^20^ requires engineered nonlinear waveguides tailored at the foundry level. SSP-KAN uses only commercially available, fiber-pigtailed telecom components and needs no custom fabrication. The nonlinearity is SOA gain saturation, a broadband traveling-wave effect that operates across the full C-band. Embedding the SOA within an MZI adds an interferometric phase degree of freedom; the resulting four-parameter module produces a family of nonlinear transfer functions from saturable gain combined with interferometric mixing. This per-edge trainability is what enables compositional depth to compensate for the limited expressivity of each individual module, while the use of commodity telecom hardware lowers the barriers to experimental realization.

Here we demonstrate that physically constrained photonic nonlinearities can act as trainable activation functions within Kolmogorov-Arnold Networks, enabling nonlinear inference using standard telecommunications components. Specifically, we show that Mach-Zehnder interferometers combined with semiconductor optical amplifiers and variable optical attenuators (MZI-VOA-SOA-VOA modules) can function as compact, trainable nonlinear units for photonic KANs, with each module controlled by only four physical parameters. Our approach differs from prior implementations based on specialized resonator geometries or custom optical platforms, in two key aspects: (i) it uses SOA gain saturation as a primary (native to telecom) nonlinearity that does not require bespoke fabrication; (ii) the nonlinear phase shift from SOA amplification is employed as a source of nonlinearity by placing the SOA inside an MZI. We have developed a fully differentiable physics-based model that enables end-to-end gradient optimization of all optical parameters, including injection current, attenuation coefficients, and interferometric phase.

Using commercially specified SOA parameters (Thorlabs BOA1554P), a four-module [2,2] SSP-KAN achieves 94.3% test accuracy (±3.9% s.d., 10 seeds) on Two Moons nonlinear classification, and a seven-module [6,1,1] network attains *R*^2^ = 0.986 ± 0.015 on six-input yacht hydrodynamics regression. Scaling studies on MNIST image classification further demonstrate competitive performance, reaching 93.9 ± 0.2% accuracy (10 seeds). Systematic robustness analysis under optical noise and finite-resolution input quantization confirms stable operation across realistic impairment regimes.

Overall, we introduce a SSP-KAN design framework grounded entirely in standard telecommunications components. The approach is inherently modular and can be extended to alternative nonlinear devices and photonic architectures, providing a foundation for telecom-grade implementations of trainable optical neural networks. Rather than pursuing asymptotic universality, this work demonstrates that physically constrained, low-parameter photonic KANs are sufficient for meaningful nonlinear inference in realistic settings. Such architectures are particularly well suited to structured computational tasks, including physics-informed modeling, low-dimensional regression, symbolic discovery, latency-critical edge inference, and applications where interpretability and hardware efficiency are paramount.

## Results

### SSP-KAN architecture

**Fig. 1:**
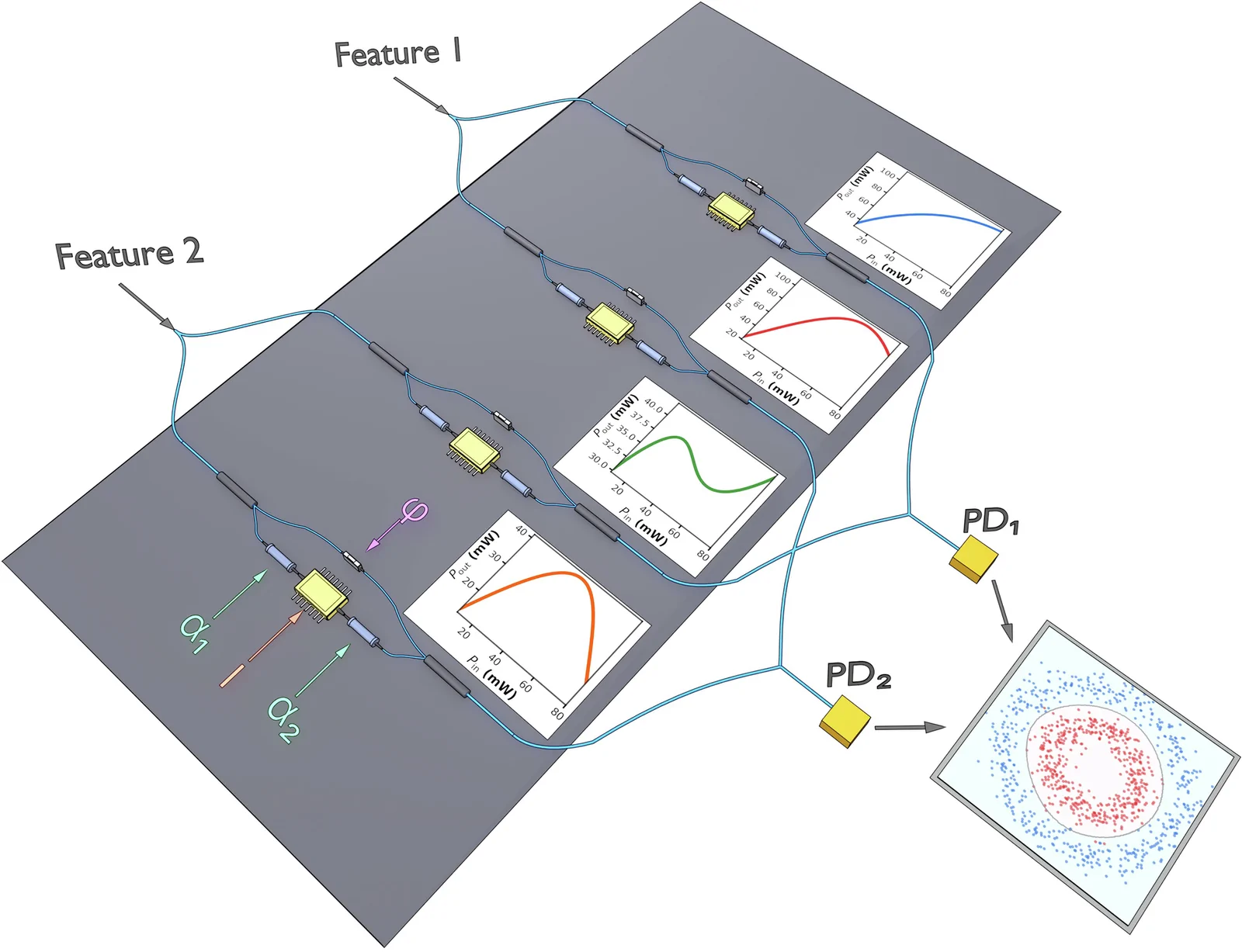
SSP-KAN [2,2] network. Two input features are encoded as optical power and split to four MZI-VOA-SOA-VOA modules on a fiber-coupled breadboard. Each module (detail, lower left) has four trainable parameters: input attenuation *α*_1_, injection current *I*, output attenuation *α*_2_, and interferometric phase *ϕ*. Insets show the learned transfer function *P*_out_(*P*_in_) of each module. The per-edge outputs are summed on two photodetectors (PD_1_, PD_2_), producing the nonlinear decision boundary on the concentric rings task (right).

The main idea of SSP-KAN is to replace software-defined activation functions with physically realized optical transfer functions, placing a dedicated MZI-VOA-SOA-VOA module on each network edge. Figure 1 shows a minimal [2,2] network with two input nodes and two output nodes, requiring four MZI-VOA-SOA-VOA modules. Each output is computed as a sum of the nonlinearly-transformed inputs:

1 $${y}_{j}={\sum}_{i=1}^{{n}_{{{{\rm{in}}}}}}{\varphi }_{i,j}({x}_{i})$$

where *φ*_*i*,*j*_ represents the transfer function of the module on edge (*i*, *j*). This is the key structural difference from multilayer perceptrons: rather than applying a fixed nonlinearity after a weighted sum, each edge independently transforms its input through a physically distinct optical transfer function. The summation itself can be performed through incoherent power combining on a photodetector, requiring no electronic computation within the network.

Each module (detail in Fig. 1) consists of a Mach-Zehnder interferometer with a semiconductor optical amplifier and variable optical attenuators in one arm, providing a tunable nonlinear transfer function controlled by four trainable parameters. The SOA injection current *I* sets both the gain magnitude and the degree of saturation nonlinearity, ranging from approximately linear response at low currents to pronounced gain compression at high currents. The input attenuation *α*_1_ controls the optical power entering the SOA, thereby setting the operating point on the gain saturation curve: low attenuation drives the SOA into saturation with strong nonlinearity, while high attenuation keeps it in the linear amplification regime. The output attenuation *α*_2_ scales the amplified arm contribution to the interference without affecting the saturation dynamics. The interferometer phase *ϕ* selects which portion of the MZI cosine-squared fringe pattern reaches the output, choosing between constructive and destructive interference regimes. Together, these four parameters per module define a family of transfer functions that spans a rich space of nonlinear input-output relationships (insets in Fig. 1). All simulations use manufacturer-specified parameters for a commercially available SOA (Thorlabs BOA1554P; see Methods for the full physical model).

### Nonlinear classification on Two Moons

To establish that SSP-KAN can learn nonlinear decision boundaries, we evaluate on the Two Moons dataset, a standard binary classification benchmark where two interleaving half-circles cannot be separated by any linear classifier. The dataset comprises 1000 training and 1000 test samples with Gaussian noise (*σ* = 0.1), scaled to the optical power range 10–80 mW. We train a single-layer [2,2] network comprising 4 MZI-VOA-SOA-VOA modules (16 trainable parameters) and compare against a linear baseline and a software KAN baseline implemented using pyKAN^15^ with grid size *G* = 1 and B-spline activation functions.

**Fig. 2:**
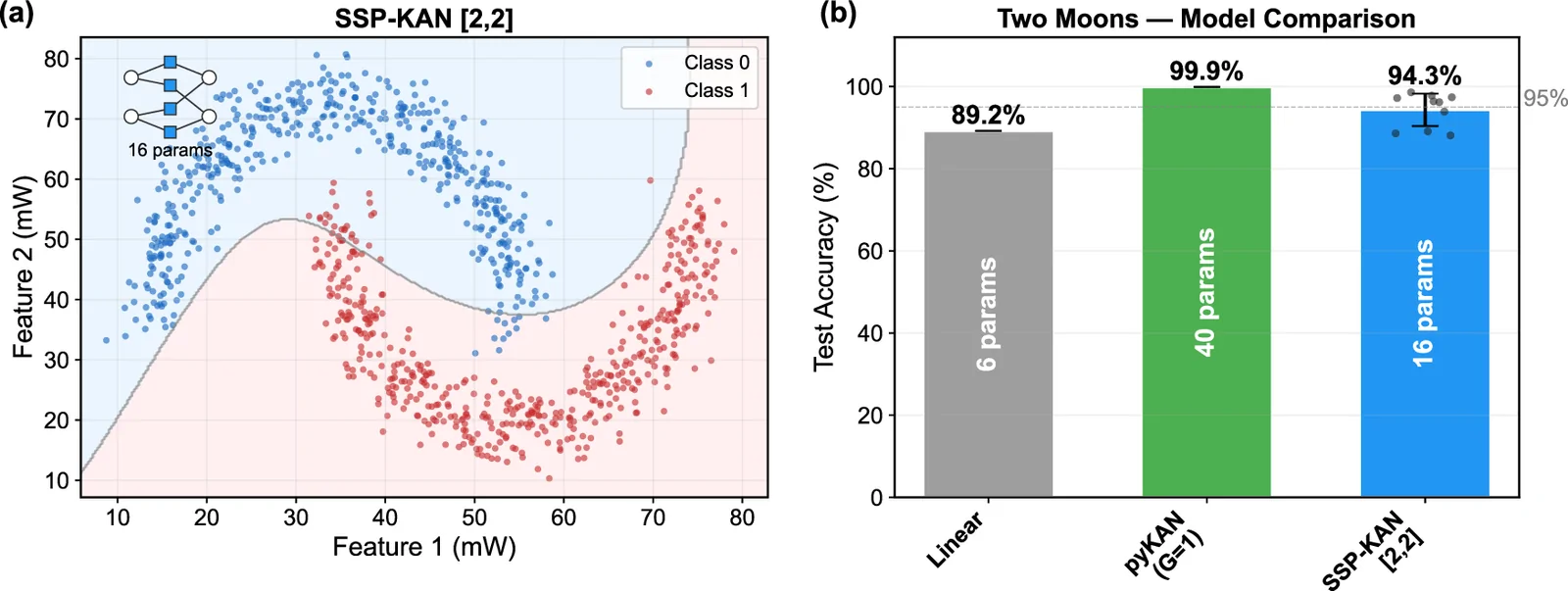
Two Moons classification with SSP-KAN [2,2]. **a** Decision boundary for a representative seed; the network achieves 94.3% (±3.9% s.d.) test accuracy over 10 seeds with 16 parameters. The boundary separates the two crescents using a smooth nonlinear function generated by four MZI-VOA-SOA-VOA modules. **b** Test accuracy and parameter count across models. The linear baseline (89.2%, 6 parameters) confirms the task is intrinsically nonlinear. pyKAN (*G* = 1, 40 parameters) reaches 99.9%; SSP-KAN achieves 94.3% with 16 parameters using physically realizable optical activation functions. For the SSP-KAN [2,2] bar: points, individual seeds (*n* = 10); bar, mean; error bars, s.d. (*n* = 10). Linear and pyKAN are single values. Inset: [2,2] network schematic showing 2 inputs, 4 modules, and 2 outputs.

Figure 2 a shows the learned decision boundary for the [2,2] architecture. The network achieves 94.3% (±3.9% s.d., 10 seeds) test accuracy, separating the two crescents with a smooth nonlinear boundary that follows the data geometry. The linear baseline reaches only 89.2% (Fig. 2b), confirming the intrinsically nonlinear nature of the task. The software KAN baseline (pyKAN, *G* = 1) achieves 99.9% with 40 parameters; SSP-KAN closes to within 5.6 percentage points using 16 parameters and a transfer function family constrained to the physics of MZI-VOA-SOA-VOA modules. This accuracy gap reflects that the nonlinear response of saturated semiconductor optical amplifiers, shaped by the four trainable parameters per edge, provides sufficient functional diversity to approximate the B-spline activation functions used in software KANs, though the periodic MZI transfer function creates multiple local minima that widen the gap relative to single-seed results (best seed: 98.6%, worst: 88.1%). A deeper [2,2,2] architecture with 8 modules substantially improves accuracy to 99.1% (±1.2% s.d., 10 seeds) (Supplementary Note 1), demonstrating that compositional depth compensates for limited per-module expressivity. Multi-layer operation introduces coherent phase accumulation at intermediate summation nodes, which is analyzed in Supplementary Note 2.

**Fig. 3:**
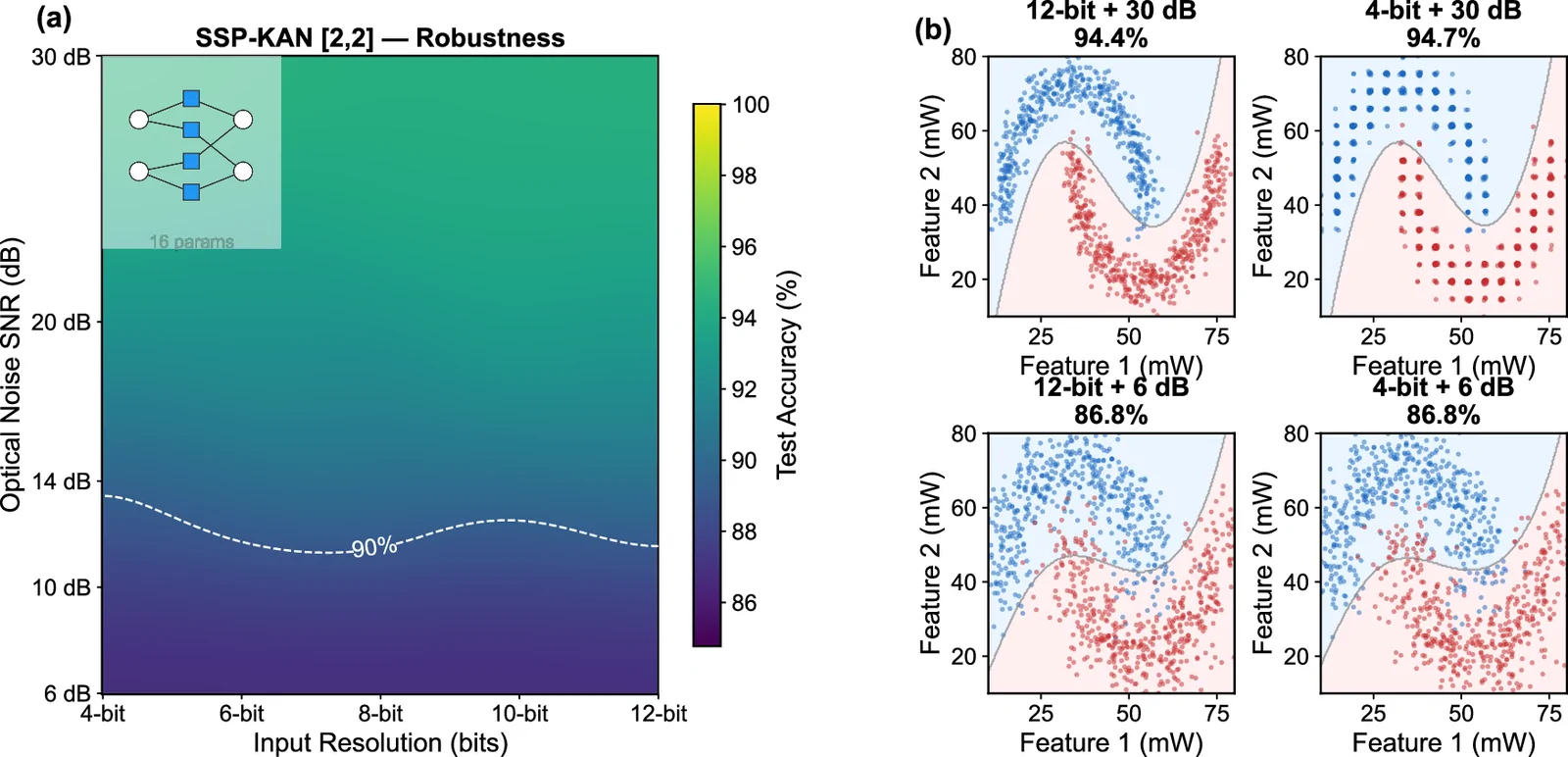
Hardware robustness of SSP-KAN [2,2] on Two Moons. **a** Test accuracy as a function of input DAC resolution (12-bit to 4-bit) and optical noise (SNR 30 dB to 6 dB). The smooth color gradient confirms that quantization has negligible impact, while performance degrades vertically with decreasing SNR. The 90% and 95% contour lines indicate the practical operating boundary. Inset: [2,2] network schematic (16 parameters). **b** Decision boundaries at four corner conditions. Top row: 30 dB noise at 12-bit (94.4%) and 4-bit (94.7%) resolution. Bottom row: 6 dB noise at 12-bit (86.8%) and 4-bit (86.8%). The boundary shape is preserved across quantization levels but simplifies under severe noise.

Having established classification performance under ideal conditions, we next evaluate robustness to the two dominant hardware impairments in photonic systems: finite digital-to-analog converter (DAC) resolution for input encoding and optical noise from amplified spontaneous emission (ASE). Figure 3a shows classification accuracy for the [2,2] network across input quantization levels (12-bit down to 4-bit) and optical SNR conditions (30 dB down to 6 dB). Quantization has negligible effect on performance: at any given SNR, accuracy is nearly constant across all tested resolutions. This implies that inexpensive, low-resolution DACs are sufficient for input encoding.

Optical noise, by contrast, produces measurable degradation. At SNR = 30 dB accuracy is approximately 94%, dropping to 92% at 14 dB, and 90% at 10 dB. Under extreme noise (SNR = 6 dB), the network retains approximately 87% accuracy, falling below the linear baseline (89.2%). At sufficiently high noise levels the nonlinear decision boundary becomes a liability: the optimizer converges to a coarser separation that is less effective than a simple linear cut. The decision boundaries under extreme conditions (Fig. 3b) illustrate this directly. Under 6 dB noise, the boundary approximates a broad diagonal rather than tracking the crescent geometry. This crossover defines a practical operating threshold for the [2,2] architecture: for SNR ≥ 10 dB, the nonlinear model consistently outperforms the linear baseline, while below 10 dB, simpler models may be preferable. Figure 3b confirms the quantization finding: the 4-bit boundary (86.8%) is visually and numerically indistinguishable from the 12-bit boundary (86.8%) at the same noise level. For realistic deployment conditions (8-bit DAC, SNR ≥ 14 dB), the [2,2] architecture maintains accuracy above 91%, comfortably exceeding the linear baseline.

### Regression performance on yacht hydrodynamics

To evaluate SSP-KAN on a higher-dimensional task, we use the UCI Yacht Hydrodynamics dataset^21^, derived from Delft ship hydrodynamics experiments^22^. The dataset contains 308 samples, each described by six hull geometry features (longitudinal position of the center of buoyancy, prismatic coefficient, length-displacement ratio, beam-draught ratio, length-beam ratio, and Froude number) with the prediction target being residuary resistance per unit weight of displacement. This regression task tests whether SSP-KAN can learn complex multivariate functions from six-dimensional inputs using a small number of optical modules.

**Fig. 4:**
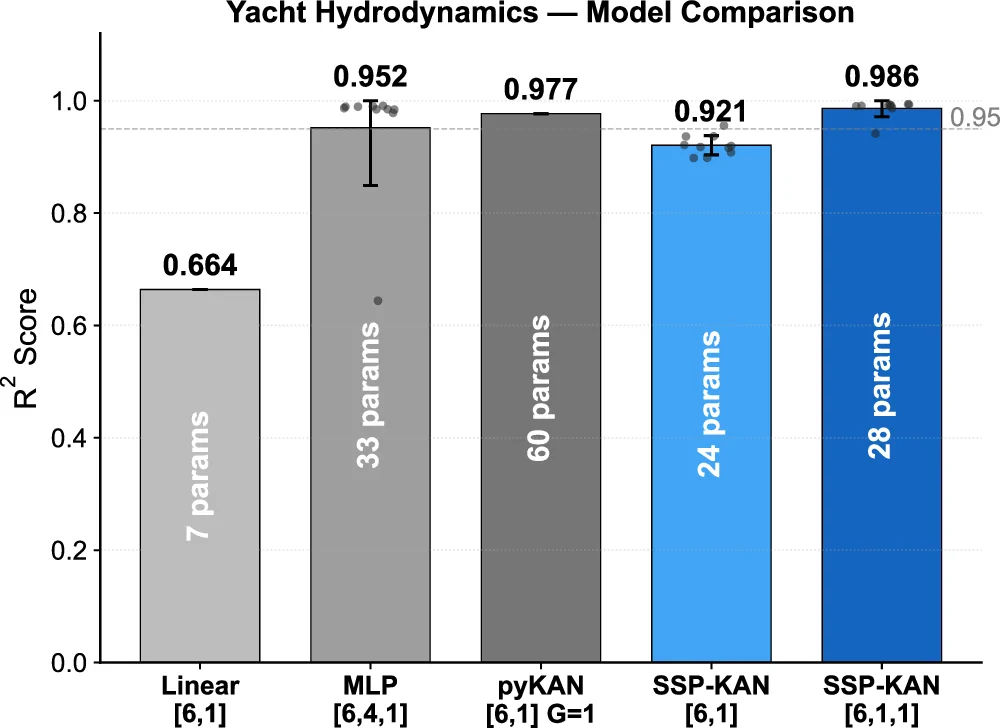
Regression performance on yacht hydrodynamics. Test *R*^2^ scores and parameter counts for linear regression, MLP, software KAN (pyKAN, *G* = 1), and SSP-KAN architectures [6,1] and [6,1,1]. For the multi-seed bars (MLP, SSP-KAN [6,1], SSP-KAN [6,1,1]): points, individual seeds (*n* = 10); bar, mean; error bars, s.d. Linear and pyKAN are single values. SSP-KAN [6,1,1] exceeds pyKAN (*R*^2^ = 0.977) with 28 parameters versus 60, demonstrating that compositional depth compensates for the constrained per-edge activation family.

We evaluate two architectures: a single-layer [6,1] network with 6 MZI-VOA-SOA-VOA modules (24 trainable parameters) and a two-layer [6,1,1] network with 7 modules (28 parameters). Figure 4 compares both against baseline models. A linear regression baseline achieves *R*^2^ = 0.664, confirming the nonlinear nature of the hull-resistance relationship. A multilayer perceptron (MLP) with architecture [6→4→1] and 33 parameters achieves *R*^2^ = 0.952 ± 0.103 (10 seeds), while a software KAN (pyKAN, [6,1] with grid size *G* = 1, 60 parameters) reaches *R*^2^ = 0.977. The single-layer SSP-KAN [6,1] achieves *R*^2^ = 0.921 ± 0.017 (10 seeds): a substantial improvement over the linear baseline, but limited by the fact that each of its six modules computes a univariate function of a single input feature, with no capacity for feature interaction. Adding a second layer improves performance dramatically: SSP-KAN [6,1,1] reaches *R*^2^ = 0.986 ± 0.015, exceeding pyKAN while using fewer than half the parameters (28 vs 60). The second-layer module composes the six first-layer outputs into a single nonlinear function, enabling the network to capture multivariate interactions that no sum of univariate functions can represent.

**Fig. 5:**
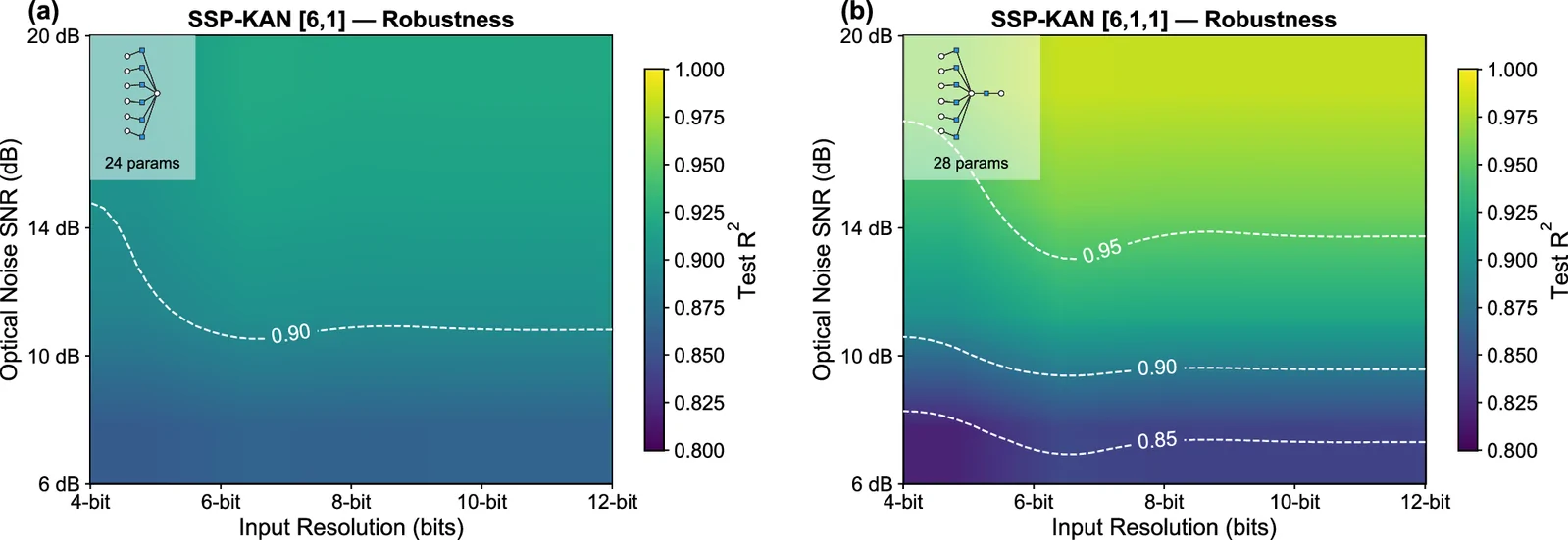
Hardware robustness of SSP-KAN on yacht hydrodynamics regression. Test *R*^2^ as a function of input DAC resolution (12-bit to 4-bit) and optical noise (SNR 20 dB to 6 dB) for **a** the single-layer [6,1] architecture (6 modules, 24 parameters) and **b** the two-layer [6,1,1] architecture (7 modules, 28 parameters). Contour lines mark *R*^2^ thresholds. The [6,1] panel shows a compressed performance range (*R*^2^ ≈ 0.86–0.92), while [6,1,1] spans a wider range (*R*^2^ ≈ 0.82–0.99), reflecting both higher peak performance and greater noise sensitivity.

Robustness to hardware impairments is assessed across SNR levels from 6 to 20 dB and DAC resolutions from 4-bit to 12-bit (Fig. 5). Figure 5a, b shows *R*^2^ as a function of input resolution at each noise level for the [6,1] and [6,1,1] architectures, respectively. As with Two Moons classification, input quantization has minimal effect: for the [6,1,1] architecture at SNR = 20 dB, *R*^2^ remains between 0.98 and 0.99 from 12-bit down to 8-bit, with a decrease to *R*^2^ ≈ 0.96 at 4-bit. Optical noise produces more significant degradation, reducing [6,1,1] performance from *R*^2^ ≈ 0.98 at SNR = 20 dB to approximately 0.96 at 14 dB, 0.92 at 10 dB, and 0.84 at 6 dB.

Figure 5 shows a clear contrast between the two panels. The [6,1] architecture (Fig. 5a) shows performance compressed into a narrow range between *R*^2^ ≈ 0.86 and 0.92 across the entire parameter space. The [6,1,1] architecture (Fig. 5b) spans a much wider range, from *R*^2^ ≈ 0.99 at 20 dB down to 0.82 at 6 dB, with contour lines indicating where each performance threshold is crossed. This contrast directly visualizes the depth-robustness trade-off: the deeper network achieves substantially higher performance under mild impairment but is more sensitive to perturbation, as the second-layer module amplifies noise that has already propagated through the first layer. The shallower [6,1] network, by contrast, has less capacity to lose. Under extreme noise (6 dB), the [6,1] network (*R*^2^ ≈ 0.86) outperforms [6,1,1] (*R*^2^ ≈ 0.82), indicating that the deeper architecture’s greater noise sensitivity outweighs its capacity advantage at this SNR. Under combined impairments representative of realistic deployment (8-bit DAC, SNR = 14 dB), the [6,1,1] architecture maintains *R*^2^ ≈ 0.96, well above the linear baseline (*R*^2^ = 0.664). A mild increase in quantization sensitivity is visible at 4-bit for the [6,1,1] network under lower SNR conditions, suggesting that 6-bit DAC resolution represents a practical lower bound for the deeper architecture.

### Image classification on MNIST and Fashion-MNIST

To evaluate SSP-KAN on high-dimensional inputs representative of practical inference tasks, we tested on MNIST handwritten digit classification (784-dimensional input, 10 classes) and Fashion-MNIST clothing classification (same dimensionality, 10 classes). MNIST is a direct comparison point with the D-RAMZI photonic KAN of Peng et al.^18^, while also testing whether the MZI-VOA-SOA-VOA transfer function can handle image-scale inputs. Fashion-MNIST is a substantially harder benchmark: its classes share more visual similarity (e.g., shirts, coats, and pullovers occupy overlapping regions of pixel space), making it a more demanding test of whether optical nonlinearity provides meaningful benefit over linear classification.

We evaluated three SSP-KAN architectures of increasing capacity: a single-layer [784,10] network with 7840 MZI-VOA-SOA-VOA modules, a two-layer [784,10,10] network with 7940 modules, and a wider two-layer [784,20,10] network with 15,880 modules. All models were trained with identical hyperparameters (AdamW optimizer, weight decay 10^−3^, 150 epochs; see “Methods”). A linear classifier trained under the same conditions is the baseline, isolating the contribution of optical nonlinearity from the effect of increased model capacity.

**Fig. 6:**
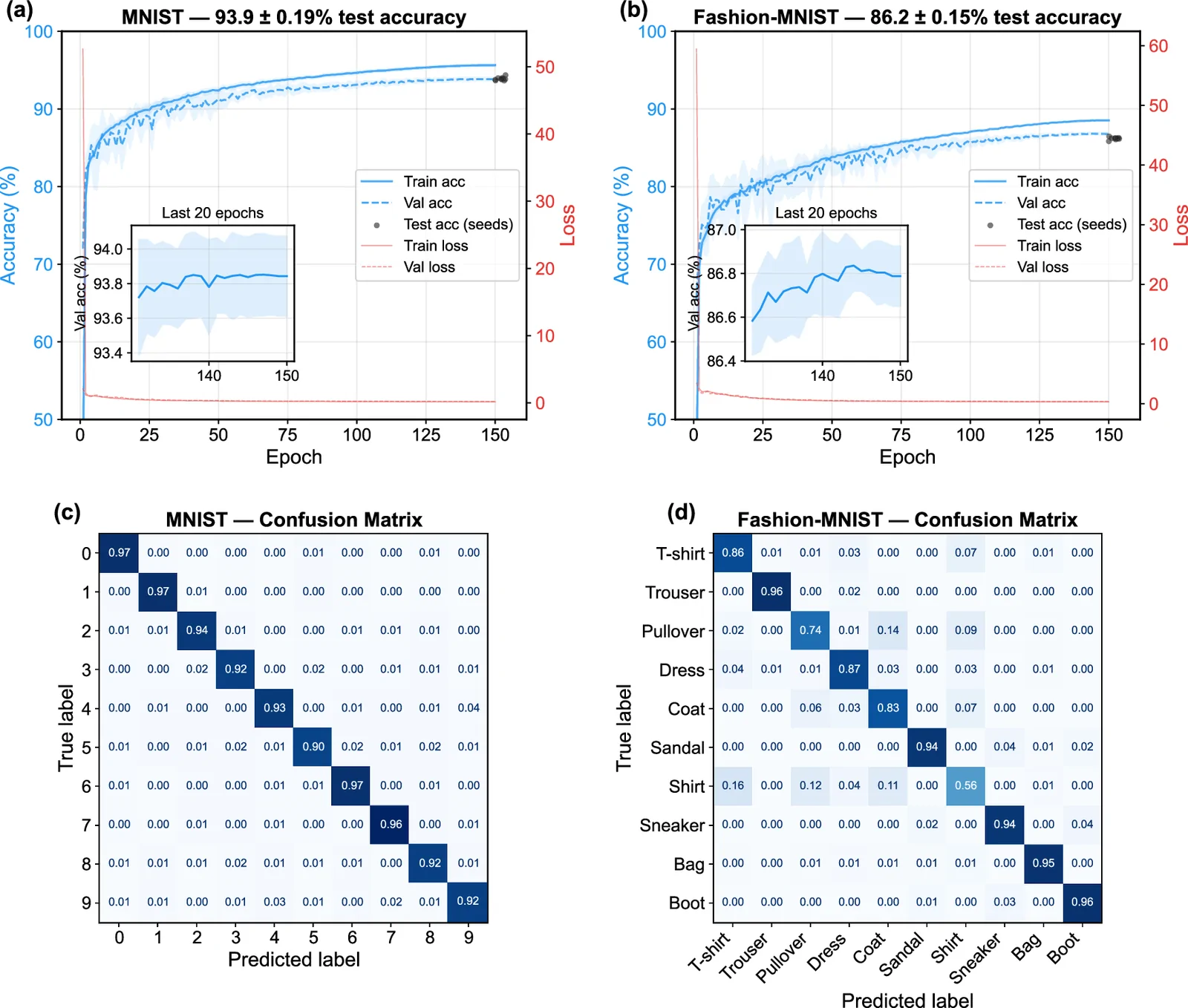
Image classification results. Training curves showing accuracy and loss for **a** MNIST and **b** Fashion-MNIST using the [784,20,10] architecture. Curves show the mean over 10 seeds and shaded bands the s.d.; points at the right edge of each panel are the individual per-seed final test accuracies (*n* = 10). Insets show the final training epochs at expanded scale. Confusion matrices for the [784,20,10] model on **c** MNIST and **d** Fashion-MNIST, showing that classification errors concentrate among visually similar classes.

On MNIST, the linear baseline achieves 91.0% accuracy, consistent with the well-known near-linear separability of handwritten digits in pixel space. SSP-KAN [784,20,10] reaches 93.9 ± 0.2% (10 seeds), a gain of 2.9 percentage points over the linear model (Fig. 6). On Fashion-MNIST, where the classification task is genuinely nonlinear, the linear baseline drops to 83.3%, while SSP-KAN [784,20,10] achieves 86.2 ± 0.2%, maintaining the same 2.9 percentage points gain over the linear model (Fig. 6b). The consistent margin across both tasks confirms that MZI-VOA-SOA-VOA transfer functions provide benefit on tasks where nonlinear feature interactions are essential for classification.

The confusion matrices (Fig. 6c, d) reveal that SSP-KAN errors are concentrated among visually similar classes. On MNIST, the most common confusions occur between digits that share structural features (e.g., 4/9, 3/5, 7/9). On Fashion-MNIST, the dominant error mode is confusion among upper-body garments (shirt, coat, pullover, and T-shirt/top), which differ primarily in subtle textural features rather than gross shape. These error patterns are consistent with the limited expressivity of four-parameter optical transfer functions: the network captures coarse nonlinear features effectively but cannot resolve fine-grained distinctions that would require higher-order activation function complexity.

## Discussion

The central question addressed by this work is whether the constrained nonlinear transfer functions of standard telecom components can serve as trainable activations in a structured neural network. Each MZI-VOA-SOA-VOA module provides four tunable parameters—injection current *I*, attenuations *α*_1_ and *α*_2_, and phase *ϕ*—and the results confirm that this four-parameter family yields sufficient diversity to support nonlinear inference across classification, regression, and image recognition tasks. Specifically, SSP-KAN achieves 94.3% accuracy (±3.9% s.d.) on Two Moons classification ([2,2], 10 seeds), *R*^2^ = 0.986 ± 0.015 on yacht hydrodynamics regression ([6,1,1], 10 seeds), and 93.9 ± 0.2% on MNIST image classification (10 seeds). All results are obtained from a fully differentiable physics-based model grounded in Agrawal–Olsson SOA gain dynamics^23^ and commercially specified device parameters (Thorlabs BOA1554P), enabling end-to-end optimization via backpropagation.

The Two Moons and yacht hydrodynamics results illustrate how compositional depth compensates for limited per-module expressivity. Each MZI-VOA-SOA-VOA module implements a cosine-squared nonlinearity modulated by SOA gain saturation (Eq. (15)); this four-parameter family is substantially more constrained than B-spline activations with tens or hundreds of degrees of freedom. The single-layer [2,2] SSP-KAN achieves 94.3% (±3.9% s.d.) on Two Moons with four modules. The seed variance (best seed: 98.6%, worst: 88.1%) indicates multiple local minima in the loss landscape, a consequence of the periodic MZI transfer function. Adding a second layer ([2,2,2], 99.1% (±1.2% s.d.)) reduces both the accuracy gap and the seed sensitivity. On yacht hydrodynamics, the single-layer [6,1] network (*R*^2^ = 0.921 ± 0.017) is limited by its inability to capture feature interactions: each module processes one input independently. Adding a second layer ([6,1,1], *R*^2^ = 0.986 ± 0.015) enables the network to compose six univariate functions, exceeding the pyKAN baseline (*R*^2^ = 0.977) with fewer than half the parameters (28 vs 60). Cascading physically constrained nonlinearities produces richer functional representations than increasing parallel width alone. Multi-layer architectures with intermediate optical summation nodes introduce coherent phase accumulation effects, analyzed in the Supplementary Material.

Robustness analyses confirm resilience to the two dominant hardware impairments: finite DAC resolution and optical noise from amplified spontaneous emission. Across both tasks, input quantization has negligible impact: accuracy and *R*^2^ remain essentially unchanged from Float32 down to 6-bit resolution. Optical noise is the primary limiting factor. On Two Moons, the [2,2] architecture retains ~92% accuracy at 14 dB SNR; on yacht hydrodynamics, the [6,1,1] network maintains *R*^2^ ≈ 0.96 at the same SNR. Under extreme noise (6 dB), both tasks degrade gracefully, converging toward a noise floor set by intrinsic task difficulty rather than architectural capacity. Quantization effects are negligible while noise effects are measurable; for practical implementations, investment in low-noise optical amplification will yield greater returns than high-resolution digital-to-analog conversion.

Physics-based noise analysis confirms that even in a 7-layer cascade, the output SNR remains above 49 dB, more than 35 dB above the phenomenological threshold (Supplementary Note 4). Noise statistics remain near-Gaussian after cascaded nonlinear propagation (skewness < 0.3, excess kurtosis < 0.3). The phenomenological power-domain model at 14 dB SNR is therefore a conservative bound, approximately 10^2^× more pessimistic than the physical ASE noise floor at the CW operating point. All rate-dependent claims are confined to electrical bandwidths *B*_*e*_ ≲ 1 GHz, where the steady-state SOA model is self-consistent and the signal–ASE beat noise, which scales linearly with *B*_*e*_, remains bounded (Supplementary Note 4). Quantifying inference performance in the multi-GHz pattern-dependent regime requires a transient gain model and is left to future work.

On MNIST and Fashion-MNIST, SSP-KAN achieves 93.9 ± 0.2% and 86.2 ± 0.2% accuracy, respectively, compared with linear baselines of 91.0% and 83.3%. The +2.9 percentage point margin over the linear baseline is consistent across both tasks, confirming that the optical nonlinearity provides measurable benefit even on the near-linearly-separable MNIST. Compared with the dual ring-assisted MZI (D-RAMZI) architecture of Peng et al.^18^, which achieves 98% MNIST accuracy using microring-based nonlinearities with nine tunable parameters per edge and custom silicon photonic fabrication, SSP-KAN employs four parameters per edge using commercially available fiber-coupled components. The D-RAMZI approach offers richer per-edge nonlinearities through resonance-enhanced transmission and cascaded interference; the remaining accuracy gap therefore quantifies the cost of reduced activation richness and suggests that enhancing per-module expressivity represents the most direct route toward closing this gap while retaining deployment simplicity.

It is important to distinguish between architectures used to probe scaling behavior and those intended for near-term experimental demonstration. The MNIST-scale [784,20,10] network, comprising 15,880 discrete MZI-VOA-SOA-VOA modules, is not proposed as a practical system; assembling such a configuration from fiber-coupled components would be prohibitive in footprint, cost, and thermal stability. Image classification serves instead to characterize SSP-KAN performance trends as input dimensionality and network width increase, informing future integrated implementations. In contrast, the Two Moons and yacht hydrodynamics configurations require only 4–7 modules and are directly realizable on an optical bench using commercially available components.

The scaling studies give initial evidence on how network size relates to performance. On Two Moons, adding a second layer improves accuracy from 94.3% ([2,2], 4 modules) to 99.1% ([2,2,2], 8 modules) and reduces seed sensitivity. On yacht hydrodynamics, the gain from [6,1] to [6,1,1] is substantial (*R*^2^: 0.921 ± 0.017 to 0.986 ± 0.015), confirming that depth helps most when the task requires feature interactions that a single additive layer cannot capture (Supplementary Note 1). This limitation is structural rather than specific to the four-parameter family: no single-layer additive KAN can represent XOR, for any activation family, because its two-class discriminant reduces to a sum of univariate functions. A single-layer B-spline KAN scores at chance on XOR for the same reason, and depth restores performance in both cases (Supplementary Note 1). On MNIST, SSP-KAN [784,20,10] reaches 93.9 ± 0.2%, a gain of 2.9 percentage points over the linear baseline. The four-parameter activation family, rather than network capacity, is the limiting factor at scale. Improving SSP-KAN performance on complex tasks will therefore require enhancing per-module expressivity, through cascaded SOA stages, resonant enhancement, or increased parameter count per edge, rather than increasing module count.

The principal physical limits to scaling are fan-out loss, noise accumulation, and phase stability. At the input layer, each feature is split to *n*_out_ edges, so the optical power entering each module scales as *P*_in_/*n*_out_. For the [784,20,10] network, each first-layer module receives *P*_in_/20, reducing the signal level relative to the SOA’s ASE noise floor. At the output of each layer, incoherent summation on the photodetector aggregates contributions from *n*_in_ edges, partially recovering the total signal power, but ASE noise from each module accumulates alongside it. The robustness analysis (sections “Nonlinear classification on Two Moons” and “Regression performance on yacht hydrodynamics”) provides empirical bounds: performance degrades gracefully down to 14 dB SNR, suggesting that cascaded networks can tolerate substantial per-stage noise provided the cumulative SNR remains above this threshold. Phase stability scales linearly with module count, since each MZI requires independent stabilization. For the 4–7 module configurations that are the experimental targets of this work, the stabilization overhead is manageable with standard TEC modules and low-bandwidth feedback. Integration onto photonic integrated circuits may become relevant for architectures requiring hundreds of modules, but is beyond the scope of the present work.

The present study is based on numerical simulations employing calibrated device parameters. Experimental realization is facilitated by the use of components operating well within standard specifications: input powers of 1–80 mW, injection currents of 600–1700 mA, and attenuation ranges of 0–30 dB. Every component in the MZI-VOA-SOA-VOA module is commercially available as a fiber-pigtailed, connectorised device. A near-term prototype can therefore be assembled entirely from catalog components without custom fabrication or cleanroom access (component specifications and assembly details are given in Supplementary Note 5). Two primary practical challenges must be addressed for experimental realization: accurate characterization of SOA transfer functions under varying bias conditions, and maintaining interferometric phase stability across modules. The Agrawal–Olsson model requires only three measurable device parameters (*P*_sat_, ${G}_{\max }$, *I*_tr_), each extractable from a standard characterization sweep. Active phase stabilization of fiber-based MZIs is routine in both photonic integrated circuits^24,25^ and fiber-optic systems^26^. The SOA power-dependent phase shift operates at MHz–GHz rates, separated from the dither-based stabilization frequency (~10 kHz) by more than four decades. It therefore does not corrupt the feedback loop (Supplementary Note 5). The immediate experimental objective is the [2,2] Two Moons configuration, comprising four fiber-coupled modules initialized with parameters obtained from simulation. Successful demonstration would validate both the fidelity of the physics-based model and the end-to-end training framework before scaling to the seven-module yacht hydrodynamics configuration.

A minimal experimental implementation of the [2,2] Two Moons network requires four MZI-VOA-SOA-VOA modules, a tunable laser source, DAC-controlled attenuators for input encoding, and two photodetectors at the output nodes. The training and deployment strategy, including offline and on-site approaches, is described in “Methods”. The trained parameter values for all modules are reported in Supplementary Note 3.

Parameter mismatch between trained set-points and their hardware realizations perturbs the transfer function. A systematic device-variability study (Supplementary Note 7) evaluates this sensitivity across four device parameters (*P*_sat_, ${G}_{\max }$, *I*_tr_, *α*_*H*_) in 120 training and 6000 Monte Carlo evaluation configurations. Training with known or measured device parameters compensates mismatch on all axes: yacht [6,1,1] retains *R*^2^ ≥ 0.955 across all eight perturbation conditions. The parameter that most requires characterization is *α*_*H*_, because it enters the MZI phase through Δ*ϕ*_SOA_ = −*α*_*H*_*h*/2; a standard gain–phase measurement determines it per device, after which the learned interference condition is preserved.

The SSP-KAN architecture is particularly relevant to signal equalization in coherent optical communications, where inference latency and bandwidth increasingly challenge electronic digital signal processing at next-generation data rates^1^. The per-edge structure maps naturally to wavelength- or space-division multiplexed systems, enabling parallel channel-wise processing. Integration with reservoir computing could combine the temporal feature extraction of SOA-based dynamical systems with the structured nonlinear readout of SSP-KAN. More broadly, the approach of constructing differentiable forward models directly from component physics and optimizing physical parameters via backpropagation extends beyond SOA-based nonlinearities. Any photonic platform with analytically tractable transfer functions (including Kerr-based or Brillouin-based mechanisms) can in principle be incorporated into this physics-aware training paradigm.

We have demonstrated SSP-KAN, a photonic implementation of small-scale Kolmogorov–Arnold Networks in which each network edge is realized by a dedicated MZI–SOA–VOA module acting as a trainable nonlinear activation function. Using only four physically grounded parameters per module, the architecture achieves 94.3% accuracy (±3.9% s.d.) on nonlinear classification ([2,2], 10 seeds), 99.1% (±1.2% s.d.) with a two-layer [2,2,2] network, *R*^2^ = 0.986 ± 0.015 on six-input regression, and 93.9 ± 0.2% on 784-dimensional image classification (10 seeds). Compositional depth compensates for constrained per-module expressivity and reduces seed sensitivity: cascading physically limited nonlinearities yields richer functional representations than increasing parallel width alone. Robustness analyses confirm that optical noise, rather than DAC resolution, is the dominant hardware impairment, with performance remaining above 92% accuracy on Two Moons and *R*^2^ > 0.96 on yacht down to 14 dB SNR. Multi-layer architectures with intermediate optical summation introduce coherent phase accumulation effects analyzed in the Supplementary Material.

The choice between coherent and incoherent source configurations has a depth-dependent effect on both expressivity and trainability. For the single-layer [2,2] network, incoherent power summation (99.4% accuracy, 16 parameters; single seed, Supplementary Note 2) substantially outperforms coherent field summation (91.8%, 20 parameters), despite the latter providing additional phase degrees of freedom: the MZI phase *ϕ* simultaneously shapes the per-edge transfer function and controls the output interference condition, creating a multimodal loss landscape that impedes gradient-based training. For the two-layer [2,2,2] network, this balance reverses: coherent operation (99.9%, 40 parameters) slightly exceeds the incoherent baseline (99.5%, 32 parameters), as the interference cross-terms at intermediate nodes introduce input-dependent effective weights that enrich the network’s functional capacity. This depth-dependent crossover indicates that incoherent summation is preferable for shallow, experimentally accessible configurations, while coherent architectures may become advantageous as network depth increases and alternative optimization strategies are employed.

Clear limitations remain. The restricted four-parameter activation family produces a measurable accuracy gap relative to architectures employing resonant or highly engineered nonlinearities, such as the D-RAMZI design of Peng et al.^18^. This gap quantifies the cost of prioritizing experimental accessibility over per-edge expressivity, and indicates that enhancing activation richness (through cascaded stages, resonant enhancement, or alternative mechanisms such as stimulated Brillouin scattering or Kerr nonlinearity) represents the most direct route to closing it while retaining telecom compatibility. Furthermore, while the small-scale configurations studied here (4–7 modules) are directly realizable on an optical bench, scaling to high-dimensional tasks will require transition to integrated photonic platforms for compactness, thermal stability, and cost.

The fully differentiable physics model developed here provides a general framework for co-designing photonic neural network architectures with their physical substrates: any optical platform with analytically tractable transfer functions can in principle be incorporated into this optimization paradigm. The immediate experimental objective is the four-module [2,2] Two Moons configuration, initialized with simulation-trained parameters, to validate both model fidelity and the end-to-end training framework before scaling to the seven-module yacht hydrodynamics system. Beyond component-level validation, the per-edge SSP-KAN structure maps naturally to wavelength- or space-division multiplexed fiber systems, positioning it for latency-critical applications such as optical channel equalization where electronic digital signal processing increasingly struggles to keep pace with data rates.

## Methods

### Kolmogorov-Arnold networks

The Kolmogorov-Arnold representation theorem states that any multivariate continuous function $f:{[0,1]}^{n}\to {\mathbb{R}}$ can be exactly represented as a superposition of continuous univariate functions and addition:

2 $$f({x}_{1},\ldots,{x}_{n})={\sum}_{q=0}^{2n}{\Phi }_{q}\left({\sum}_{p=1}^{n}{\varphi }_{q,p}({x}_{p})\right)$$

where ${\varphi }_{q,p}:[0,1]\to {\mathbb{R}}$ and ${\Phi }_{q}:{\mathbb{R}}\to {\mathbb{R}}$ are continuous univariate functions^15^. This theorem implies that high-dimensional function approximation can be reduced to learning one-dimensional functions, fundamentally differing from the multilayer perceptron (MLP) approach where fixed nonlinearities are applied at nodes.

KANs use this representation by placing learnable activation functions on network edges rather than fixed activations at nodes. In a standard MLP, the transformation between layers takes the form **y** = *σ*(**Wx** + **b**), where *σ* is a fixed nonlinearity (e.g., ReLU or sigmoid) applied element-wise after a linear transformation. In contrast, a KAN layer computes:

3 $${y}_{j}={\sum}_{i=1}^{{n}_{{{{\rm{in}}}}}}{\varphi }_{i,j}({x}_{i})$$

This architectural difference has significant implications for photonic implementation. In conventional photonic neural networks, the linear matrix-vector multiplication **Wx** is naturally implemented using interference in Mach-Zehnder interferometer meshes or wavelength-division multiplexing, but the nonlinear activation *σ*(⋅) typically requires optical-to-electrical conversion, electronic processing, and electrical-to-optical conversion^10^. This OEO bottleneck limits throughput and increases power consumption.

The KAN architecture inverts this challenge: rather than requiring a single powerful nonlinearity after linear mixing, KANs require many weaker nonlinearities applied independently to each signal before linear summation. Optical summation (combining beams on a photodetector or through interference) is straightforward, while tunable optical nonlinearities, though constrained in functional form, are achievable through various physical mechanisms including saturable absorption, gain saturation, and nonlinear phase shifts^27^.

In software implementations, the univariate functions *φ*_*i*,*j*_ are typically parameterized as B-splines with learnable control points, providing smooth, flexible function approximation with *G* + *k* parameters per edge, where *G* is the grid size and *k* is the spline order^15^. A key question for photonic KANs is whether the more constrained transfer functions available from optical components, with only a handful of tunable parameters, can provide sufficient expressivity for practical tasks.

### MZI-VOA-SOA-VOA optical module

Our photonic KAN implementation uses a composite optical module consisting of a Mach-Zehnder interferometer (MZI) with a semiconductor optical amplifier (SOA) and variable optical attenuators (VOAs) in one arm. This configuration provides a tunable nonlinear transfer function with four trainable parameters: the SOA injection current *I*, two VOA attenuation coefficients *α*_1_ and *α*_2_, and the interferometer phase *ϕ*.

### Module architecture

The MZI splits the input optical power *P*_0_ equally between two arms via a 50/50 coupler (Fig. 1). The lower arm contains VOA_1_, followed by the SOA, followed by VOA_2_. The upper arm provides a reference path with adjustable phase *ϕ*. The signals recombine at a second 50/50 coupler, producing interference that shapes the output.

The power entering the SOA is:

4 $${P}_{{{{\rm{SOA,in}}}}}={\alpha }_{1}\cdot \frac{{P}_{0}}{2}$$

where ${\alpha }_{1}=1{0}^{-{A}_{1}/10}$ is the linear transmission corresponding to VOA_1_ attenuation *A*_1_ in dB.

Each component serves a distinct purpose: the injection current *I* controls the SOA small-signal gain *h*_0_; VOA_1_ sets the operating point on the gain saturation curve by controlling the power entering the SOA; VOA_2_ scales the amplitude of the SOA arm output independently of the saturation dynamics; and the phase *ϕ* determines the interference condition between the two arms.

### SOA gain dynamics

We model the SOA using the Agrawal–Olsson formalism, where the integrated gain $h=\int_{0}^{L}g(z)\,dz$ evolves according to the rate equation:

5 $$\frac{dh}{dt}=\frac{{h}_{0}-h}{{\tau }_{c}}-\frac{{P}_{{{{\rm{in}}}}}}{{E}_{{{{\rm{sat}}}}}}({e}^{h}-1)$$

where *h*_0_ is the small-signal integrated gain, *τ*_*c*_ is the carrier lifetime, and *E*_sat_ is the saturation energy. At steady state (*dh*/*dt* = 0), this becomes:

6 $$h={h}_{0}-\frac{{P}_{{{{\rm{in}}}}}}{{P}_{{{{\rm{sat}}}}}}({e}^{h}-1)$$

where *P*_sat_ = *E*_sat_/*τ*_*c*_ is the saturation power. This transcendental equation is solved numerically by Newton–Raphson iteration:

7 $${h}_{n+1}={h}_{n}-\frac{{h}_{n}-{h}_{0}+({P}_{{{{\rm{in}}}}}/{P}_{{{{\rm{sat}}}}})({e}^{{h}_{n}}-1)}{1+({P}_{{{{\rm{in}}}}}/{P}_{{{{\rm{sat}}}}})\,{e}^{{h}_{n}}}$$

initialized at *h*_0_, with three iterations sufficient for convergence across the operating range. The power gain is *G* = *e*^*h*^.

The small-signal gain *h*_0_ depends on the injection current *I*. Above the transparency current *I*_tr_, we use a calibrated relationship:

8 $${h}_{0}(I)=\kappa \left(\frac{I}{{I}_{{{{\rm{tr}}}}}}-1\right)$$

where ${h}_{0,\max }=\ln {G}_{\max }$ and the gain coefficient $\kappa={h}_{0,\max }/({I}_{\max }/{I}_{{{{\rm{tr}}}}}-1)$ is determined from device specifications. For our simulations, we use parameters corresponding to a commercially available SOA (Thorlabs BOA1554P): saturation power *P*_sat_ = 18 dBm, maximum small-signal gain ${G}_{\max }=35$ dB at maximum current ${I}_{\max }=1700$ mA, transparency current *I*_tr_ = 600 mA, and linewidth enhancement factor *α*_*H*_ = 5.

A critical feature of SOAs is the coupling between gain and phase through the linewidth enhancement factor. The SOA transforms the complex field envelope as:

9 $${E}_{{{{\rm{out}}}}}={E}_{{{{\rm{in}}}}}\,\exp \left(\frac{1-i{\alpha }_{H}}{2}\,h\right)$$

encapsulating both amplitude gain (*G* = *e*^*h*^) and a nonlinear phase shift:

10 $$\Delta {\phi }_{{{{\rm{SOA}}}}}=-\frac{{\alpha }_{H}}{2}\,h$$

This gain–phase coupling means that intensity-dependent gain saturation simultaneously produces intensity-dependent phase modulation, enriching the nonlinear transfer function beyond what either effect alone could provide.

### Transfer function

Each 50/50 directional coupler is described by the unitary transfer matrix:

11 $${{{\bf{C}}}}=\frac{1}{\sqrt{2}}\left(\begin{array}{rc}1&i\\ i&1\end{array}\right)$$

For input field ${E}_{0}=\sqrt{{P}_{0}}$ entering port 1, the coupler directs ${E}_{0}/\sqrt{2}$ to the SOA arm and $i{E}_{0}/\sqrt{2}$ to the reference arm. After propagation through the VOA_1_-SOA-VOA_2_ cascade (Eqs. (4)–(9)) and the reference-arm phase shifter, the two arm fields are:

12 $${E}_{1}^{\,{\mbox{out}}\,}=\frac{\sqrt{{\alpha }_{1}{\alpha }_{2}G}}{\sqrt{2}}\,{e}^{-i{\alpha }_{H}h/2}\,{E}_{0},$$

13 $${E}_{2}^{\,{\mbox{out}}\,}=\frac{i}{\sqrt{2}}\,{e}^{i\phi }\,{E}_{0}.$$

Applying the output coupler (Eq. (11)) and taking the transmitted port gives the output field:

14 $${E}_{{{{\rm{out}}}}}=\frac{{E}_{0}}{2}\left[\sqrt{{\alpha }_{1}{\alpha }_{2}G}\,{e}^{-i{\alpha }_{H}h/2}-{e}^{i\phi }\right]$$

The detected output power *P*_out_ = ∣*E*_out_∣^2^ yields the complete module transfer function:

15 $${P}_{{{{\rm{out}}}}}=\frac{{P}_{0}}{4}\left[{\alpha }_{1}{\alpha }_{2}G+1-2\sqrt{{\alpha }_{1}{\alpha }_{2}G}\cos \left(\frac{{\alpha }_{H}h}{2}+\phi \right)\right]$$

where *G* = *e*^*h*^ and *h* = *h*(*P*_SOA,in_, *h*_0_) is the solution to Eq. (6). The transfer function has the form of a Mach–Zehnder interference fringe whose visibility, fringe position, and input-dependent curvature are all controlled by the four trainable parameters. The key insight is that *α*_1_ affects not only the amplitude scaling but also the degree of gain saturation through Eq. (4), coupling the interference condition to the SOA operating point.

### Role of the two VOAs

**Fig. 7:**
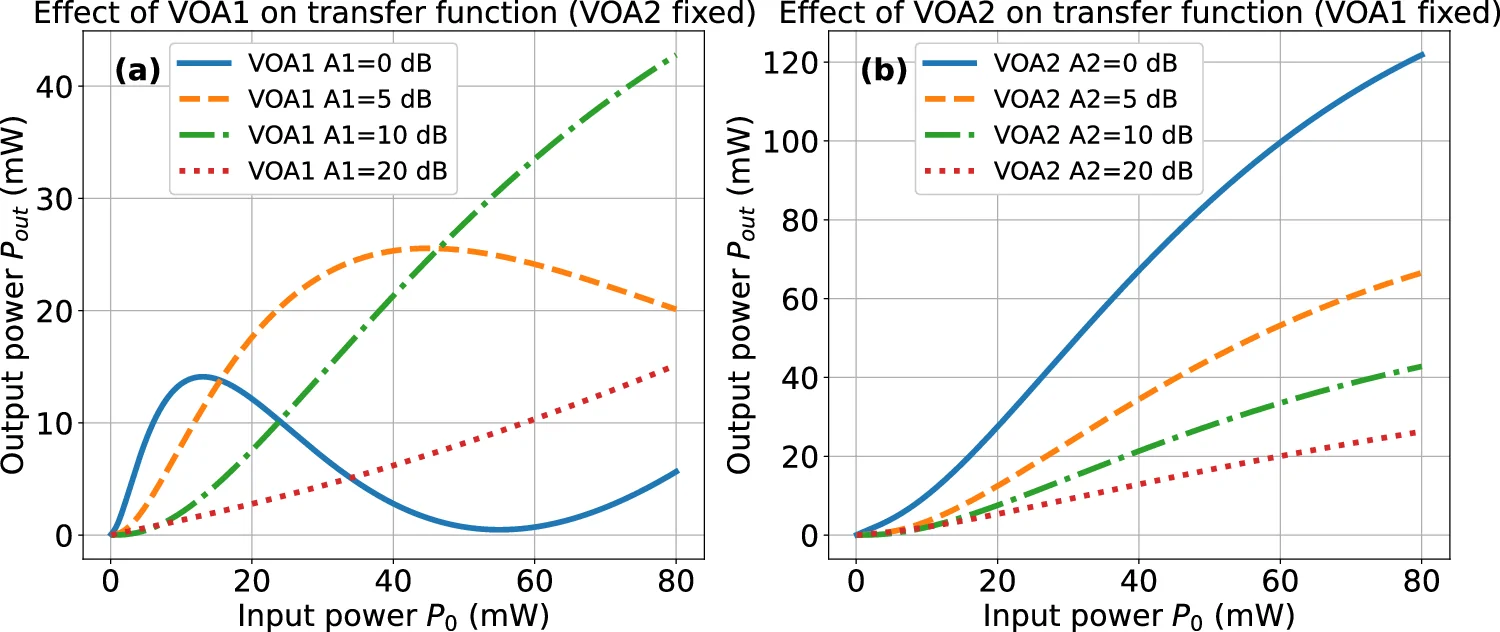
Distinct roles of the two VOAs in the MZI-VOA-SOA-VOA module transfer function. **a** Sweeping VOA_1_ changes the SOA operating point and saturation strength, producing pronounced changes in the nonlinearity shape. **b** Sweeping VOA_2_ primarily rescales the SOA-arm contribution at recombination, mainly adjusting interference contrast and overall scaling. Fixed parameters: *I* = 1200 mA, *ϕ* = *π*/2, *P*_sat_ = 18 dBm, *α*_*H*_ = 5, with *h*_0_(*I*) calibrated from ${G}_{\max }=35$ dB at ${I}_{\max }=1700$ mA and ${I}_{{{{\rm{tr}}}}}=600$ mA. In (**a**) VOA_2_ is fixed at *A*_2_ = 10 dB; in (**b**) VOA_1_ is fixed at *A*_1_ = 10 dB.

The two attenuators provide qualitatively different control knobs over the MZI-VOA-SOA-VOA module. VOA_1_ (pre-SOA) sets the SOA operating point via *P*_SOA,in_ = *α*_1_*P*_0_/2, and therefore tunes the degree of gain saturation and the associated gain-induced phase shift, changing the shape of the nonlinear transfer function. In contrast, VOA_2_ (post-SOA) does not alter the saturation dynamics because it is placed after the SOA; instead, it scales the relative amplitude of the SOA arm at recombination and thus mainly controls the interference contrast and output scaling. Figure 7 illustrates these distinct effects by sweeping VOA_1_ at fixed VOA_2_ and vice versa, for a representative operating point (*I* = 1200 mA, *ϕ* = *π*/2, *P*_sat_ = 18 dBm, *α*_*H*_ = 5, with *h*_0_(*I*) calibrated from ${G}_{\max }=35$ dB at ${I}_{\max }=1700$ mA and ${I}_{{{{\rm{tr}}}}}=600$ mA).

### Trainable parameters

Each MZI-VOA-SOA-VOA module provides four independently trainable parameters. The injection current *I* (600–1700 mA) controls the small-signal gain *h*_0_ through Eq. (8), with higher current producing stronger gain and more pronounced saturation nonlinearity. The input attenuation *α*_1_ (0–30 dB) sets the operating point on the saturation curve via Eq. (4): high attenuation keeps the SOA in its linear regime, while low attenuation drives it into saturation. The output attenuation *α*_2_ (0–30 dB) scales the SOA arm contribution to the interference without affecting the saturation dynamics. Finally, the phase *ϕ* (0–2*π*) determines whether the interference is constructive, destructive, or intermediate.

The entire forward pass—from Eq. (4) through the Newton–Raphson solution of Eq. (6) to the transfer function Eq. (15)—is implemented in PyTorch with automatic differentiation. Gradients with respect to all four parameters are computed via backpropagation through the Newton iteration, enabling end-to-end training of networks composed of multiple MZI-VOA-SOA-VOA modules.

### Noise and quantization models

Practical deployment of photonic neural networks requires robustness to hardware impairments. We model two primary sources of signal degradation: optical noise from amplified spontaneous emission (ASE) in the SOA, and quantization noise from finite-resolution digital-to-analog converters (DACs) used to encode input signals as optical power levels.

#### Optical noise models

We model optical noise as additive Gaussian perturbation in the power domain:

16 $${P}_{{{{\rm{noisy}}}}}={P}_{{{{\rm{in}}}}}+\sigma \cdot n$$

where $n \sim {{{\mathcal{N}}}}(0,1)$ and the result is clamped to non-negative values. The noise standard deviation is

17 $$\sigma=\sqrt{{P}_{\max }\cdot 1{0}^{-{{\mbox{SNR}}}_{{{{\rm{dB}}}}}/10}}$$

where ${P}_{\max }$ is the maximum operating power. This phenomenological model is deliberately more pessimistic than physical ASE noise: at the operating point (*P*_in_ = 10–80 mW, *G* ≈ 15–30), the inverse optical signal-to-noise ratio (OSNR) from amplified spontaneous emission is approximately 0.1–0.3%, two orders of magnitude below the 14 dB SNR threshold (~4% relative noise) used in the robustness analysis. The power-domain model therefore implicitly covers ASE, laser relative intensity noise (RIN), coupling fluctuations, thermal drift, and detector noise. A full physics-based derivation and four validation experiments are given in Supplementary Note 4.

#### Input quantization

The input to each optical module must be encoded as an optical power level, typically using a laser source modulated by a DAC-controlled attenuator or modulator. Finite DAC resolution introduces quantization error. For a *b*-bit DAC spanning the power range $[{P}_{{{{\rm{min}}}}},{P}_{\max }]$, the quantized input is obtained by rounding to the nearest discrete level:

18 $${P}_{{{{\rm{quant}}}}}={{\mbox{round}}}\,\left(\frac{{P}_{{{{\rm{in}}}}}-{P}_{{{{\rm{min}}}}}}{{P}_{\max }-{P}_{{{{\rm{min}}}}}}\cdot ({2}^{b}-1)\right)\cdot \frac{{P}_{\max }-{P}_{{{{\rm{min}}}}}}{{2}^{b}-1}+{P}_{{{{\rm{min}}}}}$$

where 2^*b*^ − 1 is the number of discrete levels available (for example, 255 for an 8-bit converter). To enable gradient-based training despite the non-differentiable rounding operation, we employ the straight-through estimator (STE): the forward pass uses quantized values while the backward pass computes gradients as if no quantization occurred.

We evaluate DAC resolutions from 4 to 16 bits. An 8-bit DAC provides 256 levels over the operating range, corresponding to a step size of approximately 0.3 mW for a 10–80 mW range. Our results in section “Results” demonstrate that SSP-KAN maintains high accuracy down to 6-bit resolution, with gradual degradation at lower bit depths.

### Training procedure

All SSP-KAN models are trained end-to-end via backpropagation, with optical transfer-function gradients computed by automatic differentiation through the MZI-VOA-SOA-VOA forward model described above. Because no physical hardware is in the loop during training, the procedure reduces to standard gradient-based optimization of the four trainable parameters (*I*, *α*_1_, *α*_2_, *φ*) per module, with the physics encoded entirely in the differentiable forward pass. All trainable parameters are constrained to the physical operating ranges specified by the device datasheets: injection current *I* ∈ [600, 1700] mA, attenuations *α*_1_, *α*_2_ ∈ [0, 30] dB, and phase *ϕ* ∈ [0, 2*π*]. Each parameter is reparameterised as $\theta={\theta }_{\min }+\sigma (z)({\theta }_{\max }-{\theta }_{\min })$, where *z* is the unconstrained optimizer variable and *σ* is the logistic sigmoid. This smooth bijection ensures that the gradient landscape is smooth and bounded regardless of the raw parameter scale, preventing initialization-dependent convergence failures. Multi-seed evaluation showed that alternative parameterizations (e.g., softplus with post-hoc clamping) left the raw parameter scale uncontrolled, causing certain seeds to converge to narrow minima sensitive to device variation (Supplementary Note 7). The sigmoid reparameterisation eliminates this failure mode. When the differentiable model is unavailable or insufficiently accurate, backpropagation-free training is also feasible: gradient-free optimizers (CMA-ES, PEPG, SPSA) can train SSP-KAN directly from measured network outputs at a cost of a few thousand forward-pass evaluations (Supplementary Note 6). The trained parameter values for every module are reported alongside each result (see Supplementary Information); these values map directly onto experimental set-points.

#### Datasets and preprocessing

We evaluate SSP-KAN on four benchmarks spanning binary classification, multivariate regression, and high-dimensional image classification (Table 1).

**Table 1:** Benchmark datasets and preprocessing details

Dataset	Samples	Input dim.	Task	Split	Power range (mW)
Two Moons	2000	2	Classification	50/50	10–80
Yacht Hydro.	308	6	Regression	70/15/15	10–80
MNIST	70,000	784	Classification	72/8/20	1–10
Fashion-MNIST	70,000	784	Classification	72/8/20	1–10

For all datasets, inputs are linearly scaled to optical power: $P={P}_{\min }+x\cdot ({P}_{\max }-{P}_{\min })$, where *x* ∈ [0, 1] is the normalized input. Two Moons and yacht hydrodynamics use the range 10–80 mW, representative of typical fiber-compatible operating powers. For MNIST and Fashion-MNIST, a power-range sweep identified 1–10 mW as optimal, where the MZI-VOA-SOA-VOA transfer functions operate in a more linear regime better suited to the high-dimensional, low-contrast nature of pixel inputs. The Two Moons dataset comprises 1000 training and 1000 test samples with Gaussian noise (*σ* = 0.1), generated using scikit-learn’s make_moons. The yacht hydrodynamics dataset^21,22^ contains 308 samples split 70/15/15 into training, validation, and test sets. MNIST and Fashion-MNIST use the full 70,000-sample datasets with stratified 72/8/20 splits, ensuring balanced class representation in each partition.

#### Optimization

All models are trained using the AdamW optimizer^28^. For Two Moons and yacht hydrodynamics, which involve small datasets, we use full-batch training for 5000 steps (Two Moons) or 5000 epochs (yacht), with a OneCycleLR schedule (30% warmup, cosine annealing), weight decay 10^−5^, and learning rates of 5 × 10^−3^ (Two Moons) and 10^−2^ (yacht SSP-KAN) or 5 × 10^−3^ (yacht baselines). For the yacht task, early stopping with a patience of 500 epochs monitors validation loss to prevent overfitting on the small dataset, and the best-validation-loss checkpoint is restored after training. For MNIST and Fashion-MNIST, we train for 150 epochs with batch size 256, learning rate 10^−2^, weight decay 10^−3^, and a 5-epoch linear warmup followed by cosine annealing. The best model by validation accuracy is selected. Classification tasks use cross-entropy loss; the regression task uses mean squared error. No data augmentation, label smoothing, or stochastic weight averaging is applied in any experiment.

#### Baseline models

To isolate the contribution of optical nonlinearity, we compare against several baselines trained under identical optimization conditions. A linear model with the same input-output dimensions measures the performance achievable without any nonlinear activation. For Two Moons and yacht hydrodynamics, we additionally compare against software KAN (pyKAN^15^) with B-spline grid sizes *G* = 1 and *G* = 10, representing low- and high-capacity software KAN baselines, and a multi-layer perceptron (MLP) with one hidden layer and ReLU activations, sized to approximately match the SSP-KAN parameter count. For image classification, we compare across SSP-KAN architectures of varying depth and width to characterize the effect of network topology.

#### Hardware robustness evaluation

To assess the effect of realistic hardware impairments, we retrain each model from scratch under every combination of input quantization level and optical noise condition, using the same optimizer and schedule as the no-noise baseline. Input quantization is swept from Float32 to 4-bit DAC resolution (16 levels). Optical noise is applied in the power domain (Eq. (16)) at SNR levels from no noise to 6 dB. This train-under-impairment protocol tests whether the SSP-KAN architecture can learn effective representations when noise and quantization are present throughout training, representing the expected operating condition for physical hardware where impairments are always active.

### Training and deployment strategies

The results in this work use offline training: the four per-module parameters are optimized via backpropagation through the differentiable physics model on a standard digital computer. The trained set-points are then transferred to hardware by programming current sources, VOA drivers, and phase controllers directly. This approach assumes that the device-level constants (*P*_sat_, ${G}_{\max }$, *I*_tr_) are known from prior characterization. The primary practical requirement is accurate characterization of each SOA module. A standard *P*_out_ versus *P*_in_ sweep at several bias currents suffices to extract the three Agrawal–Olsson parameters. Residual model–device mismatch can be compensated by a fine-tuning pass that re-optimizes the four per-module trainable parameters while holding the device constants fixed.

When the physical channel is unknown or the differentiable model is insufficiently accurate, on-site training without backpropagation becomes necessary. Three gradient-free optimizers are suitable for this setting. Covariance Matrix Adaptation Evolution Strategy (CMA-ES)^29^ maintains a multivariate Gaussian search distribution over the parameter space and adapts both its mean and covariance at each generation. It offers robust optimization over multimodal landscapes without gradient information. Parameter-Exploring Policy Gradients (PEPG) estimates the policy gradient by evaluating symmetric perturbations around the current parameter vector, offering lower variance than standard finite-difference methods. Simultaneous Perturbation Stochastic Approximation (SPSA) approximates the gradient using only two function evaluations per iteration regardless of parameter dimensionality. All three operate by evaluating the physical network output directly; no optical backpropagation or adjoint computation is required. Supplementary Note 6 presents CMA-ES results on the Two Moons and yacht hydrodynamics tasks.

Thermal drift of the MZI phase is a shared concern for both training strategies. In offline training, drift between characterization and deployment degrades the fidelity of the transferred set-points. In on-site training, drift during the optimisation process changes the objective landscape between evaluations. For the small-scale configurations studied here (4–7 modules), standard thermo-electric cooler stabilization and periodic recalibration are expected to be sufficient. Quantitative stability requirements are discussed in Supplementary Note 5.

### Energy analysis

The dominant electrical draw in an SSP-KAN module is the SOA bias current. The BOA1554P datasheet specifies a forward voltage of 1.5 V (typical) to 2.2 V (maximum). At a typical trained operating point (*I* ≈ 1200 mA, *V* ≈ 1.5 V), each SOA dissipates *P*_elec_ = *I* × *V* ≈ 1.8 W. VOA and phase-shifter biasing are two orders of magnitude below this and are neglected. The [2,2] Two Moons configuration draws 4 × 1.8 = 7.2 W across four SOAs; the [6,1,1] yacht configuration draws 7 × 1.8 = 12.6 W across seven. At a 1 GHz readout rate, this gives 7.2 nJ and 12.6 nJ per inference. This estimate accounts only for SOA electrical bias; DAC drivers, photodetector electronics, and feedback control are excluded.

For comparison, the equivalent electronic MLP for the yacht task has architecture [6 → 4 → 1] with 33 parameters. Its arithmetic cost is 6 × 4 + 4 × 1 = 28 multiply-accumulate (MAC) operations per inference. On a 45 nm CMOS process, a single 8-bit MAC costs approximately 1–5 pJ depending on whether operands are served from registers or SRAM^30,31^. The resulting 28–140 pJ per inference is a bare-datapath estimate for an idealized ASIC; it excludes clock distribution, control logic, I/O, and data movement beyond the immediate memory hierarchy. System-level energy for a real electronic implementation is higher. SSP-KAN at 7.2 nJ is approximately 50–250× less energy-efficient than this bare electronic estimate.

The BOA1554P is a fiber-optic booster amplifier designed for high output power in long-haul transmission, with 35 dB gain and 18 dBm saturation power. SSP-KAN does not require these specifications. The architecture requires only sufficient gain saturation to produce a tunable nonlinear transfer function through Eq. (15). A low-power SOA with ${G}_{\max } \sim 10$–15 dB and lower bias current would draw approximately 50 mW per device. For [2,2] with four such SOAs, the total draw is ~200 mW, giving ~200 pJ per inference at 1 GHz. This is comparable to the bare electronic MAC estimate (28–140 pJ). The BOA1554P therefore sets an upper bound on per-inference energy; the actual cost scales with device selection.

SSP-KAN does not target raw energy efficiency over electronic ASICs at these scales. The architectural advantages are single-pass latency at group-velocity speed (sub-nanosecond through the module), native optical-domain operation without OEO conversion, and analog parallelism through wavelength- or space-division multiplexing. A rigorous system-level energy comparison accounting for all overheads on both the optical and electronic sides remains an open benchmarking problem. Device selection and WDM parallelism are the primary levers for reducing per-inference energy.

## Supplementary information

Supplementary Information

Description of Additional Supplementary Files

Supplementary Movie 1

Supplementary Movie 2

Supplementary Movie 3

Transparent Peer Review file

## Source data

Source Data

## Data Availability

The Two Moons dataset is generated synthetically using sklearn.datasets.make_moons. The UCI Yacht Hydrodynamics dataset is publicly available^21^. MNIST and Fashion-MNIST are standard benchmarks available through PyTorch (torchvision.datasets). Source data are provided with this paper.

## Code availability

Source code for SSP-KAN, including the differentiable MZI-VOA-SOA-VOA model and all experiment scripts, is archived in a Code Ocean capsule (10.24433/CO.0732940.v1) and is available at https://github.com/StoleMyIceCream/ssp-kan, which will be made public upon publication.
