# Supplementary material for: Small-scale photonic Kolmogorov-Arnold networks using standard telecom nonlinear modules
Source: Nat Commun. 2026 Jul 21;17:8938. doi: 10.1038/s41467-026-75602-8 (PMC13503846; doi:10.1038/s41467-026-75602-8)
Supplement: Supplementary file 1 [file 41467_2026_75602_MOESM1_ESM.pdf]

Small-scale photonic Kolmogorov-Arnold networks using standard telecom nonlinear modules

Supplementary Note 1: Architectural limitations of single-layer SSP-KAN

A single-layer [2,2] SSP-KAN computes each output as a sum of univariate functions, $y_j = \varphi_{1,j}(x_1) + \varphi_{2,j}(x_2)$. This additive structure cannot represent decision boundaries that depend on interactions between inputs. Two tasks illustrate the limitation (Supplementary Fig. 1).

The XOR task assigns opposite labels to diagonally opposed quadrants, requiring a boundary that depends jointly on both features. No single-layer additive KAN can represent it, for any activation family: the [2,2] discriminant is a sum of univariate functions, $y_1 - y_2 = a(x_1) + b(x_2)$, and a sign threshold t would require

$$a(+)+b(+) > t, \quad a(-)+b(-) > t, \quad a(+)+b(-) < t, \quad a(-)+b(+) < t, \tag{1}$$

which is impossible: the first two and the last two inequalities sum to the same total $a(+)+a(-)+b(+)+b(-)$, forcing it both above and below $2t$. The limitation is structural, not a property of the optical transfer function; a single-layer B-spline KAN fails identically. The [2,2] network achieves only 51.8% accuracy, indistinguishable from random chance. Adding a second layer recovers 89.4%: the [2,2,2] network composes first-layer outputs to approximate the diagonal structure, though the task remains challenging owing to the sharp axis-aligned boundaries that smooth optical transfer functions approximate imperfectly.

A subtler failure arises when the Two Moons dataset is rotated by 45°. In its standard orientation the crescent boundary is approximately separable along the coordinate axes, and the [2,2] network reaches 94.3% (±3.9% s.d., 10 seeds) (the main-text subsection on nonlinear classification of Two Moons). After rotation the boundary runs diagonally, breaking this separability: the [2,2] network drops to 89.0%, close to the linear baseline. The [2,2,2] network restores 99.9% by composing first-layer features to reconstruct the rotated separation. This pair of results, 94.3% at 0° versus 89.0% at 45°, quantifies the cost of the additive constraint and motivates multi-layer architectures, which in turn raise the coherent phase accumulation effects analyzed in Supplementary Note 2.

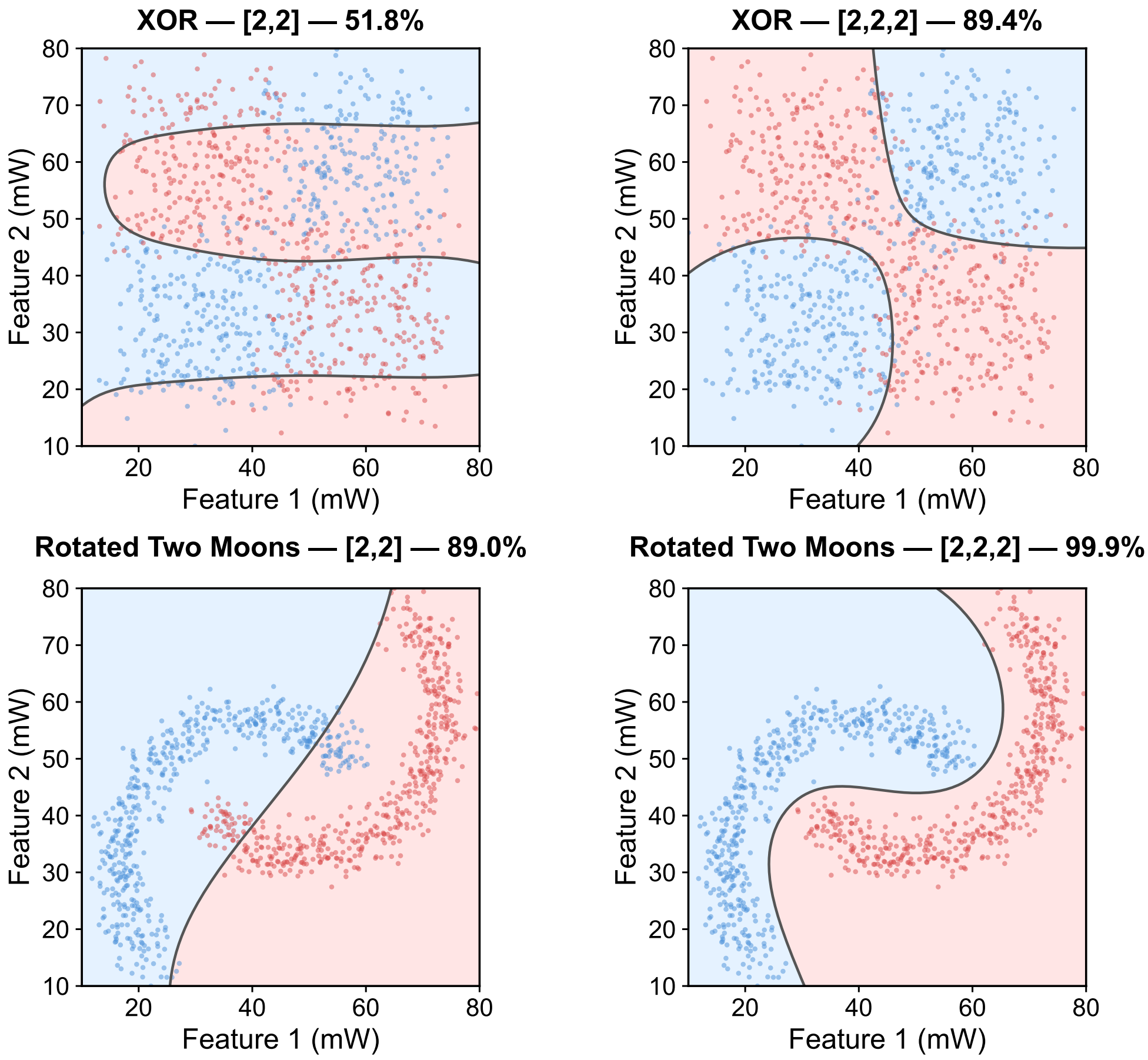

Supplementary Figure 1: Tasks requiring feature interaction. Left column: single-layer [2,2]; right column: two-layer [2,2,2]. Top row: XOR. No single-layer additive model can represent XOR (Eq. 1). The [2,2] boundary is at chance (51.8%) while [2,2,2] recovers 89.4%. Bottom row: Two Moons rotated 45°. The [2,2] network (89.0%) falls to the linear baseline because the additive structure cannot represent diagonal boundaries, while [2,2,2] reaches 99.9%.

Supplementary Note 2: Coherent versus incoherent operation

Throughout the main text, module outputs combine as optical powers, which is valid when the recombining fields are mutually incoherent. If instead all paths originate from a single laser, the fields retain a fixed phase relationship and combine coherently, producing interference terms that alter the network computation. This section derives the incoherent and coherent summation regimes and compares them on the Two Moons benchmark.

At each output or intermediate node j, the n_{in} modules routed to that node contribute fields that recombine through a coupler tree. In the most general form, the detected power is

$$y_j = \left| \frac{1}{\sqrt{n_{\text{in}}}} \sum_{i=1}^{n_{\text{in}}} c_{ij} \, E_{f_{ij},\text{out}} \right|^2, \tag{2}$$

where c_{ij} is the phase factor accumulated by the field traveling from input i to output j through the coupler network. Expanding the square produces n_{in} direct terms and $n_{\text{in}}(n_{\text{in}}-1)/2$ cross-terms, each depending on the relative phase between a pair of modules. The two regimes correspond to whether these cross-terms survive or vanish.

Supplementary Note 2.1: Incoherent regime

When the combining fields originate from separate lasers, or when path-length mismatch exceeds the coherence length, the cross-terms average to zero and only powers add. For a [2,2] layer the outputs reduce to

$$y_1 = \tfrac{1}{2}\left[f_1(x_1 P_0/4) + f_3(x_2 P_0/4)\right], \tag{3}$$

$$y_2 = \tfrac{1}{2}\left[f_2(x_1 P_0/4) + f_4(x_2 P_0/4)\right], \tag{4}$$

where f_k is the power-domain transfer function of module k and the factors 1/4 and 1/2 arise from successive 50/50 couplers. The effective weight on every edge is fixed at 1/2; only the four module parameters $(I, \alpha_1, \alpha_2, \phi)$ per edge are trainable. This is the regime assumed throughout the main text (16 trainable parameters for [2,2]).

Supplementary Note 2.2: Coherent regime

When all signals share the same source, the coupler phase factors c_{ij} in Eq. (2) must be tracked. Each lossless 50/50 coupler follows the transfer matrix:

$$\mathbf{C} = \frac{1}{\sqrt{2}} \begin{pmatrix} 1 & i \\ i & 1 \end{pmatrix}, \tag{5}$$

where the bar port transmits unchanged and the cross port acquires a factor i. A field traversing $n_\times$ cross ports through a cascade of couplers therefore accumulates a phase factor $i^{n_\times}$.

For a [2,2] layer fed by a coherent source $E_0 = \sqrt{P_0}$, inputs are encoded via amplitude modulators ($E \to \sqrt{x}\, E$) and each arm is split once more to address two modules. Each field traverses two couplers: module 1 takes two bar ports (phase factor $i^0 = +1$), modules 2 and 3 each take one cross port ($i^1 = +i$), and module 4 takes two cross ports ($i^2 = -1$). In our simulation we label each path by a binary index k encoding bar (0) or cross (1) at each stage, so that the phase factor reduces to $i^{\,\text{popcount}(k)}$.

The resulting input fields are

$$E_{f_1,\text{in}} = +\frac{\sqrt{x_1}\, E_0}{2}, \qquad E_{f_2,\text{in}} = +\frac{i\sqrt{x_1}\, E_0}{2}, \tag{6}$$

$$E_{f_3,\text{in}} = +\frac{i\sqrt{x_2}\,E_0}{2}, \qquad E_{f_4,\text{in}} = -\frac{\sqrt{x_2}\,E_0}{2}. \tag{7}$$

The sign pattern $(+1, +i, +i, -1)$ is fixed by coupler unitarity and enters every downstream interference term.

Each module acts on the complex field as

$$E_{f_k,\text{out}} = t_k\big(|E_{f_k,\text{in}}|^2\big)\, e^{\,i\theta_k(|E_{f_k,\text{in}}|^2)} \cdot E_{f_k,\text{in}}, \tag{8}$$

where t_k is the amplitude transmission and θ_k the total output phase, both set by the SOA gain dynamics and the module parameters. Expanding Eq. (2) for the [2,2] case (modules 1 and 3 feeding output 1) gives

$$y_1 = \frac{1}{2}\big[|E_1|^2 + |E_3|^2 + 2|E_1||E_3|\sin(\theta_1 - \theta_3)\big]. \tag{9}$$

The cross-term has two consequences. First, because θ_k depends on input power through SOA gain saturation, the effective weights at recombination nodes vary with the signal. Second, each module's MZI phase ϕ_k now controls both the transfer function shape and the interference condition, coupling two roles that are independent in the power-domain formulation.

A phase shifter ψ_k placed after each module separates these roles:

$$y_1 = \frac{1}{2}\big[|E_1|^2 + |E_3|^2 + 2|E_1||E_3|\sin(\theta_1 - \theta_3 + \Delta\psi_{13})\big], \tag{10}$$

where $\Delta\psi_{13} = \psi_1 - \psi_3$ controls the interference independently of the MZI-VOA-SOA-VOA parameters that shape the activation. This adds one parameter per module (5 per edge versus 4 in the incoherent regime).

Supplementary Note 2.3: Experimental comparison

Supplementary Fig. 2 compares both regimes on Two Moons classification for [2,2] and [2,2,2] architectures.

For the single-layer [2,2] network, the incoherent regime (99.4%, 16 parameters; single seed — multi-seed statistics in the main-text subsection on nonlinear classification of Two Moons) substantially outperforms the coherent regime (91.8%, 20 parameters). Although coherent operation provides the additional ψ degrees of freedom, it also couples ϕ_k to the interference condition at the output couplers. Since the MZI transfer function already depends on ϕ_k through a cosine-squared fringe, this coupling multiplies the number of local minima in the loss landscape: the same parameter now sits inside two periodic functions with different periods. Gradient descent is more likely to become trapped, and in this case settles on an inferior boundary. The performance gap reflects the difficulty of gradient-based optimization on the resulting multimodal landscape, not a limitation of the coherent model, which is strictly more expressive. Alternative training strategies which handle multimodal objectives more naturally may recover the missing performance.

For the two-layer [2,2,2] network, the situation reverses: the coherent regime (99.9%, 40 parameters) slightly exceeds the incoherent regime (99.5%, 32 parameters). Here the interference cross-terms at intermediate nodes act as input-dependent effective weights that the incoherent formulation cannot access. The additional ψ parameters allow the optimizer to exploit this extra expressivity without the ϕ-interference coupling penalty dominating.

The choice of source coherence therefore affects both expressivity and trainability, and the balance between them depends on network depth. For the small-scale configurations studied in the main text, the incoherent regime offers a simpler optimization landscape at no cost in performance for [2,2] and [6,1,1]. The coherent regime becomes advantageous only when intermediate recombination nodes are present and the network is deep enough to exploit the additional interference degrees of freedom.

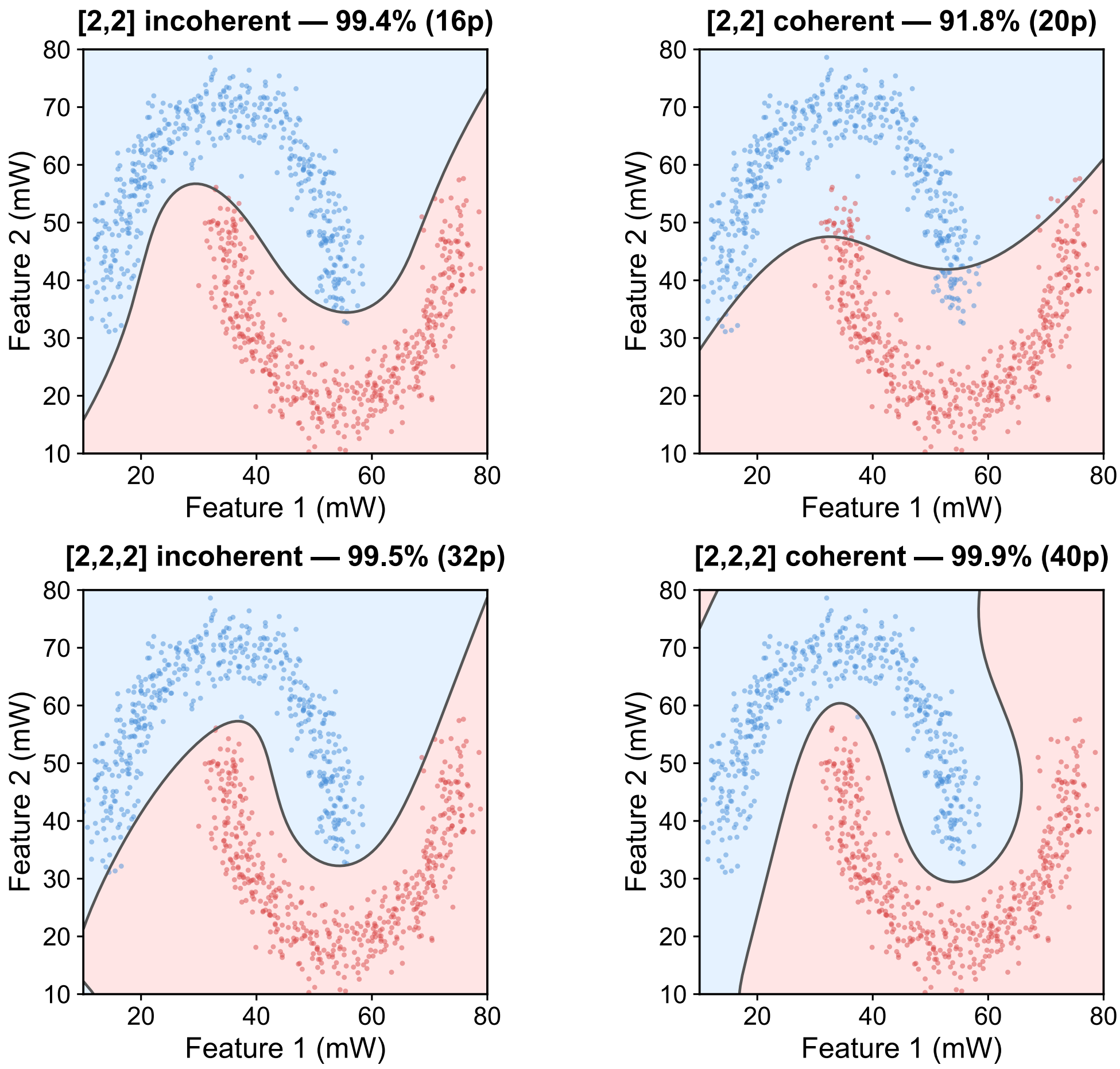

Supplementary Figure 2: Coherent versus incoherent operation on Two Moons classification (single seed). Top row: single-layer [2,2]. The incoherent regime (99.4%, 16 parameters) outperforms the coherent regime (91.8%, 20 parameters), indicating that interference at the output couplers complicates optimization without providing a net benefit at this depth. Bottom row: two-layer [2,2,2]. The coherent regime (99.9%, 40 parameters) slightly exceeds the incoherent regime (99.5%, 32 parameters), as the interference cross-terms at intermediate nodes introduce input-dependent effective weights that increase network expressivity. Multi-seed statistics for the incoherent regime are reported in the main text.

Supplementary Note 3: Trained module parameters

All trainable parameters are clamped to the physical operating ranges specified by the Thorlabs BOA1554P datasheet: injection current $I \in [600, 1700]$ mA, attenuations

$\alpha_1, \alpha_2 \in [0, 30]$ dB, and phase $\phi \in [0, 2\pi]$ rad. The values reported below are the effective parameters after the sigmoid reparameterisation applied during the forward pass, and correspond directly to the hardware set-points required for experimental realisation.

Supplementary Note 3.1: Two Moons classification

Supplementary Table 1: Trained parameters for the [2,2] SSP-KAN on Two Moons (4 modules, 16 parameters; mean test accuracy 94.3% (±3.9% s.d.) across 10 seeds; values shown are for a representative seed).

Module	I (mA)	α_1 (dB)	α_2 (dB)	ϕ (rad)
$\varphi_{0,0}$	1045.26	0.54	0.28	1.4926
$\varphi_{0,1}$	1363.19	0.70	3.69	3.2287
$\varphi_{1,0}$	1136.59	3.09	0.07	1.4761
$\varphi_{1,1}$	1219.03	0.28	7.91	1.8061

Supplementary Table 2: Trained parameters for the [2,2,2] SSP-KAN on Two Moons (8 modules, 32 parameters; mean test accuracy 99.1% (±1.2% s.d.) across 10 seeds; values shown are for a representative seed).

Layer	Module	I (mA)	α_1 (dB)	α_2 (dB)	ϕ (rad)
1	$\varphi_{0,0}$	1035.54	2.17	2.15	4.6215
1	$\varphi_{0,1}$	1358.99	0.02	0.22	3.3808
1	$\varphi_{1,0}$	1117.07	3.25	1.67	2.1629
1	$\varphi_{1,1}$	1222.74	6.14	4.19	4.6445
2	$\varphi_{0,0}$	931.23	2.53	0.25	4.5404
2	$\varphi_{0,1}$	951.79	1.97	4.62	2.5132
2	$\varphi_{1,0}$	833.05	0.96	0.11	5.1438
2	$\varphi_{1,1}$	908.22	1.81	0.12	1.3007

Supplementary Note 3.2: Yacht hydrodynamics regression

Supplementary Table 3: Trained parameters for the [6,1] SSP-KAN on yacht hydrodynamics (6 modules, 24 parameters; mean test $R^2 = 0.921 \pm 0.017$ across 10 seeds; values shown are for a representative seed).

Module	I (mA)	α_1 (dB)	α_2 (dB)	ϕ (rad)
$\varphi_{0,0}$	955.69	10.50	0.09	0.4920
$\varphi_{1,0}$	1169.22	15.78	1.13	3.2527
$\varphi_{2,0}$	950.79	10.64	0.11	0.7227
$\varphi_{3,0}$	1056.65	13.26	0.56	5.0791
$\varphi_{4,0}$	971.82	11.24	0.07	0.2775
$\varphi_{5,0}$	1308.35	3.07	7.73	3.2592

Supplementary Table 4: Trained parameters for the [6,1,1] SSP-KAN on yacht hydrodynamics (7 modules, 28 parameters; mean test $R^2 = 0.986 \pm 0.015$ across 10 seeds; values shown are for a representative seed).

Layer	Module	I (mA)	α_1 (dB)	α_2 (dB)	ϕ (rad)
1	$\varphi_{0,0}$	960.90	6.73	3.18	0.8875
1	$\varphi_{1,0}$	1176.99	7.65	7.22	4.7838
1	$\varphi_{2,0}$	956.79	3.84	8.26	2.2538
1	$\varphi_{3,0}$	1060.58	8.77	5.24	5.9785
1	$\varphi_{4,0}$	977.85	6.16	6.10	1.1401
1	$\varphi_{5,0}$	1300.58	2.62	2.18	3.6593
2	$\varphi_{0,0}$	930.61	0.36	0.05	2.9707

Supplementary Note 4: Physics-based noise model derivation and validation

This section derives physics-based noise models for the MZI-VOA-SOA-VOA module, validates them against the manufacturer-specified noise figure of the Thorlabs BOA1554P, and compares their predictions against the phenomenological noise models used in the main text.

Supplementary Note 4.1: Current-dependent ASE model

Supplementary Note 4.1.1: Spontaneous emission factor

The spontaneous emission factor n_{sp} quantifies the quality of population inversion in the gain medium. For a semiconductor optical amplifier with linear material gain $g = a(N - N_0)$, where a is the differential gain, N is the carrier density, and N_0 is the transparency carrier density [1]:

$$n_{\mathrm{sp}} = \frac{N}{N - N_0} \tag{11}$$

At high inversion ($N \gg N_0$), $n_{\mathrm{sp}} \to 1$. Near transparency ($N \to N_0^+$), $n_{\mathrm{sp}} \to \infty$. The practical range for InGaAsP SOAs is 1.4–4 [1].

Supplementary Note 4.1.2: Connection to the trainable parameter *I*

The carrier density obeys the rate equation [2]:

$$\frac{dN}{dt} = \frac{I}{qV} - R(N) - \Gamma v_g \, g(N) \, S \tag{12}$$

where I is the injection current, q is the electron charge, V is the active region volume, $R(N)$ is the total recombination rate, Γ is the optical confinement factor, v_g is the group velocity, and S is the photon density. In the unsaturated regime ($P_{\mathrm{in}} \ll P_{\mathrm{sat}}$), the stimulated emission term is negligible and $N \propto I$. At transparency, $N = N_0$ at $I = I_{\mathrm{tr}}$. Substituting into Eq. (11):

$$\boxed{n_{\mathrm{sp,unsat}} = \frac{I/I_{\mathrm{tr}}}{I/I_{\mathrm{tr}} - 1}} \tag{13}$$

At $I = 1200$ mA: $n_{\mathrm{sp,unsat}} = 2.0$. At $I = 1700$ mA: $n_{\mathrm{sp,unsat}} \approx 1.55$.

Supplementary Note 4.1.3: Saturation correction

At the SSP-KAN operating point ($P_{\mathrm{in}}/P_{\mathrm{sat}} \approx 0.16$–$1.27$), the stimulated emission term in Eq. (12) dominates. Carriers are depleted by the strong optical signal, clamping N below the unsaturated value and pushing it toward N_0. This increases n_{sp}.

Using the integrated gain $h = \Gamma a(N - N_0)L$, the saturated and unsaturated cases give:

$$N_{\mathrm{sat}} - N_0 = \frac{h}{h_0}(N_{\mathrm{unsat}} - N_0) \tag{14}$$

Substituting into $n_{\mathrm{sp}} = N_{\mathrm{sat}}/(N_{\mathrm{sat}} - N_0)$:

$$\boxed{n_{\mathrm{sp,sat}} = 1 + \frac{h_0}{h}\,(n_{\mathrm{sp,unsat}} - 1)} \tag{15}$$

At no saturation ($h = h_0$): $n_{\text{sp,sat}} = n_{\text{sp,unsat}}$. At deep saturation ($h \ll h_0$): n_{sp} grows as h_0/h, reflecting the degradation of inversion quality. Both h and h_0 are already computed in the forward pass (Newton–Raphson solver and Baveja model respectively), so this correction adds no new parameters.

Supplementary Note 4.1.4: ASE spectral density

The single-sided ASE power spectral density per polarisation mode at the SOA output is [3]:

$$S_{\text{ASE}} = n_{\text{sp}}(G-1)\,h\nu \quad \text{[W/Hz, per polarisation mode]} \tag{16}$$

where $G = e^h$ is the saturated power gain and $h\nu = 1.28 \times 10^{-19}$ J at 1550 nm.

Supplementary Note 4.1.5: ASE propagation through one MZI-VOA-SOA-VOA module

The MZI has two arms recombined at a 50:50 output coupler. ASE is broadband incoherent, so at the coupler it adds as power, not field. Tracing the ASE through each path:

Path	ASE contribution at output
Active arm (existing ASE)	$S_{\text{in}} \cdot \alpha_1\alpha_2 G/4$
Active arm (new ASE from SOA)	$\alpha_2 \cdot n_{\text{sp}}(G-1)h\nu/2$
Reference arm (existing ASE)	$S_{\text{in}}/4$

Summing:

$$S_{\text{out}} = \frac{1}{4}(\alpha_1\alpha_2 G + 1)\,S_{\text{in}} + \frac{1}{2}\,\alpha_2\,n_{\text{sp}}(G-1)\,h\nu \tag{17}$$

where S_{in} is the incoming ASE spectral density from the previous layer (zero for the first layer).

Supplementary Note 4.1.6: Multi-layer ASE accumulation

At each output node j in a KAN layer, ASE from multiple edges sums as power (broadband incoherent):

$$S_{\text{ASE},j} = \sum_i S_{\text{ASE,out},ij} \tag{18}$$

Signal power P_j and ASE spectral density $S_{\text{ASE},j}$ are tracked as two separate quantities through the network. In a multi-layer network (e.g. [2,2,2]), layer 2's SOAs receive layer 1's accumulated ASE as S_{in} in Eq. (17). The $\alpha_1\alpha_2 G$ factor in the first term represents re-amplification of upstream ASE by downstream SOAs. However, the total ASE power ($P_{\text{ASE}} = S_{\text{ASE}} \times B_o \approx 0.05\ \mu$W) remains five orders of magnitude below the signal power ($P_{\text{in}} = 10$–80 mW), so ASE does not saturate downstream SOAs.

Supplementary Note 4.1.7: Detection noise at the photodetector

Detection noise is computed once, at the final photodetector. Three noise terms arise from square-law detection [4, 5]:

Signal–ASE beat (dominant).

The coherent signal field and the broadband ASE field beat against each other at the photodetector:

$$\sigma^2_{\text{sig-sp}} = 4\, P_{\text{signal}}\, S_{\text{ASE}}\, B_e \quad [\text{W}^2] \tag{19}$$

where S_{ASE} is per polarisation mode. The factor 4 arises from the heterodyne geometry: 2 from mixing with ASE on both sides of the signal frequency, and 2 from both quadratures of the beat [5].

Shot noise (negligible at high gain).

$\sigma^2_{\text{shot}} = 2\, h\nu\, P_{\text{signal}}\, B_e$. The ratio $\sigma^2_{\text{shot}}/\sigma^2_{\text{sig-sp}} \sim 1/(2n_{\text{sp}}G)$, which is $\sim 10^{-3}$ at our operating point ($\sim$35 dB below signal–ASE beat).

ASE–ASE beat (negligible for strong signals).

$\sigma^2_{\text{sp-sp}} = 4\, S^2_{\text{ASE}}\, (2B_oB_e - B_e^2)$, where B_o is the optical filter bandwidth ($\sim$1–5 THz). This term dominates only when the signal is absent or very weak; for our regime ($P_{\text{signal}} \gg P_{\text{ASE}}$), it is negligible compared to signal–ASE beat.

Supplementary Note 4.1.8: CW validity bound

The gain equation used throughout this work sets $dh/dt = 0$, assuming steady-state carrier density. This requires the observation time Δt to be much longer than the carrier lifetime τ_c:

$$\Delta t \gg \tau_c \quad \Rightarrow \quad B_e \approx \frac{1}{\Delta t} \ll \frac{1}{\tau_c} \tag{20}$$

For the BOA1554P with $\tau_c \approx 200$–500 ps (typical bulk InGaAsP): $1/\tau_c \approx 2$–5 GHz. Self-consistency therefore requires $B_e \lesssim 1$ GHz. Since $\sigma^2_{\text{sig-sp}} \propto B_e$ (Eq. (19)), the noise is bounded to be small in exactly the regime where the CW model is valid.

Supplementary Note 4.2: Datasheet noise model

The BOA1554P datasheet specifies a fibre-to-fibre NF of 6–8 dB, corresponding to a linear noise figure:

$$F = 10^{\text{NF}_{\text{dB}}/10} \approx 4\text{–}6.3 \tag{21}$$

For a high-gain phase-insensitive amplifier, $F \approx 2\, n_{\text{sp}}$ [1], giving:

$$n_{\text{sp}} \approx F/2 \approx 2\text{–}3.15 \tag{22}$$

This measured NF already encodes all internal physics: spatially varying carrier density, internal waveguide loss, and fibre coupling loss. No approximation chain is needed. The same propagation equations (Eqs. (17)–(19)) apply, with n_{sp} fixed regardless of operating point.

The datasheet validates the unsaturated limit of the current-dependent model. At small signal ($h \to h_0$), Eq. (15) reduces to $n_{\text{sp,unsat}}$, and $F \approx 2\, n_{\text{sp,unsat}}$ matches the measured NF. The saturation correction itself (the h_0/h factor) has not been validated against input-power-dependent NF measurements for the BOA1554P.

Supplementary Note 4.3: Validation

To compare the four noise models under identical training conditions, we train SSP-KAN under nine noise configurations (baseline at 30 dB, power-domain at 14/20/30 dB, amplitude-domain at 14/20/30 dB, datasheet NF at 8 dB, and current-dependent) across three architectures with 10 random seeds each. Supplementary Table 5 reports the results side by side.

Supplementary Table 5: Noise model comparison across architectures and tasks (10 seeds, mean ± std). Both physics-based models produce performance statistically indistinguishable from the 30 dB baseline.

Noise model	Moons [2,2] (%)	Moons [2,2,2] (%)	Yacht [6,1,1] R^2
Baseline (30 dB)	95.2 ± 3.6	98.0 ± 3.3	0.977 ± 0.011
Power 14 dB	90.6 ± 2.5	91.5 ± 4.0	0.945 ± 0.014
Power 20 dB	91.5 ± 3.0	94.8 ± 3.4	0.967 ± 0.012
Power 30 dB	92.2 ± 3.4	97.3 ± 3.3	0.975 ± 0.012
Amplitude 14 dB	73.9 ± 0.3	64.0 ± 0.5	0.121 ± 0.173
Amplitude 20 dB	84.0 ± 0.5	75.9 ± 0.3	0.462 ± 0.127
Amplitude 30 dB	90.5 ± 2.6	86.8 ± 2.7	0.818 ± 0.144
Datasheet NF 8 dB	92.2 ± 3.4	97.8 ± 3.3	0.977 ± 0.011
Current-dependent	92.0 ± 3.4	97.9 ± 3.3	0.977 ± 0.011

The two physics-based models (datasheet NF and current-dependent) are indistinguishable from each other and from the baseline across all tasks, confirming that physical ASE noise at the CW operating point is negligible for inference. The amplitude-domain model is substantially worse at every SNR: at 14 dB, yacht R^2 drops to 0.121 ± 0.173, compared with 0.945 ± 0.014 for the power-domain model at the same nominal SNR. The difference arises because the amplitude model applies noise proportional to the signal field, so high-power signals receive disproportionately more noise. The power-domain model distributes noise uniformly regardless of signal level. Since the SSP-KAN operating range (10–80 mW) places the signal well above the noise floor, the amplitude model penalises strong signals far more than weak ones, collapsing the effective dynamic range.

A concern for SOA-based networks is whether the nonlinear transfer function distorts the noise statistics. If gain saturation introduced heavy tails or skewness, the Gaussian assumptions underlying the phenomenological models would break down at physical noise levels. To test this, we propagate 1000 Two Moons test samples through the trained [2,2,2] network and record the noise residuals (noisy output minus baseline output) at each layer (Supplementary Fig. 3). For both physics-based models, residuals after layer 1 remain near-Gaussian: skewness < 0.3 and excess kurtosis < 0.3 for all input classes. The phenomenological models at higher noise levels produce larger residuals but similar distributional shape. Non-Gaussian distortion from SOA saturation is negligible at physical noise levels.

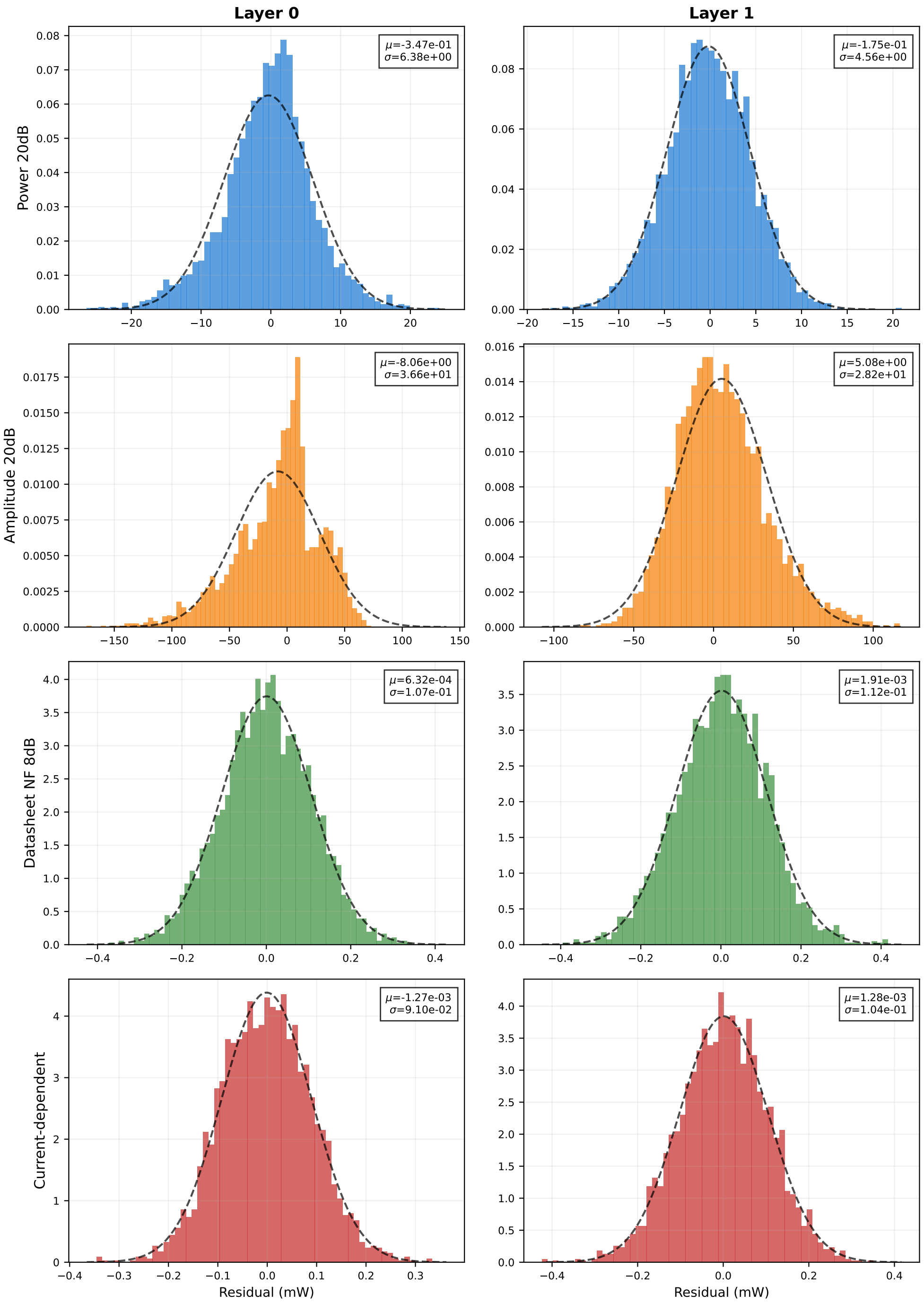

Supplementary Figure 3: Noise residual distributions (noisy output − baseline output) at the output of the [2,2,2] network for four noise models (rows) and two layers (columns), pooled across three representative inputs. The physics-based models (datasheet NF and current-dependent) produce residuals with $\sigma < 0.1$ mW, approximately $10^4\times$ smaller than the phenomenological models. Dashed lines show Gaussian fits.

The main text identifies noise accumulation through cascaded layers as a scaling concern: ASE from first-layer SOAs is re-amplified by downstream SOAs, and the signal–ASE beat noise compounds at each detection stage. To quantify this beyond the architectures used in the main text, we train a 7-layer [2,2,. . . ,2] network on Two Moons and measure the SNR at each layer output under current-dependent ASE noise (Supplementary Fig. 4). SNR decreases monotonically from ∼67 dB at layer 0 to ∼49 dB at layer 6. Each layer adds ASE, but re-amplification maintains the signal well above the noise floor. At depth 7 the accumulated SNR remains above the 14 dB phenomenological threshold by more than 35 dB.

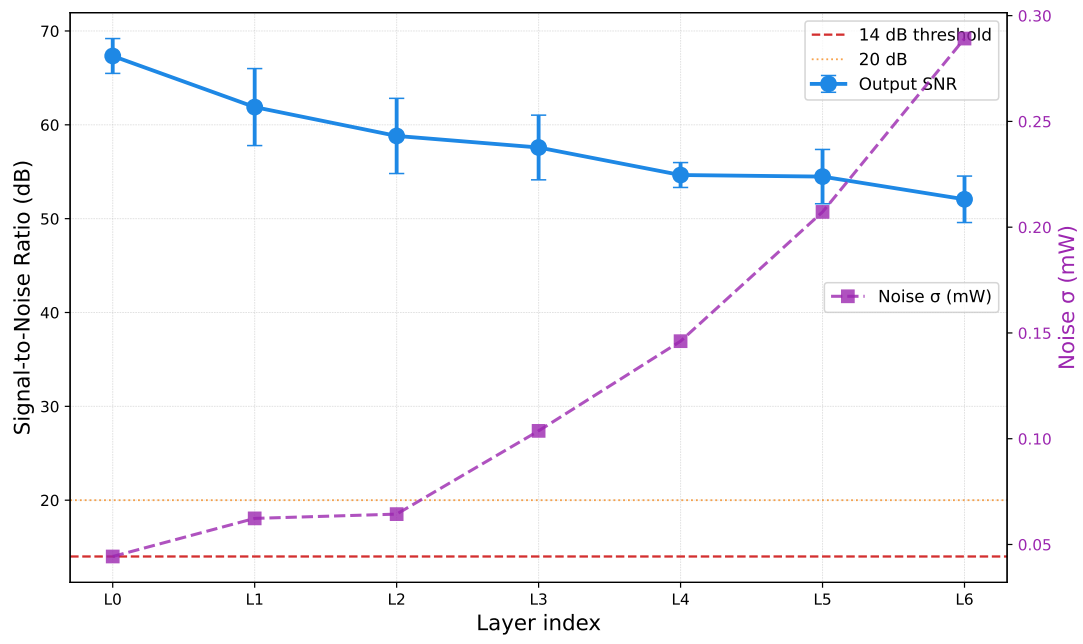

Supplementary Figure 4: SNR at each layer output through a 7-layer [2,2,. . . ,2] cascade under current-dependent ASE noise (10 seeds, mean ± std). The dashed line marks the 14 dB phenomenological threshold from the main text. SNR decreases from ∼67 dB to ∼49 dB across seven layers but remains above the threshold by more than 35 dB.

The amplitude-domain model in Supplementary Table 5 shows the strongest signal dependence: noise power scales with signal power, so high-power signals are penalised disproportionately. The same dependence appears in the physics-based models through the signal–ASE beat term ($\sigma^2_{\text{sig-sp}} \propto P_{\text{signal}}$, Eq. (19)). To quantify this, we sweep input power from 10 to 80 mW through a trained [2,2] module and record output variance over 1000 noise realisations per model (Supplementary Table 6, Supplementary Fig. 5). The physics-based models produce output variance approximately 1.4×10^4 times smaller than the phenomenological power-domain model. Both exhibit $\sigma^2 \propto P_{\text{signal}}$, confirming the heterodyne beat mechanism, but the maximum output standard deviation under current-dependent noise is ∼0.03 mW, compared with signal powers of 10–80 mW.

Supplementary Table 6: Output variance range across input power for each noise model. The physics-based models produce variance approximately $10^4\times$ smaller than the phenomenological power-domain model.

Noise model	Min var. [mW^2]	Max var. [mW^2]	Ratio	Signal dependence
Power 20 dB	4.16×10^{-1}	5.46×10^{-1}	1.3	Weak
Amplitude 20 dB	1.70×10^{1}	2.52×10^{1}	1.5	Moderate
Datasheet NF 8 dB	1.13×10^{-3}	1.78×10^{-3}	1.6	$\sigma^2 \propto P_{\text{signal}}$
Current-dependent	8.89×10^{-4}	1.15×10^{-3}	1.3	$\sigma^2 \propto P_{\text{signal}}$

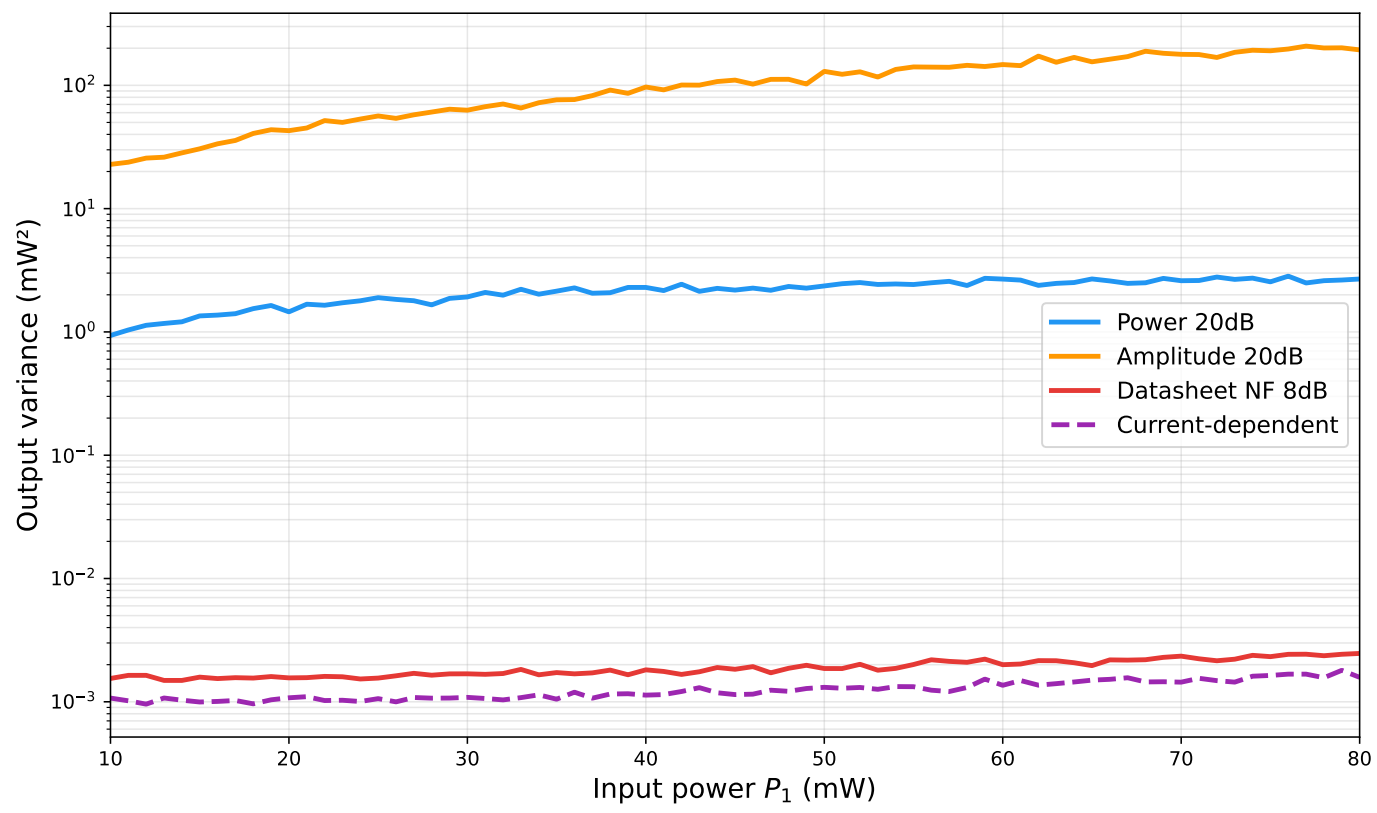

Supplementary Figure 5: Output variance versus input power for four noise models. The physics-based models (bottom curves) show variance proportional to signal power, consistent with signal–ASE beat noise. Log-scale y-axis.

In summary, physical ASE noise is negligible at the CW operating point. Even in a 7-layer cascade, the SNR remains above 49 dB, more than 35 dB above the phenomenological threshold where performance degrades. The phenomenological power-domain model at 14 dB SNR is approximately $10^2\times$ more pessimistic than the physical ASE floor and is a conservative stress test covering impairment sources beyond ASE alone.

Supplementary Note 5: Experimental realisation details

This section details the practical considerations for experimental realisation of SSP-KAN: SOA characterisation procedures, interferometric phase stability, and the minimal experimental configuration.

Supplementary Note 5.1: SOA characterisation and model calibration

The Agrawal–Olsson model used in the main text (Eq. (5) of the main text) is parameterised by three device-level quantities: saturation power P_{sat}, maximum small-signal gain $G_{\max}$ (equivalently $h_{0,\max}$), and transparency current I_{tr}. For the Thorlabs BOA1554P, the datasheet specifies $G_{\max} \approx 27$ dB (small-signal, fibre-to-fibre), saturation output power $P_{\text{sat,out}} = 18.5$ dBm, and operating current range 600–1700 mA [6]. The simulations in the main text use $G_{\max} = 35$ dB, $P_{\text{sat}} = 18$ dBm, and $I_{\text{tr}} = 600$ mA; these values represent the gain within the waveguide (before fibre coupling loss) and should be refined from measured data.

Extraction procedure.

Each SOA module is characterised by measuring output power P_{out} as a function of input power P_{in} at several injection currents spanning 600–1700 mA. The transparency current I_{tr} is the bias at which fibre-to-fibre transmission crosses 0 dB. The small-signal gain $G_{\max}(I)$ is obtained from $P_{\text{out}}/P_{\text{in}}$ in the low-power limit ($P_{\text{in}} \ll P_{\text{sat}}$) at each current. The saturation power P_{sat} follows from the 3 dB compression point: the Agrawal–Olsson model gives $P_{\text{sat}} \approx P_{3\text{dB}}/\ln 2$. The gain coefficient $\kappa = h_{0,\max}/(I_{\max}/I_{\text{tr}} - 1)$ then determines the current-to-gain mapping (Eq. (10) of the main text).

Linewidth enhancement factor.

The variability study (Supplementary Note 7) identifies α_H as the single most sensitive device parameter. Extracting α_H per device is therefore critical for deployment. The standard method modulates the SOA injection current at a frequency ω and measures the resulting amplitude modulation (AM) and phase modulation (PM) on the output signal simultaneously. The ratio of PM to AM indices gives α_H directly: $\alpha_H = -2\,(\Delta\phi/\Delta G)$, where $\Delta\phi$ is the phase modulation depth and ΔG is the fractional gain modulation. This measurement requires only a coherent receiver or a fibre interferometer and is routinely performed for SOA and laser characterisation. For the BOA1554P, α_H is expected to lie in the range 3–7 depending on wavelength and bias current, consistent with the sampling range used in the variability study.

Fine-tuning protocol.

Once all five device-level constants (P_{sat}, $G_{\max}$, I_{tr}, α_H, and the gain coefficient κ) are measured, the four per-module trainable parameters (I, α_1, α_2, ϕ) can be re-optimised via backpropagation through the differentiable model with the measured constants substituted. This fine-tuning pass compensates for residual model–device mismatch (e.g. from wavelength dependence, temperature sensitivity, or the linear-gain approximation) without requiring a full retraining of the network.

Supplementary Note 5.2: Phase stability

SSP-KAN requires active phase stabilisation. The MZI phase ϕ is a trained parameter; any drift from the trained set-point distorts the transfer function and degrades inference accuracy.

Active MZI stabilisation is a mature technology in both integrated and fibre-optic platforms. Programmable photonic circuits based on MZI meshes routinely stabilise tens to hundreds of interferometers using on-chip thermal tuners and local feedback loops [7, 8]. SSP-KAN requires stabilising only 4–7 MZIs, far fewer than typical programmable processors. For fibre-pigtailed implementations, piezoelectric fibre stretchers driven by PID controllers achieve sub-radian phase stability at kHz servo bandwidths, as demonstrated over 1 km fibre paths [9]. The bench-scale SSP-KAN modules present a less demanding stabilisation target.

A concern specific to SSP-KAN is that the SOA introduces a power-dependent phase shift $\Delta\phi_{\mathrm{SOA}} = -\alpha_H h/2$ that varies with the input signal. This could in principle corrupt a dither-based stabilisation loop. The relevant timescales are, however, separated by more than four orders of magnitude. Environmental drift (thermal, mechanical) operates at 1–100 Hz. The dither frequency for phase stabilisation is typically 1–10 kHz. The signal-dependent phase modulation follows the input at MHz to GHz rates, bounded by the CW validity condition $B_e \ll 1/\tau_c \approx 2$–5 GHz (Eq. 20). Because the stabilisation loop integrates over many signal periods, the power-dependent phase shift averages to its mean value and does not corrupt the feedback signal. The loop tracks only the slowly varying geometric phase offset between the two MZI arms.

Stabilisation overhead scales linearly with module count. For the 4–7 module configurations targeted here, standard TEC modules and low-bandwidth feedback are sufficient. Transition to photonic integrated circuits becomes relevant for architectures requiring hundreds of modules.

Supplementary Note 5.3: Minimal experimental setup

The [2,2] Two Moons classifier requires a tunable CW laser source ($P_{\mathrm{out}} \geq 20$ mW, C-band), two DAC-controlled variable optical attenuators for input encoding (0–30 dB, $\geq$8-bit resolution), two 1:2 fibre-optic couplers for input fan-out, four MZI-VOA-SOA-VOA modules, two 2:1 couplers for output summation, and two InGaAs photodetectors at the output nodes. Each module comprises two 50/50 directional couplers, one SOA (Thorlabs BOA1554P or equivalent), two variable optical attenuators, and one fibre stretcher or thermo-optic phase shifter for the MZI phase ϕ. Four TEC modules (one per MZI) provide phase stabilisation, and a digital control interface (FPGA or microcontroller with DAC outputs) programs the current sources and VOA drivers.

All components are commercially available as fibre-pigtailed, connectorised devices. The total component cost is estimated at \$5,000–10,000, dominated by the four SOAs at approximately \$1,000 each. No cleanroom access, custom photonic chip fabrication, or free-space optical alignment is required.

Supplementary Note 6: Gradient-free training results

When the differentiable physics model is insufficiently accurate or the physical channel is unknown, gradient-free optimisation can train SSP-KAN directly from measured network outputs. We evaluate Covariance Matrix Adaptation Evolution Strategy (CMA-ES) [10] as a representative gradient-free optimiser.

Supplementary Note 6.1: Experimental setup

CMA-ES maintains a multivariate Gaussian search distribution over the parameter space (16 dimensions for [2,2], 28 for [6,1,1]) and adapts both its mean and covariance at each generation. We use initial step size $\sigma_0 = 0.3$, population size $\lambda = 20$ (Moons) or $\lambda = 24$ (yacht), and run for 200 generations (Moons) or 300 generations (yacht). Each generation requires λ forward-pass evaluations of the physical network. The total evaluation budget is therefore 4,000 (Moons) and 7,200 (yacht).

Supplementary Note 6.2: Results

Supplementary Table 7: CMA-ES versus Adam+backpropagation. CMA-ES achieves comparable mean performance with substantially lower variance across seeds.

Task	Optimiser	Mean metric	Std	Best	Worst
Moons [2,2]	Adam (500 epochs)	94.2%	1.0%	95.8%	92.4%
Moons [2,2]	CMA-ES (4,000 evals)	93.8%	0.0%	93.8%	93.8%
Yacht [6,1,1]	Adam (1,000 epochs)	$R^2 = 0.881$	0.198	0.983	0.447
Yacht [6,1,1]	CMA-ES (7,200 evals)	$R^2 = 0.955$	0.048	0.986	0.879

On Two Moons, CMA-ES achieves 93.8% accuracy with zero variance across 5 seeds, compared with Adam's 94.2% ± 1.0%. The 0.4 percentage point difference in mean accuracy is offset by CMA-ES's complete elimination of seed-dependent variability.

On yacht hydrodynamics, CMA-ES achieves $R^2 = 0.955 \pm 0.048$, substantially outperforming Adam's $R^2 = 0.881 \pm 0.198$ in both mean and worst-case performance. Adam's high variance (worst seed: $R^2 = 0.447$) indicates sensitivity to initialisation in this higher-dimensional parameter space (28 parameters); CMA-ES's covariance adaptation provides more robust convergence.

These results demonstrate that SSP-KAN can be trained without backpropagation at a cost of 4,000–7,200 forward-pass evaluations. For a physical network operating at CW with microsecond-scale measurement times, this corresponds to total training times on the order of seconds to minutes, making on-site training practical for laboratory deployment.

Supplementary Note 7: Device parameter variability

Each MZI-VOA-SOA-VOA module depends on four device-level constants that vary across manufactured devices: saturation power P_{sat}, maximum small-signal gain G_{max}, transparency current I_{tr}, and linewidth enhancement factor α_H. Training uses nominal datasheet values (Supplementary Table 8). A deployed device will differ from these values. This section quantifies the sensitivity of inference accuracy to device-to-device variation on the yacht [6,1,1] regression task and identifies which parameters require per-device characterisation. When the device parameters are known, or are determined by a single routine characterisation step, training with the measured constants yields $R^2 \geq 0.955$ on every condition tested.

Supplementary Table 8: Device parameter sampling ranges. Nominal values correspond to the Thorlabs BOA1554P parameters used throughout the main text.

Parameter	Nominal	Sampling range	Units
G_{max}	35.0	$\mathcal{U}[32.0,\ 35.0]$	dB
P_{sat}	18.0	$\mathcal{U}[17.0,\ 19.0]$	dBm
I_{tr}	600	$\mathcal{U}[\text{nom}\cdot(1-f),\ \text{nom}\cdot(1+f)]$	mA
α_H	5.0	$\mathcal{U}[3.0,\ 7.0]$	—

Eight perturbation conditions are tested: nominal (no perturbation), five single-parameter ablations (G_{max}, P_{sat}, α_H, I_{tr} at $\pm 5\%$, and I_{tr} at $\pm 10\%$), and two combined conditions (all four parameters varied simultaneously, I_{tr} spread $f = 5\%$ or 10%). Two deployment scenarios are evaluated. In the first, each module's device constants are sampled at model initialisation and the optimiser trains with them in place, simulating deployment with a known variability profile. In the second, the model is trained on nominal parameters and then evaluated under 50 Monte Carlo device draws per condition, simulating direct transfer to an uncharacterised device. Five random seeds are used throughout.

Supplementary Note 7.1: Variability-aware training

When the optimiser sees the actual device parameters during training, it compensates. Supplementary Table 9 reports yacht [6,1,1] R^2 across 5 seeds per condition, using the sigmoid reparameterisation described in Methods. All eight perturbation conditions produce $R^2 \geq 0.955$, with the worst case being the combined ($f = 5\%$) condition ($R^2 = 0.955 \pm 0.062$). The linewidth enhancement factor α_H remains the most sensitive single-parameter axis ($R^2 = 0.961 \pm 0.047$), but variability-aware training compensates its effect to within 0.4 percentage points of the nominal R^2. Without the sigmoid reparameterisation (i.e. using softplus with post-hoc clamping), three conditions show marked degradation (α_H: $R^2 = 0.778 \pm 0.176$; combined $f = 10\%$: 0.839 ± 0.275; P_{sat}: 0.875 ± 0.199), driven by seeds that converge to narrow minima sensitive to the sampled device parameters. The sigmoid reparameterisation eliminates this failure

mode by constraining each trainable parameter to its physical operating range through a smooth bijection (Methods), preventing the optimiser from reaching parameter-scale configurations that are fragile under device variation.

Supplementary Table 9: Yacht [6,1,1] R^2 when training with device variability (5 seeds per condition, sigmoid reparameterisation). Device parameters are sampled per module at model initialisation; the optimiser compensates during training.

Condition	Yacht [6,1,1] R^2
Nominal	0.965 ± 0.041
G_{max} ablation	0.976 ± 0.019
P_{sat} ablation	0.974 ± 0.024
$I_{\mathrm{tr}} \pm 5\%$	0.986 ± 0.003
$I_{\mathrm{tr}} \pm 10\%$	0.972 ± 0.028
α_H ablation	0.961 ± 0.047
Combined ($f = 5\%$)	0.955 ± 0.062
Combined ($f = 10\%$)	0.974 ± 0.020

Supplementary Note 7.2: Deployment without recalibration

The more demanding scenario is deploying a nominally trained model on hardware whose device constants differ from the training values, with no characterisation of any kind; this scenario quantifies why the calibration step is needed, rather than describing an intended operating mode. Supplementary Fig. 6 ranks the eight conditions by mean R^2 across 250 evaluations (5 seeds × 50 Monte Carlo device draws). The α_H ablation dominates (mean $R^2 = -145$): the trained MZI phase ϕ no longer matches the device's α_H-dependent SOA phase shift. The combined conditions follow the α_H contribution (mean $R^2 = -98$ to -101). The sigmoid reparameterisation improves variability-aware training (Section 7.1) but cannot substitute for measuring α_H, because the mismatch is physical rather than numerical; the standard gain–phase measurement (Supplementary Note 5) restores the trained interference condition.

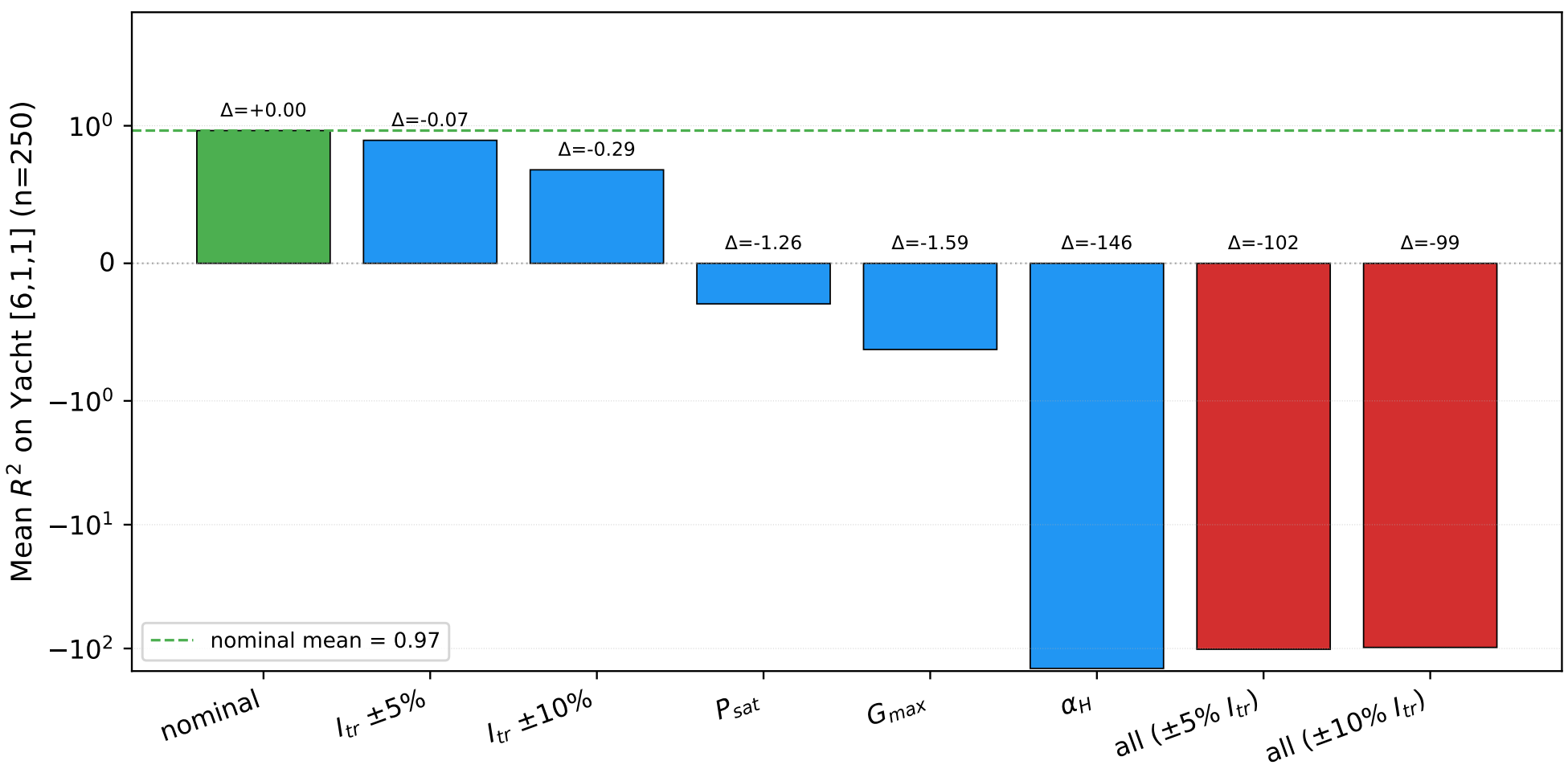

Supplementary Figure 6: Sensitivity of yacht [6,1,1] R^2 to single-parameter and combined device variation when deploying a nominally trained model without recalibration (250 evaluations per condition). α_H, sampled over ±40% of its nominal value, dominates all other parameter axes by two orders of magnitude. Sampling ranges for the remaining parameters are listed in Supplementary Table 8.

Supplementary Fig. 7 shows the full R^2 distributions. For α_H and the combined conditions, the 95th percentile remains negative, so the failure is not confined to a tail of extreme fabrication outcomes. $P_{\rm sat}$ and $G_{\rm max}$ produce moderate degradation with heavy-tailed distributions: their median R^2 values (0.42, 0.12) substantially exceed the means (−0.30, −0.63). $I_{\rm tr} \pm 5\%$ is the only perturbed condition that remains tolerable (mean $R^2 = 0.89$, median $R^2 = 0.95$).

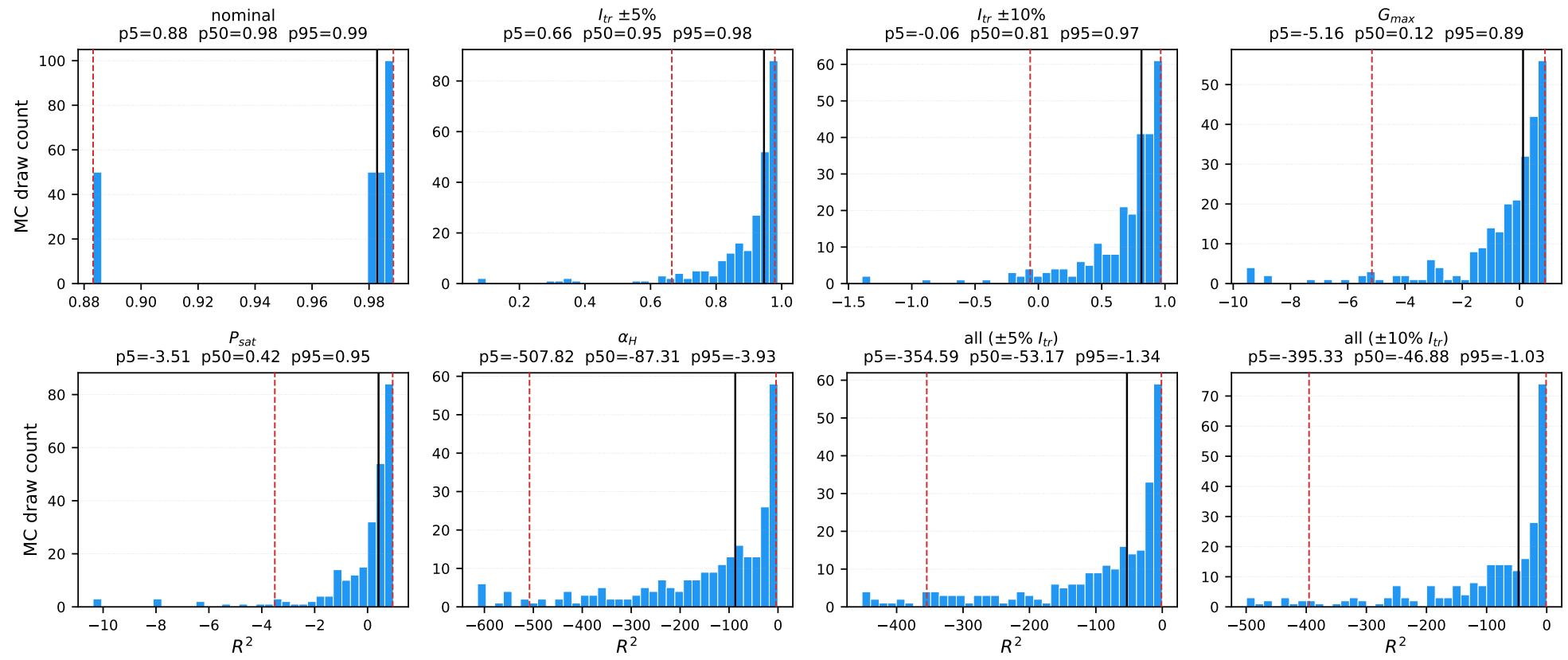

Supplementary Figure 7: Distribution of yacht [6,1,1] R^2 across 250 Monte Carlo device draws per condition, for a nominally trained model deployed without recalibration. Solid vertical lines mark the median; dashed red lines mark the 5th and 95th percentiles. The α_H, combined ($\pm 5\%$ I_{tr}), and combined ($\pm 10\%$ I_{tr}) conditions produce negative R^2 in more than 98% of draws.

Supplementary Note 7.3: Physical origin and mitigation

The α_H sensitivity has a direct physical origin. The linewidth enhancement factor enters the MZI interference condition through the gain-induced phase shift $\Delta\phi_{\mathrm{SOA}} = -\alpha_H h/2$ (Methods). Training selects MZI phases ϕ jointly with the nominal $\alpha_H = 5$. Sampling α_H in [3, 7] shifts every interference condition by $\Delta\phi = -(\Delta\alpha_H/2)\cdot h$. For a typical integrated gain $h \approx 3$, this produces phase errors of order ± 3 rad, enough to move the operating point from a constructive to a destructive MZI fringe. In contrast, P_{sat} and G_{max} shift the gain saturation curve horizontally or vertically without altering the phase condition, preserving the qualitative shape of the transfer function.

The $\pm 40\%$ α_H range used here ([3, 7] around nominal 5) is deliberately conservative. Telecom-grade SOAs are manufactured to tight specifications, and device-to-device α_H variation within a single product line is typically below $\pm 10\%$. The wider range is sampled because α_H, unlike P_{sat}, G_{max}, and I_{tr}, is rarely quoted on manufacturer datasheets and must be estimated. The [3, 7] range spans the full spread reported across different SOA material systems and wavelength bands in the literature, not the variation expected within a batch of identical devices. The failure observed under this range therefore represents a worst case that is unlikely to occur in practice with a single SOA product.

Two complementary mitigations eliminate this failure mode. First, per-device characterisation of α_H from a standard gain–phase measurement (see Supplementary Note 5) ensures that the forward model accounts for the actual gain–phase coupling. Second, the sigmoid reparameterisation (Methods) constrains each trainable parameter to its physical range through a smooth bijection, preventing the optimiser from reaching fragile parameter-scale configurations. With both mitigations in place,

variability-aware training produces $R^2 \geq 0.955$ across all eight perturbation conditions (Supplementary Table 9), including the α_H ablation, the most sensitive axis.

The variability study also bounds the effect of operating at different wavelengths. Shifting the source away from the SOA gain peak changes all four device constants simultaneously: the gain $G_{\max}(\lambda)$ and noise figure $\mathrm{NF}(\lambda)$ follow the material gain spectrum, $P_{\mathrm{sat}}(\lambda)$ tracks the wavelength-dependent confinement factor, and $\alpha_H(\lambda)$ varies with the detuning from the band edge. For the BOA1554P, the datasheet specifies ∼3 dB gain variation across the 80 nm C-band (1520–1600 nm). This is comparable to the $G_{\max}$ ablation range used here ([32, 35] dB, i.e. 3 dB one-sided), and falls within the regime where the nominally trained model remains functional (Supplementary Table 9). Multi-wavelength operation therefore requires per-channel characterisation and retraining with the wavelength-resolved Agrawal–Olsson parameters, but does not introduce a qualitatively new failure mode beyond those already captured by the single-parameter sweeps.

This study samples each module's device constants independently. Spatially correlated fabrication variation (e.g. a systematic carrier-density gradient across a photonic die) and temperature-induced parameter drift are not modelled. Independent sampling overestimates the spread that a real production batch would exhibit, so the deployment results represent a conservative bound on degradation.

References

[1] Henry, C.H.: Theory of spontaneous emission noise in open resonators and its application to lasers and optical amplifiers. Journal of Lightwave Technology **LT-4**(3), 288–297 (1986) https://doi.org/10.1109/JLT.1986.1074707

[2] Baveja, P.P., Maywar, D.N., Agrawal, G.P.: Pulse amplification in semiconductor optical amplifiers with ultrafast gain recovery. Proceedings of SPIE **7598**, 759817 (2010) https://doi.org/10.1117/12.841107

[3] Desurvire, E.: Erbium-Doped Fiber Amplifiers: Principles and Applications. Wiley-Interscience, New York (1994)

[4] Personick, S.D.: Applications for quantum amplifiers in simple digital optical communication systems. Bell System Technical Journal **52**(1), 117–133 (1973) https://doi.org/10.1002/j.1538-7305.1973.tb01901.x

[5] Mukai, T., Yamamoto, Y.: Noise in an AlGaAs semiconductor laser amplifier. IEEE Journal of Quantum Electronics **QE-18**(4), 564–575 (1982) https://doi.org/10.1109/JQE.1982.1071586

[6] Thorlabs, Inc.: BOA1554P — C-band booster optical amplifier, datasheet. https://www.thorlabs.com/thorproduct.cfm?partnumber=BOA1554P (2024)

[7] Bogaerts, W., Pérez, D., Capmany, J., Miller, D.A.B., Poon, J., Englund, D., Morichetti, F., Melloni, A.: Programmable photonic circuits. Nature **586**, 207–216

(2020) https://doi.org/10.1038/s41586-020-2764-0

[8] Pérez, D., Gasulla, I., Crudgington, L., Thomson, D.J., Khokhar, A.Z., Li, K., Cao, W., Mashanovich, G.Z., Capmany, J.: Multipurpose silicon photonics signal processor core. Nat. Commun. **8**, 636 (2017) https://doi.org/10.1038/s41467-017-00714-1

[9] Xavier, G.B., Weid, J.P.: Stable single-photon interference in a 1 km fiber-optic Mach–Zehnder interferometer with continuous phase adjustment. Opt. Lett. **36**(10), 1764–1766 (2011) https://doi.org/10.1364/OL.36.001764

[10] Hansen, N., Ostermeier, A.: Completely derandomized self-adaptation in evolution strategies. Evolutionary Computation **9**(2), 159–195 (2001) https://doi.org/10.1162/106365601750190398
