# Supplementary material for: Small-scale photonic Kolmogorov-Arnold networks using standard telecom nonlinear modules
Source: Nat Commun. 2026 Jul 21;17:8938. doi: 10.1038/s41467-026-75602-8 (PMC13503846; doi:10.1038/s41467-026-75602-8)
Supplement: Supplementary file 2 [file 41467_2026_75602_MOESM2_ESM.pdf]

Description of Additional Supplementary Files

Supplementary Movie 1. Training evolution of the learned decision boundary of the [2,2] SSPKAN on the Two Moons task under incoherent (power-summation) operation. Each frame shows the decision boundary in the input plane at one training epoch as the four trainable parameters per MZI-VOA-SOA-VOA module are optimised, converging to separate the two crescents.

Supplementary Movie 2. Training evolution of the learned decision boundary of the [2,2,2] SSP-KAN on the Two Moons task under incoherent (power-summation) operation. Each frame shows the decision boundary in the input plane at one training epoch as the four trainable parameters per MZI-VOA-SOA-VOA module are optimised, converging to separate the two crescents.

Supplementary Movie 3. Training evolution of the learned decision boundary of the [2,2,2] SSP-KAN on the Two Moons task under coherent (field-summation) operation. Each frame shows the decision boundary in the input plane at one training epoch as the four trainable parameters per MZI-VOA-SOA-VOA module are optimised, converging to separate the two crescents.
