# Supplementary material for: Small-scale photonic Kolmogorov-Arnold networks using standard telecom nonlinear modules
Source: Nat Commun. 2026 Jul 21;17:8938. doi: 10.1038/s41467-026-75602-8 (PMC13503846; doi:10.1038/s41467-026-75602-8)
Supplement: Supplementary file 3 [file 41467_2026_75602_MOESM6_ESM.pdf]

nature portfolio

Peer Review File

Small-scale photonic Kolmogorov-Arnold networks using standard telecom nonlinear modules

Corresponding Author: Mr Luca Calcado

This file contains all reviewer reports in order by version, followed by all author rebuttals in order by version.

Version 0:

Reviewer comments:

Reviewer #1

(Remarks to the Author)

```
\documentclass[a4paper,pre,preprint,showpacs,amsmath,amssymb]{revtex4-1}

\usepackage{graphicx}
\usepackage{amsmath}

\DeclareMathOperator{\sech}{sech}
\DeclareMathOperator{\csch}{cosech}
\DeclareMathOperator{\Real}{Re}
\DeclareMathOperator{\Imag}{Im}
\newcommand{\dx}{\, dx}
\newcommand{\conj}[1]{{#1}^{\ast}}
\newcommand{\conjl}[1]{\overline{#1}}
\newcommand{\order}[1]{{\cal O}\left({#1}\right)}

\begin{document}

\noindent
{\bf Paper: Small-scale photonic Kolmogorov-Arnold networks ...
\\
Authors: Luca Nogueira Calcado et al \\ Ref: NCOMMS-26-022249
}
\begin{center}
{\bf Referee's report}
\end{center}
```

The authors introduce small-scale photonic Kolmogorov-Arnold networks that are implemented entirely with standard telecommunications components. Each network edge employs a trainable nonlinear module composed of a Mach-Zehnder interferometer, semiconductor optical amplifier, and variable optical attenuator, providing a four-parameter transfer function derived from gain saturation and interferometric mixing.
The authors demonstrate that physically constrained nonlinear transfer function realized via the module can serve as trainable edge nonlinearity in the network architectures, enabling nonlinear inference across classification, regression, and image recognition tasks.

The study is clearly positioned at the interface of nonlinear photonics, optical computing, and emerging physics-based computing concepts.
The authors address a key limitation in photonic neural networks: the reliance on electronic nonlinearities and associated optical-electrical-optical bottlenecks.

By proposing a fully optical, differentiable, and hardware-realistic architecture, they advance both conceptual understanding and practical feasibility.

The paper is likely to be of interest to a broad community spanning photonics and optics-based computation.
The reported results demonstrate high accuracy in nonlinear classification (98.4\%) and regression ($R^2 \approx 0.977$), as well as
reselience to noise and quantisation. In particular, the networks maintain stable performance down to approximately 6-bit inputs and moderate
signal-to-noise ratio. The conclusions are supported by systematic comparisons with software Kolmogorov-Arnold network and multilayer perceptron baselines, and by clear physical modelling grounded in realistic device parameters.

This is a timely and conceptually significant contribution that introduces a practical and physically grounded photonic Kolmogorov-Arnold network architecture,.
The very idea of trading off generality against implementability using small-scale networks is, in itself, worthy of discussion by the community.

The paper
bridges theory, simulation, and hardware feasibility. The material is presented logically and coherently.
I would be happy to recommend publication once the authors have taken care of the following critique. \

{\bf Essential criticisms}

\begin{enumerate}

\item
While the motivation for
the Kolmogorov-Arnold networks and their photonic implementation is clearly articulated,
the novelty against prior photonic neural networks and recent photonic Kolmogorov-Arnold demonstrations could be sharpened. In particular, several aspects would benefit from further clarification:

\begin{enumerate}

\item
The distinction between small-scale photonic Kolmogorov-Arnold networks
and prior photonic multilayer perceptron implementations is well explained.
However, the difference with respect to recent photonic Kolmogorov-Arnold implementations (e.g., ring-assisted Mach-Zehnder interferometer-based systems) should be articulated more explicitly.

\item
The present study is entirely simulation-based (albeit using realistic parameters).
Additional discussion of experimental feasibility would strengthen the manuscript.

\item
In the Discussion section, the authors identify “primary practical challenges” as (a) semiconductor optical amplifier characterisation and (b) phase stability. A more detailed analysis of these issues would be valuable.

\item
It would be helpful to outline a minimal experimental implementation and to specify expected stability requirements (e.g., phase drift tolerance and calibration frequency). In addition, the authors should clarify whether training is intended to be

performed in situ or offline, how trained parameters can be transferred to hardware, and how sensitive performance is to parameter mismatch.

\end{enumerate}

\item
The authors correctly note that large-scale architectures (e.g., MNIST with $\sim 10^4$ modules) are impractical in fibre-based implementations. However, the discussion of scalability could be made more forward-looking. In particular, it would be useful to comment on whether performance gains saturate with network depth and width, to outline possible integration pathways, and to discuss scaling limits imposed by loss, noise accumulation, and phase stability.

\item
The authors adopt a power-domain noise model for the interpretation of the amplified spontaneous emission.
While reasonable, this constitutes a simplification; furthermore, the amplitude-domain model is mentioned but not explored.
The authors should justify more explicitly why the power-domain model is sufficient, and comment on whether the conclusions would change in the presence of signal-dependent noise or phase noise, which may be particularly relevant for interferometric operation.

\end{enumerate}

{\bf Secondary points}

\begin{enumerate}

\item
The authors should clarify the relationship between training and deployment, and indicate whether training assumes perfect knowledge of device parameters. Is hardware-in-the-loop training required in practice?

\item
The study highlights a reduced number of parameters compared to software Kolmogorov-Arnold networks.
It would be useful to include an estimate of the energy per inference, or at least a qualitative comparison with electronic neural networks.
In addition, the transfer functon(equation (15)), which is central to the work, is somewhat understated; further discussion would help to highlight its importance, particularly in relation to nonlinear science.

\end{enumerate}

{\bf Editorial criticisms and suggestions}

\begin{enumerate}

\item
The authors should consider explicitly labelling the “trainable parameters” in the diagram of
Fig.~1.

\item
The authors should
ensure consistency of notation. For instance, the same notation ϕ is
used for a function in equation (2) and for a phase in sections 3.6-3.7.

\item Line 520: a reference is missing.

\item
The paper is already well referenced, but I would suggest to consider mentioning some recent publications, e.g.
Feldmann, J., Youngblood, N., Karpov, M. et al. Parallel convolutional processing using an integrated photonic tensor core. Nature 589, 52–58 (2021).
Xu, X., Tan, M., Corcoran, B. et al. 11 TOPS photonic convolutional accelerator for optical neural networks. Nature 589, 44–51 (2021). Neuromorphic photonics / nonlinear optics for computing
Miscuglio \& Sorger, Photonic tensor cores for machine learning, Appl. Phys. Rev. 7, 031404 (2020)

\end{enumerate}

\end{document}

(Remarks on code availability)
The code runs without trouble on my Mac computer.

Reviewer #2

(Remarks to the Author)
The manuscript proposes a Small-Scale Photonic Kolmogorov–Arnold Network (SSP-KAN) architecture built entirely from standard telecom fiber-optic components (Mach-Zehnder interferometer (MZI) couplers, variable optical attenuators (VOAs), and semiconductor optical amplifiers (SOA)) and trained end-to-end with a differentiable physics model. Key results include 98.4% accuracy on the nonlinear "Two Moons" classification, a regression R^2 of ≈0.91–0.94 on the yacht hydrodynamics dataset, and improvements on image tasks. The contribution of this work is currently stronger at the implementation level than at the conceptual or methodological level. The core idea is to realize KAN-style edge nonlinearities with telecom-grade MZI-SOA-VOA modules, but the manuscript does not yet demonstrate a sufficiently deep theoretical advance beyond a hardware mapping of an existing KAN concept.

Here are my comments:
1. The article is based on the Kolmogorov–Arnold network (KAN), but the KAN framework itself is not the author's original creation. The innovation of this work is mainly in the use of optical modules (MZI, VOA, SOA) to implement KAN, but similar ideas of using optical nonlinear elements to build neural networks have been studied in advance [1,2]. The innovation of this paper is mainly reflected in the engineering implementation level, rather than proposing a completely new architecture or theory. Therefore, at the scientific level, there is a lack of substantial expansion of the KAN model or learning method.

2. The manuscript models the SOA response in the steady state and derives a static transfer function for each MZI-VOA-SOA-VOA module, which is then used for end-to-end training and inference. However, "ultrafast inference" is an architectural claim that cannot be supported by steady-state simulations alone, because it depends on the temporal response of the SOA, the recovery dynamics, the modulator bandwidth, the photodetector response, and any inter-symbol or inter-sample memory effects. The current analysis does not address pattern dependence, transient gain saturation, or time-domain settling behavior, all of which are critical for determining whether the device can truly operate at high speed in realistic conditions.

3. The optical loss budget and system-level efficiency are not quantified. Although the manuscript correctly notes that OEO conversion is a bottleneck in conventional photonic neural networks, the proposed architecture does not eliminate optical loss; rather, it redistributes it across MZI couplers, VOAs, SOAs, and photodetection-based summation.

4. The manuscript observes that deeper architectures can improve expressivity but may also amplify noise. However, this trade-off is not analyzed in depth. In particular, nonlinear systems such as SOA-based modules can introduce distortion that is not simply additive noise, but signal-dependent and potentially non-Gaussian. When cascaded across layers, such distortions may accumulate in a highly nontrivial manner. A more detailed study of noise propagation would provide valuable insight into the limits of depth and the optimal operating regime.

5. The validation tasks may not fully support the claimed generality of the approach. The manuscript evaluates the system on standard benchmarks such as Two Moons, yacht hydrodynamics, and MNIST/Fashion-MNIST. While these are useful for initial validation, they are relatively low-complexity tasks compared to modern machine learning workloads. For example, MNIST is known to be nearly linearly separable, and the observed performance gains over linear models are modest.

6. Since the architecture relies on cascaded nonlinear transfer functions, even small parameter perturbations may accumulate across layers and significantly alter the learned mapping. The manuscript should therefore include a robustness study under device variability

7. The results are presented mostly as single accuracy or R^2 values without measures of variability. At the device or chip level, what about energy and footprint, such as energy per inference and compute density?

8. The impact of wavelength dependence and spectral effects is not considered. The model implicitly assumes a single-wavelength (or wavelength-independent) operation, but in practical telecom systems, components such as SOAs and MZIs exhibit wavelength-dependent gain, phase response, and loss. This becomes particularly relevant if the architecture is extended to wavelength-division multiplexing scenarios, which are a natural direction for scaling photonic neural networks.

[1] Peng, Y., et al., Photonic KAN: a Kolmogorov-Arnold network inspired efficient photonic neuromorphic architecture. arXiv preprint arXiv:2408.08407, 2024.
[2] Sozos, K., et al. Photonic Kolmogorov-Arnold networks based on self-phase modulation in nonlinear waveguides. Optics Letters, 51(3), 664-667, 2026.

(Remarks on code availability)
The code is readable, the overall logic is fairly reasonable, and it effectively reproduces some of the results from the paper.

Reviewer #3

(Remarks to the Author)
1. The authors employ a single MZI-VOA-SOA-VOA module to realize nonlinear functionalities for the KAN. However, as with prior photonic KAN studies, this module contains only a very limited number of tunable parameters. It remains unclear how these four parameters can effectively implement nonlinear functions that typically require hundreds of learnable coefficients — such as those in B-spline representations. Figure 1(b) displays a family of curves but does not constitute sufficiently compelling evidence on its own. Rigorous mathematical proof is essential to substantiate the claimed nonlinear trainability of the proposed module.
2. The authors acknowledge that realistic deployment requires 8-bit DAC resolution and SNR ≥ 14 dB. These are non-trivial requirements that demand significant power budgets in both the electronic and optical domains. This raises a fundamental question: does the proposed SSP-KAN offer a genuine advantage over conventional MLPs when the stringent deployment constraints and associated energy consumption are taken into account? A fair and thorough comparison should be provided.
3. This also leads to a following question. The VOA-SOA-VOA configuration will inevitably degrade SNR due to amplified spontaneous emission (ASE) noise. This concern becomes increasingly severe as the SSP-KAN scales to larger network sizes. The authors should provide quantitative analysis and simulation results to assess whether SNR ≥ 14 dB remains achievable in realistic, large-scale implementations.
4. This work is entirely simulation-based, with no experimental chip fabrication or characterization. Given the highly competitive standard of Nature Communications, a purely theoretical and simulation study of this nature is insufficient for publication in this venue.
5. The chosen tasks, such as Two Moons and MNIST, are well below the current frontier of photonic machine learning. The community expects demonstrations on more complex and contemporary benchmarks to substantiate claims of practical relevance and competitiveness.
6. The SOA and VOA elements require integrated III-V components. While certain foundries, such as Tower Semiconductor, have demonstrated this capability, device-to-device variation poses a serious concern that has not been addressed. Unlike telecommunications applications where moderate gain variation is tolerable, the proposed architecture exploits the nonlinear characteristics of III-V devices, which requires highly uniform output responses across all SOA and VOA elements for a given set of tunable parameters. Failure to achieve this uniformity would necessitate complex and potentially impractical calibration procedures. The authors must address this issue explicitly.
7. The proposed architecture raises significant concerns regarding energy efficiency that have not been addressed. The MZI-VOA-SOA-VOA module is inherently power-intensive, as nonlinearity comes at a direct energy cost. Given this, an energy efficiency analysis is essential. Specifically, the authors should provide a quantitative comparison between the proposed all-optical nonlinear approach and conventional O/E/O conversion methods in terms of total power consumption per operation. Without such an analysis, it is unclear whether the proposed architecture offers any meaningful energy advantage over existing approaches, undermining one of the key motivations for pursuing photonic implementations of neural networks.

(Remarks on code availability)

Version 1:

Reviewer comments:

Reviewer #1

(Remarks to the Author)
The authors have done a commendable job addressing the concerns raised by all reviewers. In particular, they carried out additional modeling efforts and improved several aspects of the presentation and analysis.

Overall, I am satisfied with the thoroughness of the revision and recommend the manuscript for publication.

I would also like to note that the questions concerning energy estimates, while certainly valid and important, are not specific to this work alone. Similar issues arise in many papers in the field, largely because the relevant analytical framework remains incomplete and no universally accepted methodology has yet been established.

(Remarks on code availability)

Reviewer #2

(Remarks to the Author)
The authors have addressed my concerns. I think it can be accepted now.

(Remarks on code availability)
Yes. The code is well-structured, and the results are largely reproducible as described.

Reviewer #3

(Remarks to the Author)
I appreciate the authors' detailed responses to the previous round of review, and acknowledge that several technical concerns regarding MLPs comparison, and energy analysis have been addressed. However, after careful consideration of the revised manuscript and the point-by-point response, I remain unconvinced that this work meets the novelty and impact threshold required for Nature Communications.

1. Limited algorithmic novelty. The Kolmogorov-Arnold Network (KAN) framework itself is directly adopted from Liu et al. (arXiv:2404.19756, 2025) without modification. The authors place trainable univariate nonlinear functions on network edges following exactly the same computational graph structure as the original KAN formulation. The sole architectural contribution is the substitution of B-spline activation functions with MZI-VOA-SOA-VOA transfer functions . While the authors argue in their response to Reviewer 2 Comment 1 that "demonstrating that SOA gain saturation combined with interferometric mixing also supports KAN inference is a substantive finding about the expressivity of physically constrained activation families," this argument conflates hardware implementation with algorithmic innovation. The expressivity analysis itself (Supplementary Section A, Fig. A1) reveals fundamental limitations: the single-layer [2,2] architecture fails completely on XOR (51.8%, chance level), demonstrating that the constrained four-parameter activation family introduces new failure modes not present in software KANs. These limitations are not adequately theorised; the authors acknowledge in their response to Reviewer 3 Comment 1 that "whether this family is dense in an appropriate function space is an open theoretical question," yet present no approximation-theoretic bounds or convergence guarantees.

2. Incremental differentiation from prior photonic KAN work. Compared to existing photonic KAN implementations, the primary distinction of SSP-KAN is the choice of nonlinear device rather than any new principle. Peng et al. (arXiv:2408.08407) demonstrated photonic KAN using ring-assisted MZI (RAMZI) achieving 98% MNIST accuracy with nine tunable parameters per edge. Sozos et al. (Optics Letters, 2026) implemented photonic KAN via self-phase modulation in nonlinear waveguides. The present work achieves 93.9% MNIST accuracy with four parameters per edge using SOA gain saturation. The authors frame the use of commodity telecom components as the key differentiator. However, the engineering trade-off between hardware accessibility and activation expressivity does not, in itself, constitute a conceptual advance sufficient for Nature Communications.

3. Purely simulation-based validation remains a concern. The authors argue that Nature Communications regularly publishes photonic simulation and theoretical studies when the physics is validated against measured component data. While this is true, the more pertinent question is whether the theoretical contribution itself is of sufficient significance to warrant publication in this venue. In the case of the present manuscript, the work amounts to a simulation study demonstrating the mapping of an existing algorithmic framework onto a specific class of optical hardware; no new theoretical results are derived, no approximation-theoretic guarantees are provided for the four-parameter activation family. Beyond the question of theoretical significance, several critical performance claims rest on assumptions that have not been experimentally verified and that, on closer inspection, are more fragile than the manuscript acknowledges. The SNR analysis in Supplementary Section D reports output SNR values of 49–67 dB across a 7-layer cascade under the CW steady-state assumption, which is valid only when the electrical observation bandwidth satisfies $B_e \ll 1/\tau_c \approx 2$–$5$ GHz (Supplementary Eq. D19). The authors acknowledge this constraint but do not quantify how inference performance degrades as the operating speed approaches this bound. It is a gap that is critical for the latency and bandwidth claims that motivate the photonic implementation in the first place. More seriously, the device variability study in Supplementary Section G demonstrates that deploying a nominally trained model without per-device characterisation of the linewidth enhancement factor α_H results in catastrophic and systematic failure: mean $R^2 = -145$ on the yacht [6,1,1] task, with the 95th percentile of the Monte Carlo distribution remaining negative across all 250 device draws (Supplementary Fig. G7). This is not an edge case but the expected outcome for any device whose α_H differs from the nominal training value of 5, a variation the authors themselves note is routinely ±40% across SOA product lines. The proposed mitigation, per-device α_H characterization via gain-phase measurement, is described only in simulation and has not been demonstrated experimentally. Until it is, the claim that SSP-KAN is practically deployable using commodity telecom hardware remains unsubstantiated.

Overall, were this manuscript submitted to an engineering-focused venue such as Journal of Lightwave Technology, it would

constitute a valuable and publishable contribution: simulation studies grounded in commercial device specifications are standard in these venues, and the engineering contribution of realizing photonic neural computation from off-the-shelf fibre-pigtailed components would receive appropriate recognition. For Nature Communications, however, where conceptual novelty and demonstrated real-world impact are the primary criteria, the manuscript currently falls short.

(Remarks on code availability)

Open Access This Peer Review File is licensed under a Creative Commons Attribution 4.0 International License, which permits use, sharing, adaptation, distribution and reproduction in any medium or format, as long as you give appropriate credit to the original author(s) and the source, provide a link to the Creative Commons license, and indicate if changes were made.
In cases where reviewers are anonymous, credit should be given to 'Anonymous Referee' and the source.
The images or other third party material in this Peer Review File are included in the article's Creative Commons license, unless indicated otherwise in a credit line to the material. If material is not included in the article's Creative Commons license and your intended use is not permitted by statutory regulation or exceeds the permitted use, you will need to obtain permission directly from the copyright holder.
To view a copy of this license, visit https://creativecommons.org/licenses/by/4.0/

Point-by-Point Response to Reviewers

Manuscript NCOMMS-26-022249
"Small-scale photonic Kolmogorov–Arnold networks
using standard telecom nonlinear modules"

Luca Nogueira Calçado, Egor Manuylovich, Sergei K. Turitsyn

May 8, 2026

We would like to thank the reviewers for their detailed and constructive comments. We believe that addressing these insightful comments helped us to make presentation more clear and this significantly strengthened the manuscript. Below we address each point in detail, with reviewer comments shown in blue italics and our responses in green.

Reviewer #1

Essential criticisms

Comment 1(a): Distinction from prior photonic KAN implementations

The distinciton between small-scale photonic Kolmogorov–Arnold networks and prior photonic multilayer perceptron implementations is well explained. However, the difference with respect to recent photonic Kolmogorov–Arnold implementations (e.g., ring-assisted Mach–Zehnder interferometer-based systems) should be articulated more explicitly.

Our response: The revised Introduction now includes a more explicit comparison with recent photonic KAN implementations, including the ring-assisted MZI (RAMZI) architecture of Peng et al. and the self-phase modulation (SPM) approach of Sozos et al. Note that RAMZI relies on free-carrier dispersion in silicon microring resonators that must be custom-fabricated and precisely tuned, while the SPM approach requires engineered nonlinear waveguides with tailored dispersion. Beyond hardware accessibility, the key distinction of SSP-KAN is the use of differentiable, physically grounded telecom nonlinearities implemented entirely with commercially available fibre-pigtailed components (SOAs, VOAs, MZI couplers) , enabling direct experimental realisation without custom fabrication. We have made corresponding revision and clarification in the Introduction, lines 92–110.

Comment 1(b): Experimental feasibility

The present study is entirely simulation-based (albeit using realistic parameters). Additional discussion of experimental feasibility would strengthen the manuscript.

Our response: The revised Discussion identifies every MZI-VOA-SOA-VOA component by commercial part number and confirms that a prototype can be assembled from catalogue fibre-pigtailed devices with no custom fabrication or cleanroom access. The two primary practical challenges, SOA characterisation and interferometric phase stability, are addressed in Supplementary Section E: the Agrawal–Olsson parameters can be extracted from standard P_{out} vs P_{in} sweep across the 600–1700 mA bias range, and phase stability is mitigated by TEC stabilisation and low-bandwidth feedback locking, which are standard techniques in coherent optical systems.

Trained parameters are clamped to the datasheet operating ranges during optimisation so all set-points are directly realisable. The [2,2] Two Moons classifier (four modules) is identified as the minimal experimental target. See revised Discussion, lines 713–717.

Comment 1(c): Practical challenges

In the Discussion section, the authors identify "primary practical challenges" as (a) semiconductor optical amplifier characterisation and (b) phase stability. A more detailed analysis of these issues would be valuable.

Our response: This is addressed jointly with Comment 1(b). The revised Discussion gives a summary, and Supplementary Section E contains the full analysis: SOA characterisation requires only a standard P_{out} vs P_{in} sweep at several bias currents to extract the three Agrawal–Olsson parameters (P_{sat}, G_{max}, I_{tr}), with a fine-tuning pass to compensate residual model–device mismatch. Interferometric phase stability is a known challenge for fibre-based MZI systems, and the standard mitigation techniques (TEC stabilisation, low-bandwidth feedback locking, and path-matched design) used routinely in coherent optical communications apply directly to the 4–7 module configurations targeted in this work.

Comment 1(d): Minimal experimental implementation and training strategy

It would be helpful to outline a minimal experimental implementation and to specify expected stability requirements (e.g., phase drift tolerance and calibration frequency). In addition, the authors should clarify whether training is intended to be performed in situ or offline, how trained parameters can be transferred to hardware, and how sensitive performance is to parameter mismatch.

Our response: We have added a new Methods subsection ("Training and deployment strategies") addressing each aspect. Training is performed entirely offline via backpropagation through the differentiable physics model; set-points are then transferred to hardware by programming current sources, VOA drivers, and phase controllers directly, with no in-situ gradient computation. For scenarios in which the differentiable model is insufficiently accurate, we describe three gradient-free on-site optimisers (CMA-ES, PEPG, SPSA) that operate on forward-pass evaluations only; CMA-ES results for both the [2,2] Two Moons and [6,1,1] yacht configurations are presented in Supplementary Section F. The trained parameter values for all experimentally realisable configurations are tabulated in Supplementary Section C. Sensitivity to parameter mismatch is bounded by the existing robustness analysis: mismatch acts as an effective transfer-function perturbation analogous to the additive noise already characterised, with performance remaining above 90% on Two Moons and $R^2 > 0.79$ on yacht hydrodynamics at SNR = 14 dB. Phase stability is mitigated by TEC stabilisation and low-bandwidth feedback locking (standard in coherent optical systems), with periodic recalibration at the minutes-to-hours timescale sufficient for sustained laboratory operation.

Comment 2: Scalability

The authors correctly note that large-scale architectures (e.g., MNIST with $\sim 10^4$ modules) are impractical in fibre-based implementations. However, the discussion of scalability could be made more forward-looking. In particular, it would be useful to comment on whether performance gains saturate with network depth and width, to outline possible integration pathways, and to discuss scaling limits imposed by loss, noise accumulation, and phase stability.

Our response: We have added two paragraphs to the Discussion addressing each aspect. First, a scaling analysis across all task families shows that depth provides substantial benefit when feature interactions are required (yacht R^2: 0.921 ± 0.017 to 0.986 ± 0.015 from [6,1] to [6,1,1],

10 seeds), while width scaling on MNIST saturates rapidly, indicating that the four-parameter activation family rather than network capacity becomes the limiting factor. Enhancing per-module expressivity is therefore the productive scaling direction, not increasing module count. Second, we analyse the physical scaling limits: fan-out loss ($P_{\text{in}}/n_{\text{out}}$ per module), cumulative ASE noise from incoherent summation, and phase stability overhead scaling linearly with module count. Physics-based noise modelling (Supplementary Section D) shows that output OSNR remains above 24 dB for all architectures tested, well above the 14 dB empirical threshold from the robustness analysis. The 4–7 module configurations targeted for near-term demonstration are well served by fibre-coupled catalogue components; photonic integration becomes relevant only for architectures requiring hundreds of modules. See revised Discussion, lines 663–690.

Comment 3: Noise model justification

The authors adopt a power-domain noise model for the interpretation of the amplified spontaneous emission. While reasonable, this constitutes a simplification; furthermore, the amplitude-domain model is mentioned but not explored. The authors should justify more explicitly why the power-domain model is sufficient, and comment on whether the conclusions would change in the presence of signal-dependent noise or phase noise, which may be particularly relevant for interferometric operation.

Our response: Three concerns are raised here: sufficiency of the power-domain model, signal-dependent noise, and phase noise. All are addressed by new physics-based analysis in Supplementary Section D and a Methods paragraph that validates the phenomenological model against the Thorlabs BOA1554P datasheet noise figure (NF = 6–8 dB, $n_{\text{sp}} \approx 2$–3.15). Supplementary Experiment 1 compares four noise models (power-domain, amplitude-domain, datasheet NF, first-principles ASE) across three architectures and two tasks; both physics-based models produce accuracy within one standard deviation of the clean baseline. The phenomenological 14 dB SNR threshold corresponds to roughly $10^2\times$ more noise than the actual ASE floor, so the power-domain model is a conservative stress test rather than an overestimate. Signal-dependent noise is characterised explicitly in Experiment 4: sig-ASE beat variance scales as $\sigma^2 \propto P_{\text{signal}}$, consistent with heterodyne-beat theory. Output distributions after cascaded nonlinear propagation remain near-Gaussian (skewness < 0.3, excess kurtosis < 0.3; Experiment 2), so SOA saturation does not introduce significant non-Gaussian distortion, and cumulative OSNR exceeds 24 dB for every architecture tested (Experiment 3). Laser phase noise converts to intensity noise at the MZI output on timescales set by the laser coherence time ($\sim$10 μs for standard telecom sources), much longer than any inference interval; it therefore acts as slow set-point drift rather than per-sample noise, and is absorbed by the periodic recalibration protocol described in Supplementary Section E.

Secondary points

Comment S1: Training vs. deployment and hardware-in-the-loop

The authors should clarify the relationship between training and deployment, and indicate whether training assumes perfect knowledge of device parameters. Is hardware-in-the-loop training required in practice?

Our response: Training and deployment are separate stages: offline training produces per-module parameters $(I, \alpha_1, \alpha_2, \varphi)$ via backpropagation through the differentiable physics model, and deployment programs those set-points directly into current sources, VOA drivers, and phase controllers. The training does not assume perfect hardware; it assumes that the three device-level constants (P_{sat}, G_{max}, I_{tr}) are known from a standard P_{out} vs P_{in} characterisation sweep, which is routine for SOAs. Residual model–device mismatch is absorbed by a fine-tuning pass that re-optimises the four trainable parameters with the measured constants substituted into

the forward model, without full retraining. Hardware-in-the-loop training is not required, but is available as an on-site option through the three gradient-free optimisers (CMA-ES, PEPG, SPSA) described in the new Methods subsection and the CMA-ES results in Supplementary Section F. See also our response to Comment 1(d).

Comment S2: Energy per inference and transfer function discussion

The study highlights a reduced number of parameters compared to software Kolmogorov–Arnold networks. It would be useful to include an estimate of the energy per inference, or at least a qualitative comparison with electronic neural networks. In addition, the transfer function (equation (15)), which is central to the work, is somewhat understated; further discussion would help to highlight its importance, particularly in relation to nonlinear science.

Our response: We have added an energy analysis subsection to the Methods. The BOA1554P is a high-power booster amplifier designed for long-haul transmission; SSP-KAN does not require its 35 dB gain and 18 dBm saturation power. At the typical trained operating point ($I \approx$ 1200 mA, $V \approx 1.5$ V), each BOA1554P dissipates ∼1.8 W, giving ∼7.2 nJ per inference for the [2,2] configuration at 1 GHz. A low-power SOA with $G_{\max} \sim$ 10–15 dB would draw ∼50 mW per device, reducing the [2,2] total to ∼200 pJ per inference at 1 GHz — comparable to the bare electronic estimate (∼28–140 pJ for 28 MACs on 45 nm CMOS [Horowitz 2014, Sze 2017]). The BOA1554P therefore sets an upper bound; the actual cost scales with device selection. The advantage is not energy but latency (a single analogue pass at group-velocity speed) and native optical-domain operation without OEO conversion. See revised Methods, "Energy analysis" subsection.

Regarding the transfer function: Eq. (15) is the central result of the module design. The output power P_{out} depends on all four trainable parameters through a Mach–Zehnder interference fringe whose visibility, position, and curvature are modulated by SOA gain saturation. The gain $G = e^h$ is itself an implicit function of input power through the transcendental steady-state equation (Eq. (6)), while the gain–phase coupling $\Delta\varphi_{\text{SOA}} = -\alpha_H h/2$ links amplitude saturation to phase modulation. This nonlinear amplitude–phase coupling is the source of the functional diversity shown shown in Fig. 1(b): the four parameters control not only the output magnitude but also the shape of the nonlinear mapping, producing monotonic, non-monotonic, and multi-valued regimes within a single compact device.

Editorial comments

Comment E1: Label trainable parameters in Fig. 1

The authors should consider explicitly labelling the "trainable parameters" in the diagram of Fig. 1.

Our response: Figure 1 has been updated to label the four trainable parameters (I, α_1, α_2, φ) directly on the module schematic in panel (a).

Comment E2: Notation consistency

The authors should ensure consistency of notation. For instance, the same notation φ is used for a function in equation (2) and for a phase in sections 3.6–3.7.

Our response: The KAN edge functions in Eqs. (2) and (7) have been renamed from φ to φ throughout, disambiguating them from the interferometric phase φ used in Sections 3.6–3.7 and in the module transfer function of Eq. (15). The change is applied consistently in Fig. 1(c) and in all references to the per-edge transfer functions $\varphi_{i,j}$.

Comment E3: Missing reference

Line 520: a reference is missing.

Our response: The missing reference has been identified and corrected. All citations now resolve in the compiled manuscript.

Comment E4: Additional references

The paper is already well referenced, but I would suggest to consider mentioning some recent publications, e.g. Feldmann et al., Nature 589, 52–58 (2021); Xu et al., Nature 589, 44–51 (2021); Miscuglio & Sorger, Appl. Phys. Rev. 7, 031404 (2020).

Our response: All three references have been added to the Introduction, cited as representative recent demonstrations of photonic neural-network accelerators: Feldmann et al. for parallel convolutional processing on integrated photonic tensor cores, Xu et al. for the 11 TOPS photonic convolutional accelerator, and Miscuglio & Sorger for photonic tensor-core architectures targeting general machine-learning workloads.

Reviewer #2

Comment 1: Novelty and originality

The article is based on the Kolmogorov–Arnold network (KAN), but the KAN framework itself is not the author's original creation. The innovation of this work is mainly in the use of optical modules (MZI, VOA, SOA) to implement KAN, but similar ideas of using optical nonlinear elements to build neural networks have been studied in advance [Peng et al., Sozos et al.]. The innovation of this paper is mainly reflected in the engineering implementation level, rather than proposing a completely new architecture or theory. Therefore, at the scientific level, there is a lack of substantial expansion of the KAN model or learning method.

Our response: The revised Introduction addresses this directly through an explicit comparison with both the RAMZI architecture of Peng et al. and the self-phase modulation approach of Sozos et al.. We respectfully differ from the framing that SSP-KAN is only an engineering variant. The two prior photonic KAN implementations rely on specialised optical mechanisms (free-carrier dispersion in silicon microrings; Kerr nonlinearity in engineered waveguides) that constrain the set of physically realisable transfer functions and require custom fabrication. Demonstrating that SOA gain saturation combined with interferometric mixing also supports KAN inference — with only four trainable parameters per edge and commodity telecom components — is a substantive finding about the expressivity of physically constrained activation families rather than a pure engineering integration. The differentiable physics model (Methods) is itself a contribution: it enables gradient-based co-design of network architecture and optical hardware, and extends to any nonlinear optical platform with analytically tractable transfer functions. See also our response to R1 Comment 1(a).

Comment 2: Ultrafast inference claim and temporal dynamics

The manuscript models the SOA response in the steady state and derives a static transfer function for each MZI-VOA-SOA-VOA module, which is then used for end-to-end training and inference. However, "ultrafast inference" is an architectural claim that cannot be supported by steady-state simulations alone, because it depends on the temporal response of the SOA, the recovery dynamics, the modulator bandwidth, the photodetector response, and any inter-symbol or inter-sample memory effects. The current analysis does not address pattern dependence, transient gain saturation, or time-domain settling behavior, all of which are critical for determining

whether the device can truly operate at high speed in realistic conditions.

Our response: The manuscript does not make an ultrafast-inference claim for SSP-KAN. References to speed and bandwidth in the Introduction and Discussion (application context, optical channel equalisation) describe general motivation for photonic inference, not empirical operating rates of the reported configurations. The steady-state Agrawal–Olsson model assumes $dh/dt = 0$, which is valid only for electrical observation bandwidths $B_e \ll 1/\tau_c$. For the BOA1554P with $\tau_c \approx 200$–500 ps, this gives an upper bound of roughly 1–2 GHz; Supplementary Section D makes this CW validity bound explicit. Within this bound, pattern dependence and transient gain recovery contribute to intra-symbol distortion rather than Gaussian noise and are not captured by the steady-state model. For the 4–7 module near-term targets of this work, operation well below the CW bound is consistent with all performance claims. Extending SSP-KAN to the multi-GHz, pattern-limited regime would require a time-dependent carrier model and is left to future work.

Comment 3: Optical loss budget

The optical loss budget and system-level efficiency are not quantified. Although the manuscript correctly notes that OEO conversion is a bottleneck in conventional photonic neural networks, the proposed architecture does not eliminate optical loss; rather, it redistributes it across MZI couplers, VOAs, SOAs, and photodetection-based summation.

Our response: The optical loss budget is implicit in the transfer function (Eq. (15) of the main text). Each component introduces a quantifiable loss or gain:

- Input fan-out: $-10\log_{10}(n_{\text{out}})$ dB per feature. For [2,2]: -3 dB; for [6,1]: 0 dB (single output node).
- 50/50 coupler (input): -3 dB per stage.
- VOA_1: 0 to -30 dB (trainable).
- SOA: up to $+35$ dB gain (compensates upstream losses).
- VOA_2: 0 to -30 dB (trainable).
- 50/50 coupler (output): -3 dB.
- Output summation coupler: -3 dB for [2,2] ($n_{\text{in}} = 2$).

The SOA provides net gain that compensates passive losses at each stage. For the [2,2] network, the total passive loss from input to output is approximately 12 dB (two fan-out stages plus two couplers), which the SOA compensates with 15–35 dB gain depending on bias. The trained VOA values (Supplementary Section C) show that the optimiser converges to low attenuation on VOA_1 (0.3–3 dB) and moderate attenuation on VOA_2 (0.1–8 dB), confirming that the network preserves sufficient optical power throughout. The OSNR analysis in Supplementary Section D confirms that output signal power exceeds the ASE noise floor by at least 24 dB for all architectures tested.

Comment 4: Noise propagation and depth–distortion trade-off

The manuscript observes that deeper architectures can improve expressivity but may also amplify noise. However, this trade-off is not analyzed in depth. In particular, nonlinear systems such as SOA-based modules can introduce distortion that is not simply additive noise, but signal-dependent and potentially non-Gaussian. When cascaded across layers, such distortions may accumulate in a highly nontrivial manner. A more detailed study of noise propagation would provide valuable insight into the limits of depth and the optimal operating regime.

Our response: The revised manuscript addresses this through the physics-based noise propagation analysis in Supplementary Section D. Output distributions after cascaded propagation

through the MZI-VOA-SOA-VOA modules remain near-Gaussian (skewness < 0.3, excess kurtosis < 0.3; Experiment 2), so SOA saturation does not generate significant non-Gaussian distortion at physical noise levels. Signal-dependent variance is present and characterised explicitly: the sig-ASE beat term gives $\sigma^2 \propto P_{\text{signal}}$ (Experiment 4), consistent with heterodyne-beat theory. Cumulative OSNR as a function of depth is tabulated in Experiment 3 and exceeds 24 dB for every architecture tested, including the [2,2,2] and [6,1,1] cascaded configurations, well above the 14 dB empirical threshold from the robustness analysis. A layer-by-layer cascade probe through a 7-layer network confirms that SNR decreases from ∼67 dB at the input to ∼49 dB at depth 7, remaining above the 14 dB threshold by more than 35 dB (Supplementary Fig. 5). The optimal operating regime is the gentle-saturation region, where the SOA provides sufficient nonlinearity without driving n_{sp} upward through deep saturation. See also our response to R1 Comment 3.

Comment 5: Task complexity

The validation tasks may not fully support the claimed generality of the approach. The manuscript evaluates the system on standard benchmarks such as Two Moons, yacht hydrodynamics, and MNIST/Fashion-MNIST. While these are useful for initial validation, they are relatively low-complexity tasks compared to modern machine learning workloads. For example, MNIST is known to be nearly linearly separable, and the observed performance gains over linear models are modest.

Our response: We thank the reviewer for this insightful comment. The benchmarks were chosen to validate the proposed hardware architecture and differentiable training framework, and to demonstrate feasibility on non-trivial nonlinear inference tasks, rather than to compete with state-of-the-art deep networks on large-scale benchmarks. This positioning is explicit in the title ("*Small-scale* photonic Kolmogorov–Arnold networks") and in the Discussion, which distinguishes near-term experimental targets (Two Moons [2,2] and yacht [6,1,1], 4–7 modules, directly realisable on an optical bench) from the MNIST-scale scaling studies that characterise performance trends rather than propose practical systems. On the modest MNIST margin: the near-linear separability of the dataset is acknowledged in the Results, and the advantage widens on the harder Fashion-MNIST benchmark (+2.4 versus +1.7 percentage points), consistent with the interpretation that the nonlinearity is doing useful work when nonlinearity is required. Two additional, more strongly nonlinear benchmarks are in preparation and will be included in the final revision. A natural scaling path complementary to the pure-optical design is a mixed electronic–optical network, in which linear matrix-vector multiplications are performed electronically (where digital precision and density are cheap) while the per-edge nonlinear activations are realised optically as MZI-VOA-SOA-VOA modules. This reserves the photonic hardware for the operation that benefits most from analogue implementation — trainable, latency-limited nonlinearity — and exploits mature electronic linear algebra for the bulk of the computation; it is a concrete route to closing the task-complexity gap and will be added to the Discussion. See also our response to R3 Comment 5.

Comment 6: Robustness to device variability

Since the architecture relies on cascaded nonlinear transfer functions, even small parameter perturbations may accumulate across layers and significantly alter the learned mapping. The manuscript should therefore include a robustness study under device variability.

Our response: We thank the reviewer for raising this point. Investigating it led us to a concrete improvement in the training procedure.

A dedicated device-variability study is now presented in Supplementary Section G. It evaluates 120 training configurations (8 perturbation conditions × 3 benchmark/mode combinations × 5 seeds) with variability-aware training, and 6000 Monte Carlo evaluations of nominally trained

models under device variation. During this study, multi-seed evaluation revealed that one seed consistently converged to a narrow minimum that was catastrophically sensitive to device variation, particularly α_H. Root-cause analysis showed that the failure originated not from the device parameters themselves but from a parameter-scale mismatch in the softplus-and-clamp parameterisation: the raw optimiser variables could reach scales where small perturbations to device constants destroyed the learned interference condition. This finding motivated the sigmoid reparameterisation ($\theta = \theta_{\min} + \sigma(z)(\theta_{\max} - \theta_{\min})$), which constrains each trainable parameter to its physical range through a smooth bijection and eliminates the initialisation-dependent failure mode.

With sigmoid reparameterisation, all eight variability conditions produce $R^2 \geq 0.955$ on yacht [6,1,1] under variability-aware training (Supplementary Table 8), compared with $R^2 = 0.778$ for the α_H condition and $R^2 = 0.839$ for the combined condition in the original parameterisation. Without variability-aware training, the linewidth enhancement factor α_H remains the dominant sensitivity: it enters the MZI phase through $\Delta\varphi_{\text{SOA}} = -\alpha_H h/2$, and moderate manufacturing variation still destroys the interference condition. The mitigation is to characterise α_H per device from a standard gain–phase measurement, then substitute the measured value before training. See also our response to R3 Comment 6.

Comment 7: Variability measures, energy, and footprint

The results are presented mostly as single accuracy or R^2 values without measures of variability. At the device or chip level, what about energy and footprint, such as energy per inference and compute density?

Our response: All three concerns are now addressed. *Variability:* all main-text results are reported with interquartile ranges across 10 random seeds (94.3% (IQR: 90.3–97.4%) for Two Moons [2,2]; $R^2 = 0.986 \pm 0.015$ for yacht [6,1,1]). The Supplementary noise experiments (Section D) likewise report multi-seed statistics. *Energy:* a new Methods subsection ("Energy analysis") estimates ∼7.2 nJ per inference for the [2,2] configuration at 1 GHz readout using the BOA1554P, which is a high-power booster amplifier overspecified for SSP-KAN. A low-power SOA with $G_{\max} \sim$ 10–15 dB would draw ∼50 mW per device, reducing per-inference energy to ∼200 pJ for [2,2], comparable to the bare electronic estimate (∼28–140 pJ for 28 MACs). The BOA1554P therefore sets an upper bound; the actual cost scales with device selection. *Footprint:* the [2,2] configuration requires four fibre-pigtailed MZI-VOA-SOA-VOA modules, each approximately 15 cm in fibre path length, fitting on a standard optical breadboard (∼ 60 × 90 cm). Photonic integration is relevant for architectures exceeding ∼100 modules but is beyond the scope of the present work.

Comment 8: Wavelength dependence

The impact of wavelength dependence and spectral effects is not considered. The model implicitly assumes a single-wavelength (or wavelength-independent) operation, but in practical telecom systems, components such as SOAs and MZIs exhibit wavelength-dependent gain, phase response, and loss. This becomes particularly relevant if the architecture is extended to wavelength-division multiplexing scenarios, which are a natural direction for scaling photonic neural networks.

Our response: The simulations assume operation at a single wavelength near the SOA gain peak (1550 nm). The BOA1554P datasheet specifies a gain bandwidth of approximately 80 nm (1520–1600 nm), with gain variation of ∼3 dB across this range. Wavelength-dependent effects enter through three mechanisms: (i) the SOA gain $G(\lambda)$ and noise figure $\text{NF}(\lambda)$ vary across the C-band; (ii) the MZI phase φ acquires a wavelength-dependent offset from chromatic dispersion in the fibre arms; (iii) the VOA attenuation is weakly wavelength-dependent (<0.5 dB variation

across the C-band for typical MEMS-based devices). For single-wavelength operation at the gain peak, these effects are negligible and are absorbed into the device characterisation.

Wavelength-dependent gain variation is a form of device parameter variability. A systematic variability study (Supplementary Section G) sweeping per-module $G_{\max}$, P_{sat}, I_{tr}, and α_H (8 conditions $\times$ 5 seeds) shows that when each module is characterised individually and trained with its own measured parameters, the network compensates $\sim$3 dB gain variation (the same order as the C-band gain slope). The linewidth enhancement factor α_H is the dominant sensitivity: it enters the interference condition through the gain–phase coupling $\Delta\varphi_{\text{SOA}} = -\alpha_H h/2$, so per-device α_H variation shifts the trained MZI phase set-points. This finding informs the WDM scaling path: per-channel training with wavelength-resolved Agrawal–Olsson parameters is required. We acknowledge this as future work in the revised Discussion.

Reviewer #3

Comment 1: Expressive power with only four parameters

The authors employ a single MZI-VOA-SOA-VOA module to realize nonlinear functionalities for the KAN. However, as with prior photonic KAN studies, this module contains only a very limited number of tunable parameters. It remains unclear how these four parameters can effectively implement nonlinear functions that typically require hundreds of learnable coefficients—such as those in B-spline representations. Figure 1(b) displays a family of curves but does not constitute sufficiently compelling evidence on its own. Rigorous mathematical proof is essential to substantiate the claimed nonlinear trainability of the proposed module.

Our response: Universal approximation in Kolmogorov–Arnold Networks is a network-level property, not a per-edge requirement. The Kolmogorov–Arnold representation theorem [Liu 2024] states that any continuous multivariate function can be written as a finite sum of compositions of univariate functions; it does not require each univariate function to be individually a universal approximator. B-splines with $G + k$ coefficients per edge [Liu 2024] are one realisation of sufficient per-edge flexibility, not a necessary condition. SSP-KAN replaces the B-spline family with the four-parameter MZI-VOA-SOA-VOA transfer function (Eq. (15)), parameterised by $(I, \alpha_1, \alpha_2, \varphi)$, which defines a structured continuous family of nonlinearities. Whether this family is dense in an appropriate function space is an open theoretical question that we do not resolve here; a density or approximation-rate theorem for the SSP-KAN activation family is natural future work. What the present results establish empirically is that compositional depth compensates for restricted per-edge expressivity: on yacht hydrodynamics, the single-layer SSP-KAN [6,1] reaches $R^2 = 0.921 \pm 0.017$ (six independent univariate modules, 10 seeds), while adding a second layer ([6,1,1], 28 parameters) improves to $R^2 = 0.986 \pm 0.015$, exceeding the pyKAN B-spline baseline ($R^2 = 0.977$, 60 parameters) with fewer than half the parameters. The revised scaling analysis identifies per-module expressivity as the dominant constraint at larger scale, consistent with the compositional interpretation. We respectfully suggest that this empirical evidence, together with the Kolmogorov–Arnold framework, is the appropriate standard of substantiation for a physical-layer demonstration; a rigorous mathematical proof of completeness is a separate, complementary theoretical programme.

Comment 2: Advantage over MLPs given deployment constraints

The authors acknowledge that realistic deployment requires 8-bit DAC resolution and SNR $\geq$ 14 dB. These are non-trivial requirements that demand significant power budgets in both the electronic and optical domains. This raises a fundamental question: does the proposed SSP-KAN offer a genuine advantage over conventional MLPs when the stringent deployment constraints

and associated energy consumption are taken into account? A fair and thorough comparison should be provided.

Our response: The new Methods subsection ("Energy analysis") provides the requested comparison. The BOA1554P is a high-power booster amplifier overspecified for SSP-KAN. At the typical trained operating point, the [2,2] configuration draws ∼7 W across four SOAs, giving ∼7.2 nJ per inference at 1 GHz. However, SSP-KAN requires only sufficient gain saturation to produce a tunable nonlinear transfer function. A low-power SOA ($G_{\max} \sim$ 10–15 dB, ∼50 mW per device) would reduce per-inference energy to ∼200 pJ for [2,2] at 1 GHz, comparable to the bare electronic estimate (∼28–140 pJ for 28 MACs on 45 nm CMOS [Horowitz 2014, Sze 2017]). The BOA1554P sets an upper bound; the actual cost scales with device selection and WDM parallelism.

Three properties distinguish SSP-KAN from electronic MLPs: (a) single-pass latency at group-velocity speed (sub-nanosecond through the module), with no clock-cycle overhead; (b) the signal remains in the optical domain throughout, avoiding OEO conversion and matching the native format of coherent-communication and fibre-sensing data; (c) the differentiable physics model enables gradient-based co-design of any photonic architecture with analytically tractable transfer functions.

Comment 3: SNRw degradation at scale

The VOA-SOA-VOA configuration will inevitably degrade SNR due to amplified spontaneous emission (ASE) noise. This concern becomes increasingly severe as the SSP-KAN scales to larger network sizes. The authors should provide quantitative analysis and simulation results to assess whether SNR $\geq$ 14 dB remains achievable in realistic, large-scale implementations.

Our response: The revised manuscript provides this quantitative analysis. Supplementary Experiment 3 tabulates cumulative OSNR across six architectures from [2,1] through the cascaded [2,2,2], [6,1], and [6,1,1] configurations; the lowest OSNR in the set is 24.2 dB for [6,1] with the datasheet NF model, corresponding to an equivalent phenomenological SNR of 49.7 dB — more than 30 dB above the 14 dB threshold at which performance starts to degrade. For the MNIST-scale [784,20,10] network, fan-out to 20 first-layer edges reduces the per-module signal by roughly 13 dB; the new Discussion scaling paragraph analyses how ASE noise accumulates through incoherent summation but is compensated by per-stage re-amplification, so OSNR does not decay with depth in the way it would in a passive lossy cascade. The 14 dB threshold therefore remains achievable for every architecture studied here, including the cascaded multi-layer configurations. Practical large-scale deployment is bounded less by SNR than by phase stability and module count, which are treated separately in the scaling paragraph and in Supplementary Section E. See also our responses to R1 Comment 3 and R2 Comment 4.

Comment 4: Lack of experimental validation

This work is entirely simulation-based, with no experimental chip fabrication or characterization. Given the highly competitive standard of Nature Communications, a purely theoretical and simulation study of this nature is insufficient for publication in this venue.

Our response: We understand the reviewer's concern but respectfully disagree with the conclusion. Four considerations support the contribution of the present simulation study. First, Nature Communications regularly publishes photonic simulation and theoretical studies when the physics is validated against measured component data and the framework has experimental implications. Second, all component parameters used in this work come directly from commercial datasheets (Thorlabs BOA1554P for the SOA, standard catalogue specifications for couplers and VOAs); no hypothetical devices are invoked. Third, the differentiable physics model and the end-

to-end training framework are themselves contributions: they enable gradient-based co-design of any photonic architecture with an analytically tractable transfer function, and their correctness does not depend on chip fabrication. Fourth, the minimal experimental demonstration is identified explicitly: the [2,2] Two Moons classifier requires four fibre-pigtailed catalogue modules and is fully specified in Supplementary Section E (component list, cost estimate, mitigation strategies for SOA characterisation and phase stability); scaling to the [6,1,1] yacht configuration requires only three additional modules. The manuscript is positioned as the design-and-training study that precedes this near-term demonstration, not as a replacement for it. See also our responses to R1 Comments 1(b), 1(c), and 1(d).

Comment 5: Task complexity

The chosen tasks, such as Two Moons and MNIST, are well below the current frontier of photonic machine learning. The community expects demonstrations on more complex and contemporary benchmarks to substantiate claims of practical relevance and competitiveness.

Our response: This concern is addressed jointly with R2 Comment 5, which raises the same point. Briefly: the benchmarks were chosen to validate the physical architecture and the differentiable training framework, not to compete on contemporary ML tasks; this positioning is explicit in the title ("*Small-scale* photonic Kolmogorov–Arnold networks"). Two additional, more strongly nonlinear benchmarks are in preparation for the final revision. The revised Discussion also proposes a concrete scaling path — a hybrid electronic-linear + optical-nonlinear architecture — that reserves photonic hardware for the operations that benefit most from analogue implementation while using mature electronic linear algebra for the bulk of the computation. See our full response to R2 Comment 5.

Comment 6: Device-to-device variation

The SOA and VOA elements require integrated III-V components. While certain foundries, such as Tower Semiconductor, have demonstrated this capability, device-to-device variation poses a serious concern that has not been addressed. Unlike telecommunications applications where moderate gain variation is tolerable, the proposed architecture exploits the nonlinear characteristics of III-V devices, which requires highly uniform output responses across all SOA and VOA elements for a given set of tunable parameters. Failure to achieve this uniformity would necessitate complex and potentially impractical calibration procedures. The authors must address this issue explicitly.

Our response: We clarify that SSP-KAN in the form demonstrated here does not require a foundry-integrated III-V platform. The minimal experimental configuration uses commercially available, fibre-pigtailed SOAs (Thorlabs BOA1554P), VOAs, and directional couplers; each device is individually characterised and individually trained, so per-device variation is absorbed by per-module training rather than requiring foundry-level uniformity. The revised manuscript addresses device-to-device variation through three complementary mechanisms. First, each SOA is characterised individually with a standard P_{out} vs P_{in} sweep to extract its own $(P_{\text{sat}}, G_{\max}, I_{\text{tr}})$; training then uses these measured constants rather than assuming uniformity (new Methods subsection "Training and deployment strategies"). Second, residual model–device mismatch is absorbed by a fine-tuning pass that re-optimises the four trainable parameters per module with the measured constants substituted into the forward model. Third, when the model or the characterisation is insufficient, gradient-free on-site recalibration is available through CMA-ES; Supplementary Section F demonstrates that CMA-ES reaches outcomes statistically equivalent to gradient-based training on the [2,2] Two Moons and [6,1,1] yacht configurations, at a cost of a few thousand forward-pass evaluations.

A systematic device-variability study (new Supplementary Section G) now provides quantitative

evidence for these mechanisms. The study also prompted a concrete improvement: multi-seed evaluation under device variation revealed an initialisation-dependent failure mode in the original softplus-and-clamp parameterisation. Investigating the root cause led to the sigmoid reparameterisation ($\theta = \theta_{\min} + \sigma(z)(\theta_{\max} - \theta_{\min})$), which constrains each parameter to its physical range through a smooth bijection and eliminates the failure. With sigmoid reparameterisation, all eight variability conditions produce $R^2 \geq 0.955$ under variability-aware training (Supplementary Table 8). Without variability-aware training, the linewidth enhancement factor α_H remains the dominant sensitivity: it couples to the MZI phase through $\Delta\varphi_{\mathrm{SOA}} = -\alpha_H h/2$, and moderate manufacturing variation (nominal $\alpha_H = 5$, sampled in [3, 7]) destroys the interference condition. Per-device α_H characterisation from a standard gain–phase measurement is the primary requirement for deployment. See also our response to R2 Comment 6.

Comment 7: Energy efficiency analysis

The proposed architecture raises significant concerns regarding energy efficiency that have not been addressed. The MZI-VOA-SOA-VOA module is inherently power-intensive, as nonlinearity comes at a direct energy cost. Given this, an energy efficiency analysis is essential. Specifically, the authors should provide a quantitative comparison between the proposed all-optical nonlinear approach and conventional O/E/O conversion methods in terms of total power consumption per operation. Without such an analysis, it is unclear whether the proposed architecture offers any meaningful energy advantage over existing approaches, undermining one of the key motivations for pursuing photonic implementations of neural networks.

Our response: The new Methods subsection ("Energy analysis") provides the requested quantitative comparison. The BOA1554P is a fibre-optic booster amplifier designed for high output power in long-haul transmission, with 35 dB gain and 18 dBm saturation power. SSP-KAN does not require these specifications. At a typical operating point ($I \approx 1200$ mA, $V \approx 1.5$ V), each BOA1554P dissipates ∼1.8 W. The [2,2] configuration draws ∼7 W across four SOAs, giving ∼7.2 nJ per inference at 1 GHz. However, SSP-KAN requires only sufficient gain saturation to produce a tunable nonlinear transfer function. A low-power SOA with $G_{\max} \sim$ 10–15 dB would draw ∼50 mW per device, reducing the [2,2] total to ∼200 mW and per-inference energy to ∼200 pJ at 1 GHz. This is comparable to the bare electronic estimate (∼28–140 pJ for 28 MACs on 45 nm CMOS [Horowitz 2014, Sze 2017]). The BOA1554P therefore sets an upper bound on per-inference energy; the actual cost scales with device selection.

SSP-KAN is not intended to compete with highly optimised electronic ASICs at small network scales. Rather, its advantages lie in single-pass latency, native optical-domain processing without repeated O/E/O conversion, and compatibility with wavelength- and space-division multiplexing architectures, where photonic parallelism becomes increasingly advantageous. Device selection and WDM parallelism are the primary levers for reducing per-inference energy. See also our responses to R1 Comment S2, R2 Comment 7, and R3 Comment 2.

Concluding remarks

We are grateful to the reviewers for their time and numerous helpful comments. We beiev that the revised manuscript has been substantially strengthened as a result. In response to the reviewers' comments, the revised manuscript includes: expanded comparison with and clarified the novelty of SSP-KAN relative to prior photonic KAN platforms; new detailed discussion of experimental feasibility and deployment strategies; new analyses of ASE noise propagation, wavelength dependence, and device variability; a new Methods subsection on energy consumption analysis; expanded comparison with electronic and photonic alternatives; clarification of training strategies and hardware transfer; expanded discussion of scalability and practical implementation constraints.

We hope that the revised manuscript and the accompanying point-by-point responses satisfactorily address all concerns raised by the reviewers.

Point-by-Point Response to Reviewers (Second Round)

Manuscript NCOMMS-26-022249A
"Small-scale photonic Kolmogorov–Arnold networks
using standard telecom nonlinear modules"

Luca Nogueira Calçado, Egor Manuylovich, Sergei K. Turitsyn

11 June 2026

We thank all three referees for the second round of review. Referees #1 and #2 are satisfied with the revised manuscript and recommend publication. Our responses therefore focus on the three numbered concerns of Referee #3. Reviewer comments are shown in blue italics and our responses in green. All changes to the manuscript and Supplementary Information are shown in blue tracked markup.

Reviewer #1

The authors have done a commendable job addressing the concerns raised by all reviewers. In particular, they carried out additional modeling efforts and improved several aspects of the presentation and analysis. Overall, I am satisfied with the thoroughness of the revision and recommend the manuscript for publication. I would also like to note that the questions concerning energy estimates, while certainly valid and important, are not specific to this work alone. Similar issues arise in many papers in the field, largely because the relevant analytical framework remains incomplete and no universally accepted methodology has yet been established.

Our response: We thank the referee for the positive assessment and the recommendation for publication. We agree that energy accounting for photonic neural hardware lacks an established methodology; for this reason the manuscript reports the BOA1554P figure (7.2 nJ per inference at 1 GHz) explicitly as an upper bound and states all assumptions in Methods.

Reviewer #2

The authors have addressed my concerns. I think it can be accepted now.

(Remarks on code availability) Yes. The code is well-structured, and the results are largely reproducible as described.

Our response: We thank the referee for the positive assessment of both the manuscript and the code.

Reviewer #3

Comment 1: Limited algorithmic novelty

The Kolmogorov-Arnold Network (KAN) framework itself is directly adopted from Liu et al. (arXiv:2404.19756, 2025) without modification. The authors place trainable univariate nonlinear functions on network edges following exactly the same computational graph structure as the

original KAN formulation. The sole architectural contribution is the substitution of B-spline activation functions with MZI-VOA-SOA-VOA transfer functions. While the authors argue in their response to Reviewer 2 Comment 1 that "demonstrating that SOA gain saturation combined with interferometric mixing also supports KAN inference is a substantive finding about the expressivity of physically constrained activation families," this argument conflates hardware implementation with algorithmic innovation. The expressivity analysis itself (Supplementary Section A, Fig. A1) reveals fundamental limitations: the single-layer [2,2] architecture fails completely on XOR (51.8%, chance level), demonstrating that the constrained four-parameter activation family introduces new failure modes not present in software KANs. These limitations are not adequately theorised; the authors acknowledge in their response to Reviewer 3 Comment 1 that "whether this family is dense in an appropriate function space is an open theoretical question," yet present no approximation-theoretic bounds or convergence guarantees.

Our response: We address the three parts of this comment in turn.

(a) The computational graph is adopted deliberately. We agree that the graph follows the original KAN formulation without modification. This is intentional. The objective is not to introduce a new KAN architecture but to test whether useful nonlinear inference can be achieved when the trainable activations are restricted to a four-parameter family dictated by the physics of commercially available telecom components. This is the question the manuscript poses and answers: the Introduction identifies as an open question "whether the constrained nonlinear transfer functions available from practical optical components (often defined by only a small number of tunable physical parameters) can achieve expressive capability comparable to software-based activation functions such as B-splines", and the Discussion opens with the same question. The contribution is a physics and hardware-computing result, not an algorithmic one.

Importantly, the scientific question addressed here cannot be answered from the original KAN formulation. Theoretical KANs assume highly flexible trainable edge functions, whereas physical implementations are restricted to transfer functions determined by device physics. The manuscript therefore investigates whether useful nonlinear inference survives when activation functions are constrained to a four-parameter family derived from standard telecom hardware, and quantifies the resulting computational trade-offs.

(b) The XOR result is not a new failure mode of the four-parameter family. No single-layer KAN can represent XOR, for any activation family, software or hardware. The output of a single additive layer is a sum of univariate functions, so the two-class discriminant reduces to $a(x_1) + b(x_2)$. Assigning one label to two diagonally opposite quadrants and the opposite label to the other two requires four inequalities whose pairwise sums force the same quantity to be simultaneously above and below a threshold, which is impossible. A single-layer B-spline KAN scores at chance on XOR for exactly the same reason, and depth restores performance in both cases (SSP-KAN [2,2]: 51.8%; [2,2,2]: 89.4%). This limitation is a structural property of the KAN graph itself, shared with the software KANs of Liu et al., rather than a property of the four-parameter activation family. The revised Supplementary Section A now contains this impossibility argument as a short formal proof (new equation and surrounding text, tracked), and the Discussion now states it in one sentence where the depth-scaling results are presented.

(c) Approximation-theoretic bounds. We agree these remain open and we do not claim them. The manuscript claims empirical sufficiency for the studied task classes, with the performance cost of the constrained family quantified against B-spline and RAMZI baselines, and identifies formal approximation rates for physically constrained activation families as future work. We therefore do not present SSP-KAN as a new learning theory, approximation theory, or neural-network architecture. Rather, the contribution is to establish experimentally (through physics-based simulation) that a severely constrained activation family remains sufficient for meaningful nonlinear inference across classification, regression, and image-recognition tasks.

Comment 2: Incremental differentiation from prior photonic KAN work

Compared to existing photonic KAN implementations, the primary distinction of SSP-KAN is the choice of nonlinear device rather than any new principle. Peng et al. (arXiv:2408.08407) demonstrated photonic KAN using ring-assisted MZI (RAMZI) achieving 98% MNIST accuracy with nine tunable parameters per edge. Sozos et al. (Optics Letters, 2026) implemented photonic KAN via self-phase modulation in nonlinear waveguides. The present work achieves 93.9% MNIST accuracy with four parameters per edge using SOA gain saturation. The authors frame the use of commodity telecom components as the key differentiator. However, the engineering trade-off between hardware accessibility and activation expressivity does not, in itself, constitute a conceptual advance sufficient for Nature Communications.

Our response: We do not claim a new principle of optical nonlinearity. The question the work answers is different from those addressed by Peng et al. and Sozos et al.: how far can KAN inference be pushed when the activation family is maximally constrained, to four physically grounded parameters realisable with catalogue fibre-pigtailed components, and what is the quantified cost of that constraint? The answer is concrete: 93.9% versus 98% on MNIST with four versus nine parameters per edge, $R^2 = 0.986$ on yacht regression exceeding the pyKAN baseline with fewer than half the parameters, and no custom fabrication. The observed performance difference is therefore not presented as a competition between devices, but as a quantitative measurement of the cost of physical constraints. We believe that understanding how computational performance degrades as activation functions become physically realizable is itself a question of broader relevance to analog and physical AI systems.

More fundamentally, the manuscript is not primarily a comparison of nonlinear devices. Prior photonic KAN demonstrations establish that KAN inference can be implemented using custom photonic platforms. SSP-KAN asks a complementary question: how much computational capability is retained when the activation family is restricted to the physics of widely available telecom components requiring no custom fabrication. In this sense, the work investigates the relationship between physical expressivity and computational performance rather than introducing another nonlinear mechanism.

Comment 3: Purely simulation-based validation

The authors argue that Nature Communications regularly publishes photonic simulation and theoretical studies when the physics is validated against measured component data. While this is true, the more pertinent question is whether the theoretical contribution itself is of sufficient significance to warrant publication in this venue. In the case of the present manuscript, the work amounts to a simulation study demonstrating the mapping of an existing algorithmic framework onto a specific class of optical hardware; no new theoretical results are derived, no approximation-theoretic guarantees are provided for the four-parameter activation family. Beyond the question of theoretical significance, several critical performance claims rest on assumptions that have not been experimentally verified and that, on closer inspection, are more fragile than the manuscript acknowledges. The SNR analysis in Supplementary Section D reports output SNR values of 49–67 dB across a 7-layer cascade under the CW steady-state assumption, which is valid only when the electrical observation bandwidth satisfies $B_e \ll 1/\tau_c \approx$ 2–5 GHz (Supplementary Eq. D19). The authors acknowledge this constraint but do not quantify how inference performance degrades as the operating speed approaches this bound. It is a gap that is critical for the latency and bandwidth claims that motivate the photonic implementation in the first place. More seriously, the device variability study in Supplementary Section G demonstrates that deploying a nominally trained model without per-device characterisation of the linewidth enhancement factor α_H results in catastrophic and systematic failure: mean $R^2 = -145$ on the yacht [6,1,1] task, with the 95th percentile of the Monte Carlo distribution remaining negative across all 250 device

draws (Supplementary Fig. G7). This is not an edge case but the expected outcome for any device whose α_H differs from the nominal training value of 5, a variation the authors themselves note is routinely ±40% across SOA product lines. The proposed mitigation, per-device α_H characterization via gain-phase measurement, is described only in simulation and has not been demonstrated experimentally. Until it is, the claim that SSP-KAN is practically deployable using commodity telecom hardware remains unsubstantiated.

Our response: We accept the referee's broader point that the study is simulation-based and that experimental validation remains future work; the manuscript positions itself accordingly ("small-scale" in the title, explicit experimental plan in Supplementary Section E, the four-module [2,2] configuration identified as the immediate experimental target). We respond to the two specific technical points.

(a) Operation near the CW validity bound. All performance and latency claims in the manuscript are confined to electrical bandwidths $B_e \lesssim 1$ GHz, where the steady-state Agrawal–Olsson model is self-consistent. Within this regime the dominant signal–ASE beat noise scales linearly with B_e (Supplementary Section D), so the noise is bounded to be small in exactly the regime where the model is valid; the claims and the model fail together at the boundary rather than the claims outrunning the model. We make no multi-GHz claims. Quantifying inference performance in the pattern-dependent regime beyond $1/\tau_c$ requires a transient gain model and is stated as future work. The manuscript deliberately avoids claims in this regime. The Discussion now contains two tracked sentences making this scope explicit.

(b) The $R^2 = -145$ result characterises blind deployment, not the proposed operating mode. The Monte Carlo condition behind this number deploys a nominally trained model on devices whose α_H is drawn from a ±40% range with no characterisation of any kind. The manuscript includes this condition to quantify why the calibration step is needed, not as a description of intended operation. In this sense, the result should not be interpreted as evidence against deployability, but rather as identification of a specific deployment requirement. The purpose of Supplementary Fig. G7 is precisely to distinguish between conditions under which SSP-KAN can and cannot be expected to operate reliably. Without such a stress test, the calibration requirement would remain hidden.

The deployment workflow proposed in the manuscript includes a per-device gain–phase measurement to determine α_H; when the measured value is supplied, inference is robust across the entire simulated range ($R^2 \geq 0.955$ for all eight perturbation conditions). Two further points bound the severity. First, the ±40% range spans different SOA product lines; within a given product family the spread is substantially smaller, so the stress test is deliberately conservative. Second, gain–phase characterisation of α_H is a standard measurement with decades of literature; performing it on the ordered devices is part of the planned experimental validation. Supplementary Section G and the corresponding Discussion paragraph have been revised (tracked) to present the variability study as identifying a calibration requirement rather than a fundamental obstacle to deployment.

Overall assessment

Overall, were this manuscript submitted to an engineering-focused venue such as Journal of Lightwave Technology, it would constitute a valuable and publishable contribution: simulation studies grounded in commercial device specifications are standard in these venues, and the engineering contribution of realizing photonic neural computation from off-the-shelf fibre-pigtailed components would receive appropriate recognition. For Nature Communications, however, where conceptual novelty and demonstrated real-world impact are the primary criteria, the manuscript currently falls short.

Our response: We respect that the venue threshold is an editorial judgement.We respectfully suggest, however, that the central contribution should be evaluated as a study of computation under physical constraints rather than as a new machine-learning architecture. The manuscript addresses a general question that extends beyond photonic KANs: how much computational functionality can be retained when highly expressive software primitives are replaced by physically realizable dynamical elements. This question is increasingly relevant across photonic, electronic, neuromorphic and analog-AI hardware platforms.

Our aim in this revision has been to make the contribution straightforward to assess: the manuscript tests whether a physically constrained activation family supports nonlinear inference, quantifies the cost of the constraint against B-spline and RAMZI baselines, and states the scope of every claim explicitly, with the planned minimal experiment specified down to catalogue part numbers (Supplementary Section E). New computing concepts frequently appear first as theory and simulation studies validated against measured component data; we have positioned the work in that tradition and leave the assessment of its significance to the editors.

We therefore believe the manuscript's significance does not rest on introducing a new KAN architecture or a new nonlinear optical effect. Rather, it lies in demonstrating and quantifying how useful nonlinear inference emerges from a highly constrained, physically realizable activation family built entirely from standard telecommunications hardware. Together with the robustness, scalability and deployment analyses added during revision, we believe this contribution is of interest to the broader community working at the intersection of computation, machine learning and physical hardware.

Summary of changes in this revision

All changes are shown in blue tracked markup in the manuscript and Supplementary Information.

1. **Discussion (depth scaling):** added the structural impossibility of XOR for any single-layer additive KAN, software or hardware (responds to R3 Comment 1).
2. **Discussion (noise analysis):** added explicit confinement of all rate-dependent claims to $B_e \lesssim 1$ GHz and identified the transient regime as future work (responds to R3 Comment 3a).
3. **Discussion (variability):** reworded the device-variability paragraph to present per-device α_H characterisation as the operating mode, with blind deployment quantifying why it is needed (responds to R3 Comment 3b).
4. **Supplementary Section A:** formal proof that no single-layer additive KAN represents XOR, with a new equation; figure caption updated (responds to R3 Comment 1).
5. **Supplementary Section G:** blind-deployment scenario reframed as a calibration requirement; added the conservatism of the $\pm 40\%$ α_H stress range and the role of the standard gain–phase measurement (responds to R3 Comment 3b).
